# An Ecological Study on the Mortality Impact of the COVID-19 Pandemic According to Country Development Status and Pandemic Years

**DOI:** 10.3390/epidemiologia7020050

**Published:** 2026-04-06

**Authors:** Murat Razi, Manuel Graña

**Affiliations:** Computational Intelligence Group, University of the Basque Country UPV/EHU, 20018 San Sebastian, Spain; mrazi001@ikasle.ehu.eus

**Keywords:** COVID-19, accumulated mortality ratio, ecological study, aging population, obesity prevalence, diabetes prevalence, Gini index, Democracy Index, socioeconomic determinants, excess mortality

## Abstract

The COVID-19 pandemic caused stark global mortality disparities, influenced by a complex interplay of demographic, economic, and health factors. This ecological study investigates associations between country macroscopic variables and COVID-19 accumulated mortality ratio (AMR) across 174 countries and may serve as a preparation for new pandemics. Methods: The study applies bidirectional stepwise multiple linear regression. To ensure statistical validity, we conducted diagnostic tests for multicollinearity and heteroscedasticity, applying robust M-estimation where necessary to minimize root mean squared error. The analysis covered six distinct stratifications based on development status (developed, developing, least developed, and combinations), and incorporated temporal analyses across three specific annual periods: 21 January 2020–20 January 2021; 21 January 2021–20 January 2022; and 21 January 2022–10 January 2023. Data: AMR per country values, accumulated between 21 January 2020 and 10 January 2023, and data on the prevalence of health conditions, and socioeconomic descriptive variables were extracted from Our World in Data (OWID) and other public data sites, like the World Bank. Results: The percentage of population aged over 65 years has the most consistent association with increased AMR globally. Obesity prevalence and income inequality (Gini index) were positively associated with AMR regardless of country development status. Conversely, the study finds a consistent negative correlation with diabetes prevalence, while the prevalence of respiratory diseases is a significant association only for developed nations. Socioeconomic factors were significantly associated with AMR, but this influence is stronger in developed countries than in the developing and least developed countries. Conclusions: While population aging is the primary association with increased AMR, the mortality impact of comorbidities and socioeconomic factors is heavily conditioned by a country’s development stage, pointing to the necessity of development-status-aware public health strategies for incoming pandemics.

## 1. Introduction

The Coronavirus Disease 2019 (COVID-19) has been a disruptive global public health crisis with wide-reaching consequences across healthcare systems and socioeconomic structures, casting a fearsome premonition for incoming pandemics in the future. The pandemic resulted in stark disparities in health outcomes, specifically in the COVID-19 accumulated mortality ratio (AMR), both across different nations and within their populations. While the crisis unfolded as a syndemic—where the impact was intensified by existing health vulnerabilities and unequal socioeconomic conditions [1,2]—identifying the specific macroscopic variables associated with these disparities remains an urgent task for effective risk assessment and policymaking in the face of the next pandemic.

Extensive research has attempted to identify the macroscopic variables influencing COVID-19 AMR. Demographic structure, particularly the percentage of population of advanced age, has been consistently identified as a primary public health risk factor, given the association between aging and weakened immune responses [3,4,5,6,7]. Conversely, some studies have found a negative correlation between population spatial distribution, i.e., population spatial density, and the AMR [8,9].

Economic indicators, such as gross domestic product (GDP) per capita and unemployment rate, reflect a country’s overall wealth and economic capacity. These indices explain how well a country can fund healthcare systems, emergency response mechanisms, and provide social support during health crises. Wealthier countries should have better resources but at the same time may face challenges related to population aging or urban density. Previous studies found that the mortality was positively associated with GDP [7,10,11,12], and that COVID-19 AMR and unemployment rate are positively associated inside USA [13,14].

Socioeconomic indicators, such as the Human Development Index (HDI), the Inequality-adjusted HDI (IHDI), and the Gender Inequality Index (GII) represent dimensions of national development, equity, and access to healthcare and education. These variables help explain differences in a country’s ability to respond effectively to public health crises and to protect vulnerable populations. Some previous studies show that the number of COVID-19 deaths was negatively associated with HDI [9,15,16], while others found that the socioeconomic indexes (HDI, IHDI, GII) had no significant correlation with COVID-19 incidence and AMR except in Western Pacific Region Countries (WPRO) [17]. Inequality, especially in the form of income deprivation and economic disparity, played a crucial role in shaping the global impact of COVID-19 [2].

The most relevant political indicators are the Democracy Index, which reflects the nature of political governance, and the Gini coefficient, which measures income inequality. These structural factors give information about the population trust in institutions, the efficiency of policy implementation, and the equity of healthcare delivery in the country. Previous studies found that AMR was positively associated with the Gini index in developed countries [18,19,20,21,22]. While poverty indicators have weak effects globally, some studies report that relative poverty is a strong predictor of COVID-19 case incidence specifically within OECD countries, suggesting that economic deprivation within wealthy nations remains a potent driver of transmission [21].

While democratic institutions are generally associated with better public health outcomes, several studies have reported a counterintuitive positive correlation between democracy indices and COVID-19 case incidence [23] and case fatality rate [12]. This situation has been attributed to several factors: (1) the aging demographic structure typical of wealthy democracies [12], (2) higher data transparency and accurate reporting compared to authoritarian regimes that may underreport deaths [24], and (3) the challenges of enforcing strict lockdowns in societies that prioritize individual liberties [25].

Prevalence of specific health conditions was another big factor influencing COVID-19 pandemic outcomes. This study includes the prevalence of diabetes, obesity, hypertension, cardiovascular disease, and chronic respiratory diseases, which are comorbidities with well-known association with higher mortality in COVID-19 patients [5,20,26,27,28,29,30]. The prevalence of these conditions at the macroscopic population level provides insights into the population health vulnerability to new pandemics.

While some studies have examined the effect of specific subsets of these factors during the early stages of the pandemic [1,11,31,32,33], a comprehensive global analysis integrating all risk categories across 174 countries along the entire pandemic (years 2020 to 2023) is missing. The existing literature is largely constrained by limited geographic scope, reliance on data from only the early stages of the pandemic, or the use of broad aggregated indicators that obscure specific population dynamics. Specifically, there is a scarcity of research that systematically stratifies countries by development level; most studies either treat global data as a whole or focus exclusively on high-income nations (e.g., OECD countries), thereby overlooking how risk factors might be associated with COVID-19 pandemic outcomes differently in developing versus developed economies. Furthermore, many analyses fail to account for the complex interactions between risk factors or the temporal instability of these predictors as the virus evolved. In contrast, this study addresses these gaps by encompassing a global dataset to allow for a broader identification of population level risk factors, while employing a rigorous stratification strategy to capture context-specific associations.

To address these limitations and advance the existing literature, this study offers a distinct methodological contribution in three key dimensions. First, this study relies on aggregated national data and, thus, does not claim to find direct causality at the individual level, avoiding the risk of ecological fallacy through a rigorous stratification strategy [34] over a comprehensive dataset of 174 countries and 21 granular regressor variables. The study narrows the analytical scope, applying country aggregation according to development status, thus reducing the heterogeneity inherent in global averages. This stratified approach enables the generation of context-specific hypotheses, providing a roadmap for future research to investigate these specific subgroups in greater detail and avoid the pitfalls of broad generalizations. Specifically, the inclusion of detailed population distributions across five distinct age groups (0–4, 5–14, 15–24, 25–64, over 65)—rather than relying solely on median age or a single elderly threshold—addresses a significant gap regarding how specific demographic cohorts influence mortality at a macroscopic level. Second, the study employs a systematic data stratification approach comprising seven distinct regression experiments. By dissecting the analysis into economic country groups (developed, developing, least developed), we move beyond a static snapshot of the pandemic to capture the specific associations of relevant population level variables. This approach directly addresses the lack of comparative literature by revealing how determinants like healthcare capacity or governance shift in importance across different stages of economic development. Even where associations appear weak, these findings serve as a baseline for future researchers about which variables remain salient across different contexts. Third, the study incorporates a temporal stratification analysis by dividing the pandemic into three distinct annual periods (2020, 2021, and 2022). This longitudinal perspective allows us to assess the dynamic nature of the pandemic and determine how structural factors—such as institutional transparency or population density—gained or lost relevance over time, especially following the global vaccination rollout and the emergence of different variants. Fourth, by employing a stepwise multiple linear regression model, this study prioritizes a data-driven identification of the most dominant regressors, isolating the variables with the highest explanatory power from the noise.

Building on this framework, we investigate the relationship between the COVID-19 AMR and these 21 regressor variables, categorized into five broad groups: demographic factors (including detailed age structures), socioeconomic indicators (e.g., Gini index, HDI), economic indicators (e.g., GDP, unemployment), structural and political metrics (e.g., Democracy Index), and prevalence of health conditions (e.g., obesity, diabetes). A stepwise multiple linear regression modeling approach identifies the variables strongly associated with COVID-19 AMR.

The current study encompasses ten regression analysis experiments designed to assess the association of AMR with selected regressors in diverse settings across economic development groups and distinct time periods. The multiple analysis carried out in this paper yields critical insights that refine the current understanding of pandemic mortality. First, consistent with established literature, our main-effects analysis identified advanced age (specifically the population over 65) as the most robust and universal predictor of mortality across nearly all economic clusters. Crucially, the baseline approach also highlighted ecological paradoxes—such as the significant negative association with diabetes prevalence—underscoring the necessity of distinguishing between individual biological risks and national-level healthcare dynamics. Second, we demonstrate that secondary risk drivers are highly context-dependent. While structural factors like unemployment and life expectancy were significant in developed nations (Analysis #2), socioeconomic inequality (Gini index) played a more dominant role in developing contexts (Analyses #3 and #7). Third, our temporal assessments (Analyses #8–#10) reveal a critical longitudinal shift in pandemic dynamics: while clinical and health conditions (such as obesity and diabetes) were the primary drivers of mortality during the initial phases of the pandemic, socioeconomic indicators (including GDP and the Gini index) emerged as the dominant predictors in the later stages. By integrating this broad set of independent variables, temporal phases, and a robust analytical framework, this research seeks to uncover both universal and context-specific determinants of COVID-19 mortality, providing valuable insights for improving global preparedness for future public health emergencies.

## 2. Methods

### 2.1. Data

**Figure 1:**
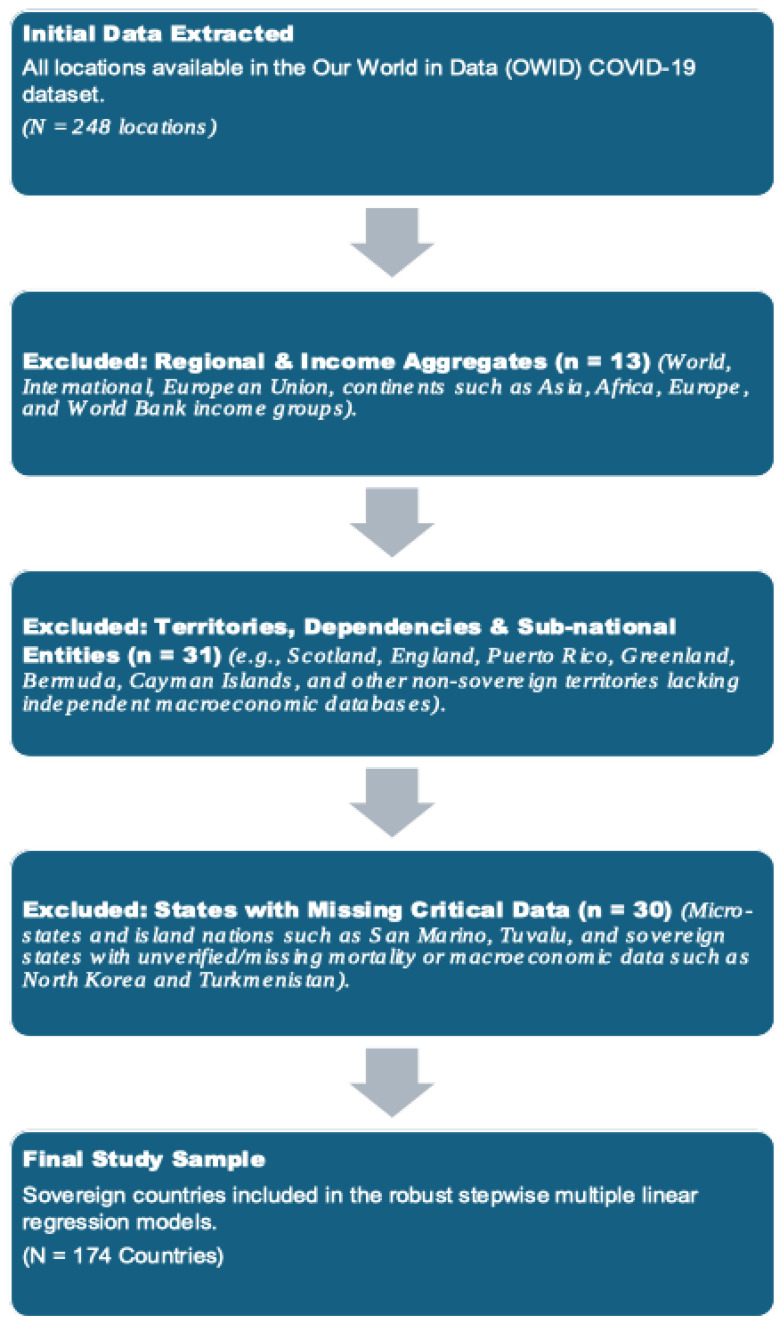
Flow diagram of country inclusion and exclusion criteria. The flowchart details the stepwise selection of the 174 sovereign countries included in the final analysis, starting from the initial 248 locations extracted from the Our World in Data (OWID) repository.

This ecological study was conducted and reported in accordance with the Strengthening the Reporting of Observational Studies in Epidemiology (STROBE) guidelines for observational studies. A detailed flowchart illustrating the stepwise inclusion and exclusion criteria is provided in Figure 1. Data and code as well as Supplementary Material will be openly accessible at zenodo repository referred below upon paper acceptance.

“Our World in Data” (OWID) is a website devoted to the publication of relevant data for pressing geopolitical and economic issues. It is hosted by the University of Oxford. OWID has been publishing relevant epidemiological data for the follow-up of the COVID-19 pandemic, aggregating it from several sources. To compute the dependent variable of the study, i.e., the COVID-19 AMR, we use the smoothed deaths relative to population as published in the OWID site, which is a seven-days moving average. The OWID site was capturing the mortality time series from the Johns Hopkins University COVID-19 site until March 2023, when it stopped updating data after the World Health Organization (WHO) declared the end of the sanitary emergency. After this date, most countries stopped reporting, and the OWID site shifted to the WHO mortality time series publication. This work is based on the OWID dataset, covering the period between 21 January 2020, and 10 January 2023, thereby capturing the most significant events of the pandemic evolution. The AMR is computed as the accumulated death *per* million for each country until the final date of data capture, representing the total number of deaths explicitly attributed to COVID-19 by national public health agencies (typically validated via clinical testing or death certificates) which can follow substantially different policies for the attribution [35]. It can be argued that excess mortality data may be more robust against reporting bias than AMR, but it is frequently unavailable or relies on gross imputations for many developing and least developed countries (LDCs) included in our study. Confirmed COVID-19 deaths allow for a direct comparison across countries of diverse socioeconomic strata (developed, developing, and least developed) without excluding nations with limited death registration systems. However, to account for differences in data collection and to validate the stability of our findings against potential reporting biases, all regression analyses were replicated using cumulative excess mortality data—specifically the central estimate of cumulative excess deaths per 100,000 people—sourced from OWID [36].

The data for the independent variables (i.e., the regressors) utilized in this study were gathered from various reputable sources, with a focus on providing the most recent available information, summarized in Table 1. As health-related indicators we consider the prevalence of obesity (body mass index (BMI) ≥ 30 in WHO nomenclature) among adults, the prevalence of diabetes (given as the percentage of population ages 20–79, reported by the standard protocols established by the International Diabetes Federation (IDF) and the World Bank), the prevalence of hypertension in adults aged 30–79, the prevalence of chronic respiratory diseases, and the prevalence of cardiovascular diseases (as reported in 2021). The demographic variables considered include the population spatial density (population per unit area) and age demographics, comprising the median age and the distribution of population into five age groups (0–4, 5–14, 15–24, 25–64, and 65+). As social indicators we consider the Gini coefficient, GDP *per* Capita, Human Development Index (HDI), Inequality-adjusted HDI (IHDI), Gender Inequality Index (GII), life expectancy, Democracy Index, and unemployment ratio.

To mitigate potential endogeneity and reverse causality—where pandemic effects might influence the regressor variables—we adopted a split temporal approach. For structural health and development indicators (life expectancy, HDI, IHDI), the study data is strictly restricted to pre-pandemic baseline values of the year 2019, as these indices may be strongly influenced by pandemic social effects. Conversely, for economic variables (GDP, unemployment), the study uses the average of years 2020 to 2022. This selection was intended to accurately reflect the real-time economic capacity and resource constraints nations faced while managing the crisis, as pre-pandemic wealth metrics would not account for the rapid economic contractions that limited emergency responses in several regions.

For a limited number of small countries suffering from missing data, alternative data sources or proxy values from demographically, economically, or geographically similar nations (“Reference Countries”) were utilized. To ensure transparency and address potential systematic bias, we quantify that this substitution strategy was strictly limited to only two variables—the Gini index and the Democracy Index—affecting a total of 10 unique countries. Specifically, for the Gini index, proxies were used for Venezuela (Colombia) and the Solomon Islands (Indonesia). For the Democracy Index, substitutions were applied to the Seychelles (Peru), Lebanon (Morocco), the Solomon Islands (Burkina Faso), the Marshall Islands (Portugal), Micronesia (Portugal), South Sudan (Sudan), Somalia (Afghanistan), Maldives (Burkina Faso), and São Tomé and Príncipe (Botswana). This careful imputation was performed only when strictly necessary to maintain dataset completeness and prevent the total exclusion of these developing nations from the macroscopic analysis. Furthermore, to address concerns regarding the potential distortion of estimated associations, we conducted a sensitivity analysis by completely excluding the countries with substituted values. The results of these sensitivity regressions confirm that the statistical signs and the overall direction of the associations remained entirely unchanged, demonstrating the robustness of our main findings. The results are provided in the Supplementary Material file “Sensitivity Analysis Regression Models Excluding Referenced Countries (N=165).docx”. Finally, a comprehensive overview of all independent variables, including these specific substitutions, original data sources, reference years, and any preprocessing applied, is provided in the Supplementary Material “Supplementary Table S1” to ensure full reproducibility.

### 2.2. Statistical Analyses

This study was designed as an observational ecological study utilizing aggregated country-level data. To investigate the macroscopic associations between the COVID-19 AMR and the selected set of demographic, health, economic, and political variables, we employed stepwise multiple multivariate linear regression [37] as implemented by the MATLAB 2025b “stepwiselm” function. This method enables automatic model building by iteratively selecting predictors based on the statistical significance of their contribution to model performance. It is a variable selection technique used in the context of multiple linear regression, where the goal is to identify the subset of predictor variables that best explain the variability of the dependent variable. The stepwise approach iteratively adds or removes variables based on predefined statistical criteria such as conditions on *p*-values, F-statistics, or information criteria (e.g., AIC or BIC), aiming to prevent overfitting and to improve model parsimony [38], helping to identify the most statistically significant variables while controlling for multicollinearity and model complexity [39]. This approach is particularly useful when analyzing high-dimensional datasets where many potential predictors exist. There are three alternative procedures for variable selection: (1) Forward selection: begins with no predictors and adds variables incrementally. (2) Backward elimination: starts with all predictors and removes non-significant ones. (3) Bidirectional (stepwise): iteratively adds and removes predictors based on statistical criteria. Specifically, this study uses the bidirectional stepwise approach for variable selection, which combines forward selection and backward elimination.

The model selection criteria that prioritize statistical robustness while keeping model complexity in check are as follows: a predictor must have p<0.05 to remain in the model, and a predictor is removed if p>0.10. The overall optimization criterion is the root mean squared error (RMSE) $R^{2}$ that the stepwise process is trying to minimize at each variable selection step. To allow for a direct comparison of predictor strength across variables with differing units and scales, we computed standardized regression coefficients (standardized beta). While the sign of the coefficient reflects the direction of the association, the absolute magnitude of the standardized beta ($\left|\beta \right|$) indicates the relative influence of each predictor.

In this study, the dependent variable (*Y*) is defined as the accumulated mortality ratio (AMR) *per* country. The set of candidate independent variables ($X_{candidate}$) consists of 21 potential predictors categorized into three main categories of variable, namely, health determinants (e.g., age, obesity prevalence, diabetes), demographic factors (e.g., 65+ population, median age), and socioeconomic indices (e.g., Gini, GDP), that were detailed in the Data section above. The final regression model is expressed by the general linear model displayed in Algorithm 1:
**Algorithm 1** Multivariate linear regression.$Y=\beta_{0}\;+\sum_{j=1}^{k}\beta_{j}X_{j}+\epsilon$where*Y* is the predicted accumulated mortality ratio;$\beta_{0}$ is the intercept (constant term);$X_{1},X_{2},\ldots ,X_{k}$ represent the specific subset of k independent variables selected from the candidate pool of variables by the stepwise algorithm;$\beta_{1},\beta_{2},\ldots ,\beta_{k}$ are the regression coefficients corresponding to the selected variables, and ϵ is the error term (residuals).

The modeling approach of the study is a main effects-only regression that limits the model to the main effects, excluding interactions. While it is simpler though less explanatory in terms of fit, this modeling approach offers a clearer view of each predictor’s direct association with COVID-19 AMR.

The following diagnostic tests were conducted to assess the validity of regression assumptions. Their detailed results are provided in the Supplementary Material in the file “Supplementary Material—AMR Regression Results and Diagnostics.docx”:

- A scatter plot of actual versus predicted values was drawn to assess the assumption of linearity.
- Standardized residuals were calculated for each observation to detect potential outliers, particularly those exceeding ±3 standard deviations.
- Partial regression plots were constructed for each predictor to visually inspect their individual contributions to the dependent variable and identify influential observations.
- Assessment of multicollinearity: First, while bidirectional stepwise selection may reduce redundancy by excluding predictors with negligible partial contributions, it does not constitute a formal diagnostic or remedy for multicollinearity. To further diagnose potential issues, variance inflation factor (VIF) values were calculated. Detailed diagnostic results, including specific VIF values and checks for exploratory interaction models, are provided in the Supplementary Material (Regression Results and Diagnostics).
- The Durbin–Watson statistic was used to test for autocorrelation of residuals.
- To rigorously assess the presence of heteroscedasticity, a plot of predicted values versus standardized residuals was examined in conjunction with formal statistical testing using the Breusch–Pagan and White tests.
- A normal probability (P–P) plot of standardized residuals was generated to evaluate the assumption of normality in the residuals.

In Analyses #1, #4, #6, described below, diagnostic plots and statistical tests (Breusch–Pagan and White) indicated the occurrence of heteroscedasticity, i.e., a violation of the assumption of constant variance of the residuals by linear regression models. To address this issue, robust model fitting was used in these analysis [40,41]. Robust regression methods, such as those employing iteratively reweighed least squares, reduce the influence of outliers and heteroscedastic errors by assigning lower weights to observations with large residuals. Robust coefficients were estimated using M-estimation with Tukey’s bisquare weight function, implemented via the iteratively reweighted least squares (IRLS) algorithm as in MATLAB’s *robustfit* function. Standard errors are asymptotic and were computed from the final weighted least squares fit using the estimated scale and IRLS weights [42].

Given the multiple regression models and numerous predictors assessed in this study, there is an increased risk of Type I errors (false positives). To address this multiplicity, we applied the Benjamini–Hochberg false discovery rate (FDR) procedure [43] to the resulting *p*-values. Adjusted *p*-values (q-values) were calculated across the analyses to rigorously validate the significance of our findings, with an FDR threshold of 5% considered statistically significant. The detailed results of FDR analysis are provided in the Supplementary Material in file “Supplementary Table S2 FDR Results.xlsx”.

### 2.3. Modeling Framework

To investigate the differential impacts and responses related to COVID-19 across varying levels of economic development, we carried out a comprehensive design consisting of seven distinct regression analyses. The objective of these analyses is to identify variations in associations of variables with the AMR across countries classified as developed, developing, and least developed. Additionally, the analysis has been also applied to data from each year of the pandemic separately, in order to assess some longitudinal changes. Table 2 summarizes the modeling framework design.

## 3. Results

Detailed computational outputs of all regression models are available in the Supplementary Material, which is also published in the zenodo repository (https://doi.org/10.5281/zenodo.18879993).

### 3.1. Global Determinants and Structural Interactions (Analysis #1)

There are statistically significant differences among the groups of countries defined according to their status of development, as can be appreciated in the results of two way non-parametric *t*-tests that are provided in the Supplementary Material File “t_test_results.xlsx”. The results of the global analysis (N=174) are summarized in Table 3, revealing that demographic structure is the primary driver of pandemic mortality. In this baseline model, the proportion of the population aged over 65 emerged as the strongest association with AMR (β=0.77, p<0.001), followed by the prevalence of comorbidities such as obesity (β = 0.25,p<0.001) and hypertension (β = 0.16, p<0.001). Interestingly, the global multivariate model showed a counterintuitive negative association between diabetes prevalence and AMR (β = −0.11, p=0.015). To verify that this result is not an ecological paradox and, thus, reflects genuine differences in healthcare capacity rather than a statistical modeling artifact, we explicitly tested the unadjusted bivariate associations across different economic (GDP) and development (HDI) strata (results provided in the Supplementary Material files “Figure S2. Univariate Regression Trends Stratified by Economic Capacity (GDP).jpg” and “Figure S3. Univariate Regression Trends Stratified by Human Development Index (HDI).jpg”). These stratified diagnostic analyses reveal a clear directional shift consistent with our proposed mechanism. In high-GDP and high-HDI nations, the association is significantly (p=0.011 and p=0.043, respectively) negative, driving the global model’s inverse relationship. Conversely, within low-GDP and low-HDI strata, the slope reverses to a positive directional trend, which aligns with established expectations of clinical resources. However, this positive trend in lower-income regions does not reach statistical significance (p>0.05), which is likely attributable to profound data noise and structural limitations. In these countries, widespread under-reporting of both diabetes prevalence and true COVID-19 mortality inflates statistical variance. Furthermore, the specific effect of diabetes is likely overshadowed by dominant competing risks—such as severe shortages in healthcare infrastructure, limited ICU capacity, and delayed hospital admissions—rendering the isolated effect of diabetes statistically undetectable.

On the side of the structural variables, the study finds a positive relation between Gini index and AMR (β = 0.17, p=0.001), and a negative association with the GDP per capita (β = −0.11, p<0.03).

**Figure 2:**
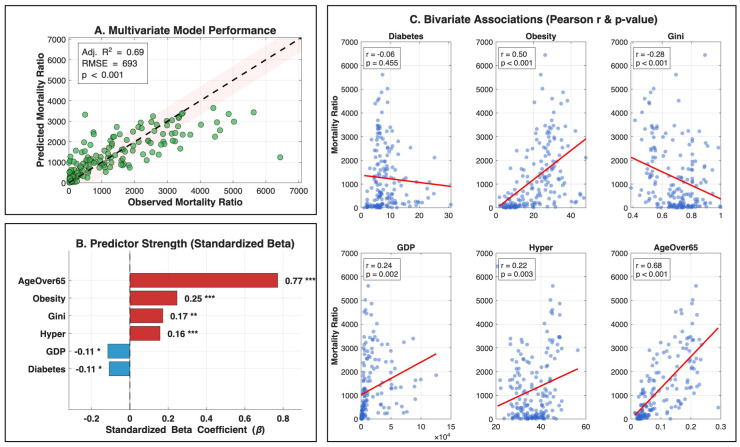
Integrated analysis of macroscopic determinants for COVID-19 accumulated mortality ratio (AMR). (**A**) Multivariate model performance comparing observed versus predicted AMR. Each point represents an individual country. The dashed black line represents perfect prediction (identity line), and the shaded area indicates the root mean squared error (RMSE) margin. (**B**) Predictor strength illustrated by standardized beta coefficients. Red bars denote positive associations, while blue bars denote negative associations. Significance levels are indicated by asterisks (*** *p* < 0.001, ** *p* < 0.01, * *p* < 0.05). (**C**) Bivariate associations between individual macroscopic predictors and AMR. In each scatter plot, each point represents a country, and the solid red line indicates the fitted linear trend. Pearson correlation coefficients (*r*) and *p*-values are provided in the insets.

Figure 2 is a comprehensive presentation of the results of the regression analysis, providing the graphical evaluation of model performance in panel A, predictor importance in panel B, and raw associations sequentially in panel C. First, Figure 2A validates the overall success of the stepwise model. The observed mortality rates cluster tightly along the diagonal identity line within the RMSE confidence band, showing that the model explains approximately 69% of AMR variance. Second, Figure 2B shows the independent contribution of each risk factor plotting the standardized β t-statistics. After controlling for confounding variables, age over 65 emerges as the strongest biological driver of mortality. Moreover, this panel uncovers significant effects that are not visible in raw data: obesity and the Gini index are confirmed as significant positive risk factors (p<0.05), while diabetes displays a significant negative adjusted association (t=−2.5).

Finally, Figure 2C illustrates the raw bivariate associations for comparison. A striking discrepancy is observed here: unlike the findings in Panel B, the unadjusted data depicts diabetes as statistically insignificant (p=0.455) and the Gini index as negatively correlated (r=−0.28). This contrast confirms that true risks are masked in raw data. The multivariate adjustment, illustrated in Figure 2B, resolves this by holding constant dominant drivers like age and GDP—effectively neutralizing their interference. By isolating the specific impact of each predictor as if other factors were fixed, the model strips away these masking effects, revealing true independent relationships that remain hidden in the unadjusted analysis.

Results of a model including interactions among variables are provided in the Supplementary Material. It demonstrated robust explanatory power (adjusted $R^{2}=0.756$), confirming that risk factors do not operate in isolation but are moderated by structural capacities. These results (see entry “Analysis 1 Model including interactions”) indicate that structural contexts significantly moderate biological drivers. For instance, strong health infrastructure modeled by life expectancy appears to mitigate the lethality of comorbidities like hypertension, while high unemployment disproportionately increased mortality risks in aging demographics (see Supplementary Material “Figure S4. Marginal Effects of Significant Interaction Terms on Predicted Mortality Rates”).

### 3.2. Divergent Risk Profiles Across Country Development Levels (Analyses 2–7)

When stratified by economic development, distinct associations with the AMR emerged, revealing a divide between the Global North and the Global South, as summarized in Table 4.

Developed countries (Analysis #2): The variance in mortality rates was primarily associated with clinical comorbidities and socioeconomic indicators. Notably, the prevalence of hypertension and chronic respiratory diseases exhibited the strongest positive correlation with mortality (β=0.48, p=0.001) and (β=0.41, p<0.001), respectively. Similarly, unemployment rates were significantly correlated with higher mortality figures (β=0.32, p=0.003). Furthermore, life expectancy (β=−0.35, p=0.01) showed significant negative association with AMR, suggesting a statistical link between this factor and mortality outcomes in advanced economies.

Developing and least developed countries (Analyses #3 & #4): In contrast, the primary drivers in lower-income nations were strictly demographic and economic. The percentage of population of age over 65 is the strongest association with AMR in both country groups (β = 0.73, p<0.001), and (β = 0.33, p<0.001). The prevalence of obesity is another shared structural risk for bad pandemic AMR outcomes (β = 0.328, p=0.001), and (β = 0.14, p<0.001). The Gini index was a significant positive predictor in developing countries (β = 0.24, p=0.005), underscoring the lethal role of social inequality in emerging economies. The least developed countries (LDCs) have no protective effect from healthcare factors observed in wealthier country groups.

Pairwise comparisons in Analyses #5–#7 are summarized in Table 5. The analyses across aggregated groups reinforced findings regarding country disparities. Notably, the positive association between the Democracy Index and mortality persisted in the developing vs. least developed countries comparison of Analysis #7, suggesting that reporting transparency remains a distinguishing factor even among lower-income groups. The Democracy Index exhibited a significant positive association with AMR (β = 0.14, p=0.013). While statistically controlling for confounders such as age (p<0.001) and obesity (p<0.001), this finding likely reflects the higher reporting transparency and institutional capacity of democratic nations within the developing world, rather than a direct causality.

In order to account for the multiplicity of the models evaluated, all initial *p*-values were subjected to Benjamini–Hochberg FDR correction. Most of the primary strong associations—namely, age over 65, obesity prevalence, diabetes, and the Gini index—retained their robust statistical significance after FDR adjustment, confirming that these are not chance findings. The comprehensive table comparing original and FDR-adjusted *p*-values is provided in the Supplementary Material “Supplementary Table S2 FDR Results.xlsx”.

### 3.3. Associations Across the Pandemic Years

To examine the dynamic nature of the pandemic, mitigate potential seasonal biases, and assess how the relevance of structural factors—such as governance (Democracy Index) or population spatial density—evolved over the years, the dataset was stratified into three distinct annual periods. As shown in Table 6, the analyses reveal a clear longitudinal shift in macroscopic mortality drivers:

- First Year (21 January 2020–20 January 2021, Analysis #8): During the first year of the pandemic, macroscopic mortality was driven almost exclusively by demographic and clinical vulnerabilities. The proportion of the population aged over 65 was the strongest predictor (β=0.45, p<0.0001), accompanied by obesity (β=0.34, p<0.0001) and diabetes (β=−0.22, p<0.001). Notably, socioeconomic, structural, and political variables did not reach statistical significance during this early period.
- Second Year (21 January 2021–20 January 2022, Analysis #9): As the pandemic progressed into its second year, a critical shift occurred. While demographic and health factors such as median age (β=0.62, p<0.0001), hypertension (β=0.23, p<0.0001), and obesity (β=0.19, p<0.001) remained highly relevant, structural and political variables entered the model. The Democracy Index emerged as a significant predictor of reported mortality (β=0.13, p<0.01), alongside a strong protective association from GDP per capita (β=−0.33, p<0.0001). This indicates that during this phase, institutional transparency, data reporting practices, and economic capacity became key differentiators in mortality outcomes.
- Third Year (21 January 2022–10 January 2023, Analysis #10): In the third year of the pandemic, socioeconomic disparity became a dominant macroscopic driver. Alongside the persistent risk of an aging population (age over 65: β=0.65, p<0.0001), the Gini index emerged as a significant predictor (β=0.14, p<0.001). This suggests that prolonged economic inequality heavily dictated long-term vulnerability.

Finally, regarding physical proximity metrics, population spatial density was iteratively tested across all three temporal models but failed to emerge as a statistically significant predictor of accumulated mortality in any phase of the pandemic, indicating that structural and biological vulnerabilities consistently outweighed geographic density. As an additional check, one-way non-parametric *t*-tests (Mann–Whitney U, right-tailed) found that AMR in year 2021 was significantly greater than in year 2020 (p<0.001). On the other hand, AMR in 2022 was not significantly greater than in years 2020 or 2021.

### 3.4. Excess Deaths

In order to confirm the robustness of the study’s primary findings, a sensitivity analysis was conducted using cumulative excess mortality as the dependent variable. The regression results are detailed in the Supplementary Material in file “Supplementary Material—Excess Mortality Regression Results and Diagnostics.docx”. The excess mortality data were sourced from the epidemiological modeling framework developed by *The Economist*, and accessed via the Our World in Data (OWID) repository. Please note some major methodological limitations of this variable as a measure of pandemic impact. First, excess deaths are always the result of a modeling effort that tries to predict the expected deaths under normal circumstances. Such models may change from one institution to another [44], among research teams, and even depending on the subject of study [45]. Second, empirical excess mortality estimations based on actual registry data are only available for 97 of the 174 countries included in our study. For the remaining 77 countries in our dataset—constituting nearly 45% of our analysis and encompassing almost all least developed countries (e.g., Sub-Saharan nations) as well as major developing countries (e.g., India, China, Indonesia, Turkey)—the baseline death counts used to compute excess mortality figures are not empirical data but imputed estimates. We think that relying on a variable for which a substantial portion of the data samples consists of modeled estimates seriously compromises the study’s empirical foundation. For this reason, we utilized the reported accumulated mortality ratio (AMR) as the primary macroscopic dependent variable, leveraging excess mortality strictly as a supplementary robustness check.

## 4. Discussion

In all the analyses carried out in this study, the prevalence of respiratory diseases was not identified as a primary association with AMR in the multivariate models, with the exception of Analysis #2 (developed countries). While this might appear contrary to the clinical definition of COVID-19 as a respiratory disease [46], this finding does not necessarily imply a lack of biological relevance. Rather, it suggests that at the macroscopic level, the explanatory power of the prevalence of respiratory disease is overshadowed by more dominant demographic and systemic drivers, such as population aging and prevalence of obesity, which captured the majority of the variance in our stepwise regression framework.

Demographic structure remains the consistent statistical predictor across all analyses, with the proportion of the population aged over 65 consistently serving as the primary predictor of mortality across all socioeconomic strata and pandemic phases, consistent with the prior literature [3,4,5,6,7]. While specific nuances emerged—such as the negative association observed in the age group 25–64 in least developed countries—younger cohorts failed to demonstrate consistent explanatory power. For instance, the age 5–14 group in Analysis #4 yielded a statistically not-significant coefficient (*p* = 0.301). These findings justify prioritizing age over 65 as the most salient demographic association with AMR, treating other age stratifications as necessary controls.

Regarding prevalence of health conditions, although studies at the early stages of the pandemic did not find a close relation between hypertension and increased COVID-19 mortality, some authors suggested a protective effect of anti-hypertensive drugs [28], and some meta-analyses report an independent association with increased risk of in-hospital COVID-19 mortality [47]. This study identified a significant positive association between hypertension and mortality in the global model (Analysis #1), and developed nations (Analysis #2). Diabetes has been strongly associated with worse outcomes of COVID-19 diagnosis, up to double the risk of death [48], with the diagnostic of diabetes in COVID-19 being associated with up to 16% of COVID-19 attributed deaths [49]. The disruption of the treatments due to pandemic measures may be related to this increased morbidity and mortality [30,50]. There seems to be a two-way interaction between COVID-19 and diabetes [51], with the occurrence of new diabetes onset after suffering COVID-19 disease [52]. Therefore, we should expect a strong positive association between diabetes prevalence and COVID-19 AMR. However, the univariate trends in Figures S2 and S3 reveal a divergence regarding prevalence of diabetes: while it exhibits a positive directional trend—though not statistically significant—with AMR in lower-income and less-developed settings, it shows a significant negative association in high-income and highly developed nations. This contrast underscores the risk of ecological fallacy and highlights the relevance of healthcare capacity in managing chronic conditions. These results invite further detailed research on the relationship between diabetes, COVID-19 prevalence, and pandemic mitigation measures. Since the early stages of the COVID-19 pandemic, a strong relation between obesity and COVID-19 mortality has been highlighted [53,54,55]. Consequently, in our study, obesity prevalence is consistently found as a significant positive association with COVID-19 AMR across all analyses, supporting earlier epidemiological findings [27]. Across categories of country development, the specific analysis for the developed countries does not find obesity as a significant AMR predictor, signaling the potential role of better healthcare systems.

Regarding socioeconomic factors, the Gini index, as a proxy for income inequality, was identified in this study as a robust predictor of COVID-19 mortality, particularly in developing countries. This is in agreement with the synergetic interplay of inequalities in the impact of the COVID-19 pandemic [1,2,56], so that the pandemic disproportionately impacted populations already disadvantaged by social and economic deprivation, which can also be appreciated inside countries like Spain [57] and Brazil [58]. Unlike prior studies that report mixed or region-specific effects of the development indices (HDI or IHDI) [17], the results in this study demonstrate that inequality—not just development level—is strongly linked to macroscopic vulnerability to COVID-19 AMR, especially when entering the model through interaction terms. Economic variables like unemployment and GDP per capita also featured prominently but exhibited nuanced behavior.

In Analysis #2, unemployment exhibits a direct and robust positive association with AMR (β = 0.33, *p* = 0.003). This pattern aligns with prior research in high-income settings [13], suggesting that socioeconomic vulnerability—manifested as job loss—is particularly lethal for the elderly, likely due to disruptions in healthcare access or financial instability. Furthermore, contrary to the protective role of urban density suggested in some studies [9], our analysis of developed nations identified clinical factors—specifically the prevalence of lung diseases and hypertension—as the primary determinants of mortality, indicating that specific disease burdens outweighed the influence of urban structure.

If we consider the countries according to their development stage, COVID-19 AMR in developed countries (in Analysis #2) shows strong associations with socioeconomic and clinical factors; namely, unemployment is a positive factor, while life expectancy is negatively associated with mortality. The prevalence of lung diseases emerged as a dominant positive predictor in this group. In developing countries (in Analysis #3), the inequality index (Gini), and the prevalence of obesity and diabetes become highly influential alongside the percentage of older people. However, in least developed countries (in Analysis #4), diabetes prevalence is not relevant, while aging factors and obesity prevalence are significantly relevant. The aggregation of developed and developing countries in Analysis #5 seems to be influenced by the complexity of the combined data, where diabetes (negative association) and obesity (positive association) emerge as significant factors alongside Gini inequality index. A similar effect is observed in Analysis #7, where the prevalence of medical conditions becomes more relevant due to their effect in the lower-ranked countries. The counterintuitive positive association between the Democracy Index and AMR observed in Analysis #7 (for developing vs. least developed countries) warrants a nuanced interpretation. This paradox likely reflects a confluence of factors suggested in the literature: the aging demographic structures are often correlated with democratic development, superior data transparency compared to potential underreporting in authoritarian regimes, and the challenges of enforcing strict lockdowns in societies prioritizing individual liberties. However, given the complexity of these institutional dynamics in lower-income settings, further research is crucial to definitively disentangle these causal pathways.

The incorporation of temporal analyses (Analyses #8–#10) provides critical context to these findings, revealing how macroscopic associations evolved across different phases of the pandemic. During the first year, AMR was almost exclusively associated with demographic and clinical vulnerabilities (aging, obesity, and diabetes). This aligns with the epidemiological hypothesis of a novel virus spreading through an immunologically naive population. However, the shift in the second year—where the Democracy Index and GDP per capita emerged as significant AMR predictors—coincides with the global vaccination rollout. This suggests that during the middle phase of the pandemic, a country’s economic capacity to procure vaccines, treatments, and its institutional framework became decisive. The significance of the Democracy Index during this period likely captures a dual effect: higher data transparency in democratic nations leading to more accurate mortality reporting, coupled with the political complexities of enforcing vaccine mandates and non-pharmaceutical interventions in societies prioritizing individual liberties. By the third year, the emergence of the Gini index as a dominant predictor highlights the compounding effect of prolonged pandemic fatigue and economic disruption, demonstrating that systemic inequality ultimately became the primary bottleneck in protecting vulnerable populations. Furthermore, the persistent insignificance of population spatial density across all three years challenges early assumptions about urban crowding, suggesting that structural inequalities, clinical baseline health, and institutional capacity were far more lethal factors than physical proximity alone.

The primary limitation of this study lies in its ecological design, implying that the observed macroscopic associations reflect country macroscopic structural vulnerabilities rather than individual biological risks and must be interpreted with caution in order not to fall in ecological fallacy when interpreting the results. While our formal FDR adjustments confirmed the robust statistical significance of the core determinants (such as aging and obesity), the nuanced, context-specific associations identified in these stratifications serve primarily as hypothesis generators for future research. Furthermore, to maximize global inclusivity (174 countries) and avoid restricting the analysis solely to developed nations where excess mortality data is available, we relied on reported COVID-19 mortality; a strategic choice that accepts potential reporting biases—particularly in less transparent regimes—in exchange for a comprehensive assessment of cross-country disparities including developing regions. Regarding data structure, while structural baseline values (e.g., life expectancy or HDI) were sourced from the pre-pandemic year 2019 to minimize the possibility of induced reverse causality, dynamic economic variables were averaged over 2020–2022 intending to capture the genuine socioeconomic shock of the crisis. Missing values were estimated based on country structural variables similarity. Finally, unmeasured confounders such as genetic heterogeneity or population compliance with non-pharmaceutical interventions could not be fully integrated into this macroscopic model because there is no published data, or the scarce studies published, for instance, regarding mask wearing [59,60], are low-quality observational studies.

## 5. Conclusions

In conclusion, this study provides a comprehensive macroscopic analysis of COVID-19 mortality drivers across 174 countries, revealing that pandemic outcomes are associated with a complex interplay of structural, demographic, and socioeconomic conditions that dynamically evolved over time. The study findings identify the proportion of the population aged over 65 as the dominant and consistent association with increased AMR, irrespective of country development stage and pandemic year. We think that dismissing the relevance of aging population, taking it as a control regressor, is like ignoring the elephant in the porcelain shop: aging population remains a strong risk factor for future pandemics. Moreover, our temporal analyses demonstrate a longitudinal shift in risk factors: while clinical vulnerabilities like obesity primarily drove mortality during the initial pre-vaccination phase, institutional and socioeconomic indicators—specifically governance (Democracy Index), GDP per capita, and income inequality (Gini index)—emerged as decisive predictors in the later stages of the pandemic. These findings underscore the necessity of adaptive, phase-responsive public health strategies that move beyond “one-size-fits-all” models, prioritizing the protection of the elderly and the mitigation of structural inequalities to build resilience against future global health crises. 

## Figures and Tables

**Table 1:** Regressor variables specifying the source of the data and the available years. WB = World Bank data bank, VHD = Vizhub Health Data, EIU = Economic Intelligence Unit, UN = United Nations.

Regressor	Data Source	Years
Obesity	WHO	2020–2022
Diabetes	WB	2021
Hypertension	OWID	2019
Chronic respiratory diseases	OWID, VHD	2020, 2021
Cardiovascular diseases	OWID	2021
Population spatial density	OWID	2020–2022
Gini	OWID, WB	2020–2022
GDP *per* capita	OWID, WB	2020–2022
Human Development Index (HDI)	OWID, WB	2019
Inequality-adjusted HDI (IHDI)	OWID, WB	2019
Gender Inequality Index (GII)	OWID, WB	2020–2022
Life expectancy	OWID, WB	2019
Democracy Index (DI)	OWID, EIU	2020–2022
Unemployment	Eurostat	2020–2022
Population age distribution	UN	2020–2022

**Table 2:** Summary of regression analyses carried out in this study.

Analysis ID	Target Scope	Period/Phase	Sample Size (N)
Analysis #1	All Countries (Global)	Full Pandemic	174
Analysis #2	Developed Countries	Full Pandemic	41
Analysis #3	Developing Countries	Full Pandemic	78
Analysis #4	Least Developed Countries	Full Pandemic	56
Analysis #5	Developed and Developing Countries	Full Pandemic	119
Analysis #6	Developed and Least Developed Countries	Full Pandemic	97
Analysis #7	Developing and Least Developed Countries	Full Pandemic	134
Analysis #8	All Countries (Global)	21 January 2020 20 January 2021	174
Analysis #9	All Countries (Global)	21 January 2021 20 January 2022	174
Analysis #10	All Countries (Global)	21 January 2022 20 January 2023	174

**Table 3:** Summary results of Analysis #1.

Analysis #	Predictor	Standardized Beta (β)	*p*-Value
Analysis #1 *n* = 174 adjusted R^2^ = 0.691 RMSE = 693 (F = 65.5 *p* < 0.001)	Diabetes	β = −0.11,	*p* = 0.015
	Obesity	β = 0.25	*p* < 0.001
	Gini	β= 0.17	*p* = 0.001
	GDP *per* Capita	β = −0.11	*p* = 0.03
	Hypertension	β = 0.16	*p* < 0.001
	Age Over 65	β = 0.77	*p* < 0.001

**Table 4:** Summary of results of combination of pairwise country categories: developed, developing, and least developed countries. CRD = chronic respiratory diseases.

Analysis #	Predictor	Standardized Beta (β)	*p*-Value
Analysis #2 *n* = 41 adjusted R^2^ = 0.55	Hypertension	β = 0.48	*p* = 0.001
	Life expectancy	β = −0.35	*p* = 0.01
	Unemployment	β = 0.32	*p* = 0.003
	CRD	β = 0.41	*p* < 0.001
Analysis #3 *n* = 77 adjusted R^2^ = 0.547	Gini	β = 0.24	*p* = 0.005
	Diabetes	β = −0.22	*p* = 0.01
	Obesity	β = 0.328	*p* = 0.001
	Age Over 65	β = 0.73	*p* < 0.001
Analysis #4 *n* = 56 adjusted R^2^ = 0.652	Obesity	β = 0.14	*p* < 0.001
	Age Over 65	β = 0.33	*p* < 0.001
	Age 5 to 14	β = 0.06	*p* = 0.301

**Table 5:** Summary of results of combination of pairwise grouping of country categories: developed + developing, developed + least developed, and developing + least developed.

Analysis #	Predictor	Standardized Beta (β)	*p*-Value
Analysis #5 *n* = 118 Adjusted R^2^ = 0.488	Diabetes	β = −0.26	*p* < 0.001
	Obesity	β = 0.37	*p* < 0.0001
	Gini	β = 0.21	*p* = 0.029
	GDP *per* Capita	β = −0.25	*p* = 0.0014
	Age Over 65	β = 0.73	*p* < 0.0001
Analysis #6 *n* = 97 Adjusted R^2^ = 0.818	Obesity	β = 0.13	*p* < 0.001
	Hypertension	β < 0.0001	*p* = 0.945
	Age Over 65	β = 0.45	*p* < 0.0001
Analysis #7 *n* = 133 Adjusted R^2^ = 0.68	Diabetes	β = −0.18	*p* = 0.002
	Obesity	β = 0.29	*p* < 0.0001
	Gini	β = 0.14	*p* = 0.011
	DI	β = 0.14	*p* = 0.013
	Age Over 65	β = 0.66	*p* < 0.0001

**Table 6:** Summary of results for each of the pandemic years over all country categories.

Analysis #	Predictor	Standardized Beta (β)	*p*-Value
Analysis #8 *n* = 174 Adjusted R^2^ = 0.405	Diabetes	β = −0.22	*p* < 0.001
	Obesity	β = 0.34	*p* < 0.0001
	Age Over 65	β = 0.45	*p* < 0.0001
Analysis #9 *n* = 174 Adjusted R^2^ = 0.596	Obesity	β = 0.19	*p* < 0.001
	DI	β = 0.13	*p* < 0.01
	GDP per Capita	β = −0.33	*p* < 0.0001
	GII	β = 0.13	*p* = 0.09
	Hypertension	β = 0.23	*p* < 0.0001
	Median Age	β = 0.62	*p* < 0.0001
Analysis #10 *n* = 174 Adjusted R^2^ = 0.552	Gini	β = 0.4	*p* = 0.23
	GDP per Capita	β = 0.14	*p* < 0.001
	Age Over 65	β = 0.65	*p* < 0.0001

## Data Availability

Data and code will be published in zenodo.org after the paper is accepted along with the Supplementary Material already published in https://doi.org/10.5281/zenodo.18879993.

## Supplementary Materials

The following supporting information can be downloaded at: https://www.mdpi.com/article/10.3390/epidemiologia7020050/s1.
