# Supplementary material for: An Ecological Study on the Mortality Impact of the COVID-19 Pandemic According to Country Development Status and Pandemic Years
Source: Epidemiologia (Basel). 2026 Apr 6;7(2):50. doi: 10.3390/epidemiologia7020050 (PMC13115391; doi:10.3390/epidemiologia7020050)
Supplement: Supplementary file 1 [file epidemiologia-07-00050-s001.zip › Figure S1 STROBE Flowchart.pdf]

Initial Data Extracted

All locations available in the Our World in Data (OWID) COVID-19 dataset.

(N = 244 locations)

Excluded: Regional & Income Aggregates (n = 14) *(World, International, European Union, continents such as Asia, Africa, Europe, and World Bank income groups).*

Excluded: Territories, Dependencies & Sub-national Entities (n = 31) *(e.g., Scotland, England, Puerto Rico, Greenland, Bermuda, Cayman Islands, and other non-sovereign territories lacking independent macroeconomic databases).*

Excluded: States with Missing Critical Data (n = 30) *(Micro-states and island nations such as San Marino, Tuvalu, and sovereign states with unverified/missing mortality or macroeconomic data such as North Korea and Turkmenistan).*

Final Study Sample

Sovereign countries included in the robust stepwise multiple linear regression models.

(N = 174 Countries)

Figure 4. Flow diagram of country inclusion and exclusion criteria. The flowchart details the stepwise selection of the 174 sovereign countries included in the final analysis, starting from the initial 248 locations extracted from the Our World in Data (OWID) repository.
