# Supplementary material for: An Ecological Study on the Mortality Impact of the COVID-19 Pandemic According to Country Development Status and Pandemic Years
Source: Epidemiologia (Basel). 2026 Apr 6;7(2):50. doi: 10.3390/epidemiologia7020050 (PMC13115391; doi:10.3390/epidemiologia7020050)
Supplement: Supplementary file 1 [file epidemiologia-07-00050-s001.zip › Supplementary material - Excess Mortality Regression Results and Diagnostics.pdf]

Excess Mortality Results

Tabla de contenido

Excess Mortality Results 1
Analysis 1 2
Analysis 1 Model including interactions 5
Analysis 2 10
Analysis 3 13
Analysis 4 16
Analysis 5 19
Analysis 6 22
Analysis 7 25
Analysis 8 28
Analysis 9 31
Analysis 10 34

Analysis 1

```
Linear regression model (robust fit):
    DeathRate ~ 1 + Obesity + Democracy + GDP + Hyper + AgeOver65
```

Estimated Coefficients:

	Estimate	SE	tStat	pValue
**(Intercept)**	-21.011	60.72	-0.34604	0.72975
**Obesity**	2.3191	0.95655	2.4244	0.016394
**Democracy**	-15.895	5.6477	-2.8144	0.005471
**GDP**	-0.0030485	0.00054925	-5.5502	1.0931e-07
**Hyper**	4.6493	1.5809	2.9409	0.0037346
**AgeOver65**	2075	192.3	10.79	5.8941e-21

Number of observations: 174, Error degrees of freedom: 168

```
Root Mean Squared Error: 122
R-squared: 0.522,  Adjusted R-Squared: 0.508
F-statistic vs. constant model: 36.7, p-value = 2.76e-25
```

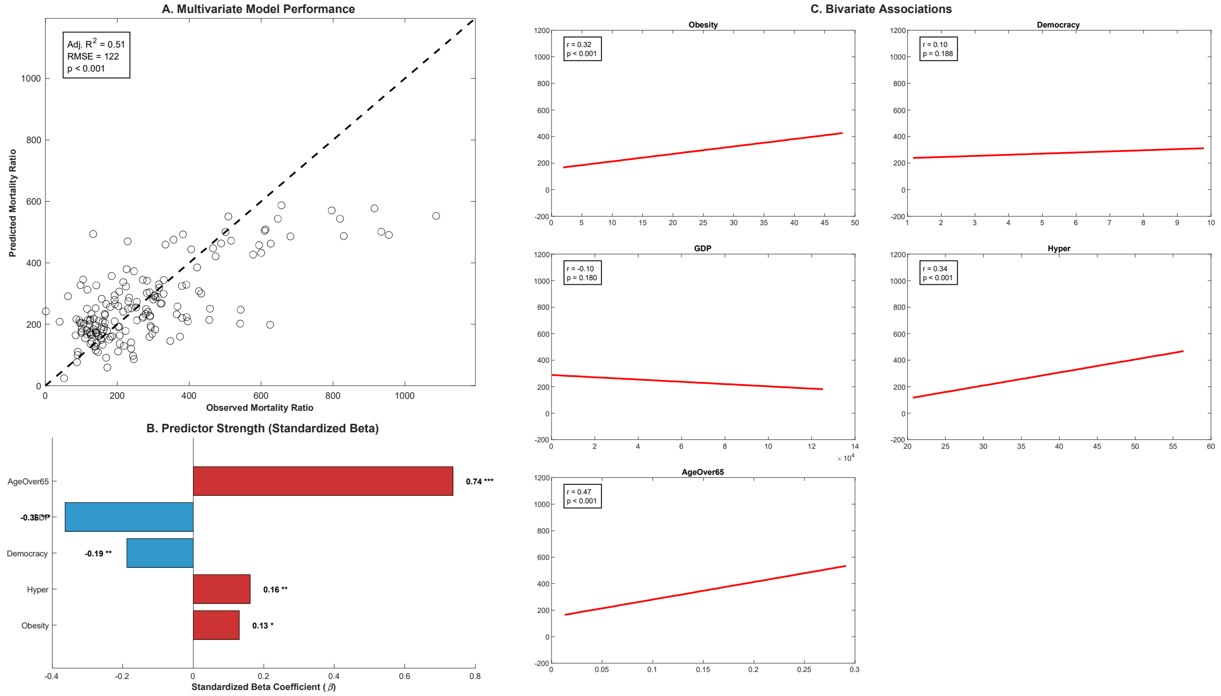

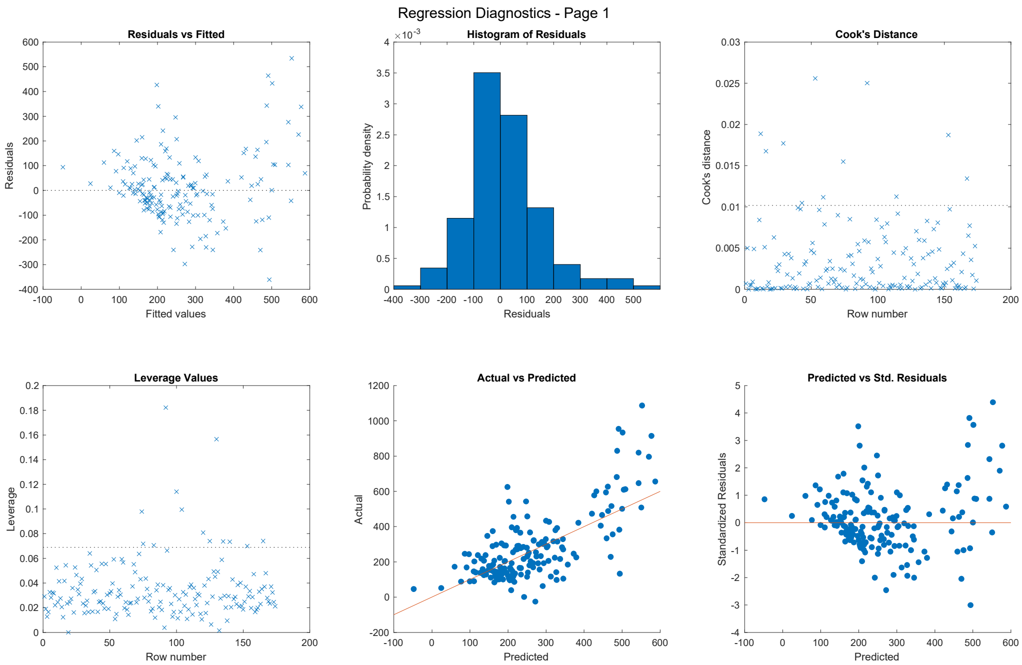
Regression Diagnostics - Page 1
Residuals vs Fitted
Residuals
Fitted values
Histogram of Residuals
Probability density
Residuals
Cook's Distance
Cook's distance
Row number
Leverage Values
Leverage
Row number
Actual vs Predicted
Actual
Predicted
Predicted vs Std. Residuals
Standardized Residuals
Predicted

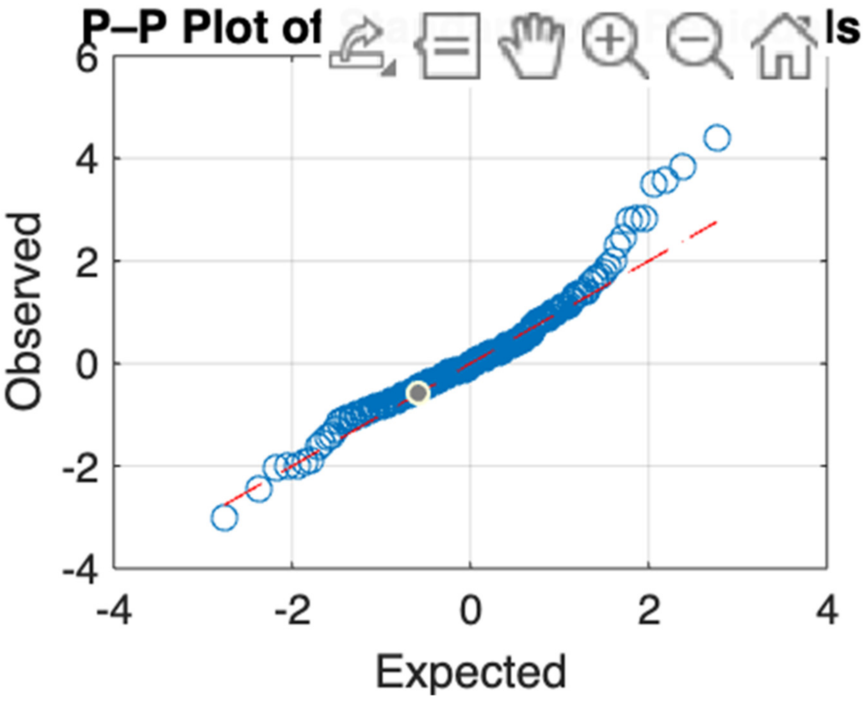
P–P Plot of
Observed
Expected

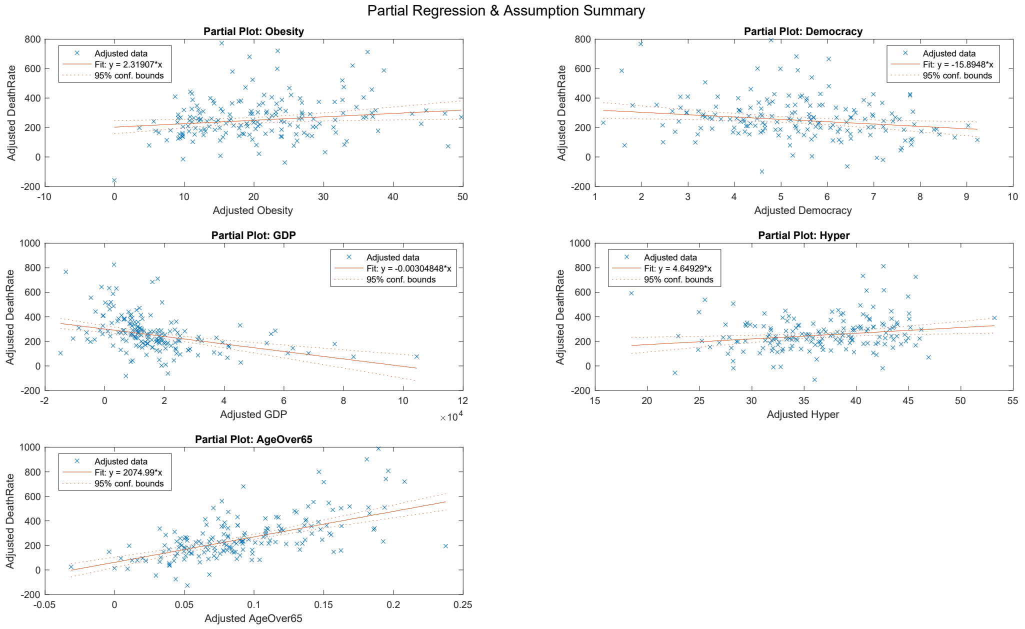

Model Assumption Tests Summary

Durbin-Watson Statistic: 1.843

Breusch–Pagan LM Stat: 26.938 | p-value: 0.0001 → Violated

White Test Stat: 55.534 | p-value: 0.0000 → Violated

Variance Inflation Factor (VIF) Values:

Predictor	VIF
Obesity	1.30
Democracy	2.01
GDP	1.92
Hyper	1.36
AgeOver65	2.09

Analysis 1 Model including interactions

```
Linear regression model:
    DeathRate ~ 1 + Diabets*Democracy + Diabets*IHDI + Diabets*GII +
Diabets*Hyper + Obesity*Democracy + Obesity*LifeExpectancy +
Democracy*Density + Democracy*GDP + Democracy*GII + Density*GII +
Density*Hyper + Density*AgeOver65 + GDP*IHDI + GDP*GII + IHDI*LifeExpectancy
+ GII*AgeOver65 + Hyper*LifeExpectancy + Hyper*AgeOver65 + Hyper*Unemployment
+ AgeOver65*Unemployment
```

Estimated Coefficients:

	Estimate	SE	tStat	pValue
**(Intercept)**	316.21	890.24	0.3552	0.72297
**Diabets**	-95.529	16.183	-5.903	2.5091e-08
**Obesity**	-20.627	11.247	-1.834	0.068751
**Democracy**	-40.225	15.398	-2.6124	0.0099585
**Density**	1.1828	0.26935	4.3914	2.1866e-05
**GDP**	0.011064	0.0034172	3.2378	0.0014996
**IHDI**	-43.065	11.007	-3.9126	0.00014111
**GII**	-3.0127	0.86765	-3.4723	0.00068421
**Hyper**	56.852	22.298	2.5497	0.011844
**LifeExpectancy**	12.546	13.385	0.9373	0.3502
**AgeOver65**	-3570.7	1257.3	-2.8399	0.0051755
**Unemployment**	-35.268	9.3493	-3.7722	0.00023681
**Diabets:Democracy**	5.2174	1.1768	4.4336	1.8418e-05
**Diabets:IHDI**	0.48562	0.22416	2.1664	0.031952
**Diabets:GII**	0.19804	0.073307	2.7016	0.0077423
**Diabets:Hyper**	1.2802	0.33418	3.8309	0.00019105
**Obesity:Democracy**	-1.6718	0.44138	-3.7877	0.0002238
**Obesity:LifeExpectancy**	0.47687	0.16181	2.9471	0.0037504

**Democracy:Density**	0.030654	0.0084993	3.6067	0.00042866
**Democracy:GDP**	-0.00096548	0.00031015	-3.1129	0.0022408
**Democracy:GII**	0.29738	0.15601	1.9062	0.058642
**Density:GII**	-0.0035577	0.001151	-3.091	0.0024011
**Density:Hyper**	-0.031993	0.007272	-4.3994	2.1164e-05
**Density:AgeOver65**	-2.9944	0.78157	-3.8313	0.00019079
**GDP:IHDI**	-0.00029498	0.00015606	-1.8902	0.060771
**GDP:GII**	-0.00013622	7.0694e-05	-1.927	0.055982
**IHDI:LifeExpectancy**	0.6123	0.15592	3.9269	0.00013372
**GII:AgeOver65**	28.378	10.402	2.7282	0.0071731
**Hyper:LifeExpectancy**	-1.1585	0.33258	-3.4833	0.00065875
**Hyper:AgeOver65**	165.94	29.833	5.5623	1.282e-07
**Hyper:Unemployment**	0.79728	0.23651	3.371	0.00096502
**AgeOver65:Unemployment**	66.74	28.462	2.3449	0.020416

```
Number of observations: 174, Error degrees of freedom: 142
Root Mean Squared Error: 91.6
R-squared: 0.82,  Adjusted R-Squared: 0.781
F-statistic vs. constant model: 20.9, p-value = 6.39e-39
```

- In Modeling Approach B (Interaction-Inclusive), specific granular age cohorts (0–4, 5–14, 15–24, 25–64), HDI and the prevalence of Lung Diseases were manually excluded from the candidate pool to prevent a rank-deficient design matrix (which yielded NaN statistics in preliminary runs).

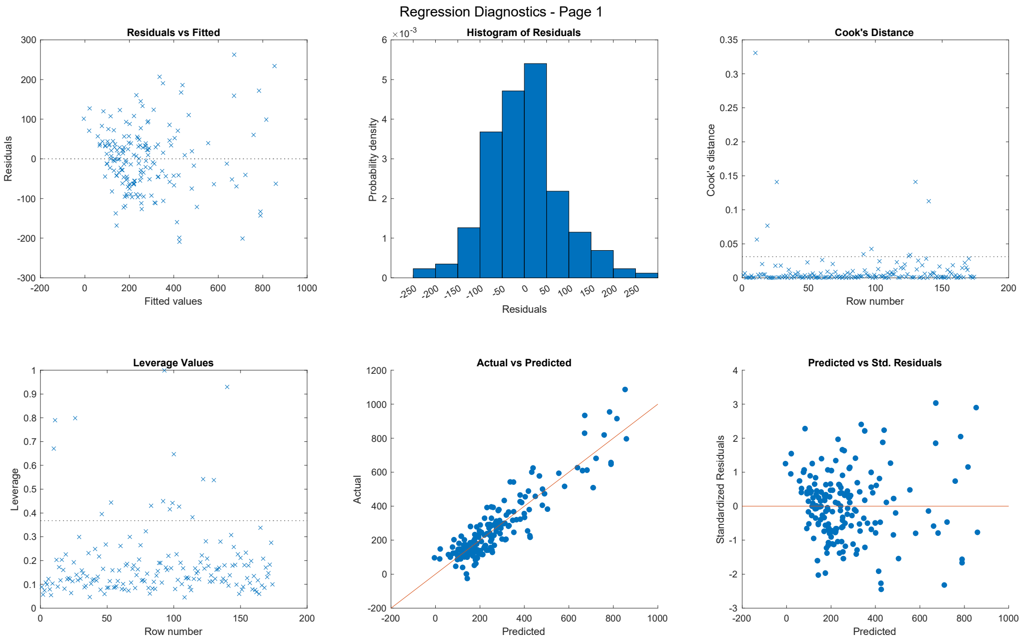
Regression Diagnostics - Page 1
Residuals vs Fitted
Histogram of Residuals
Cook's Distance
Leverage Values
Actual vs Predicted
Predicted vs Std. Residuals
Residuals
Fitted values
Probability density
Cook's distance
Row number
Leverage
Actual
Predicted
Standardized Residuals

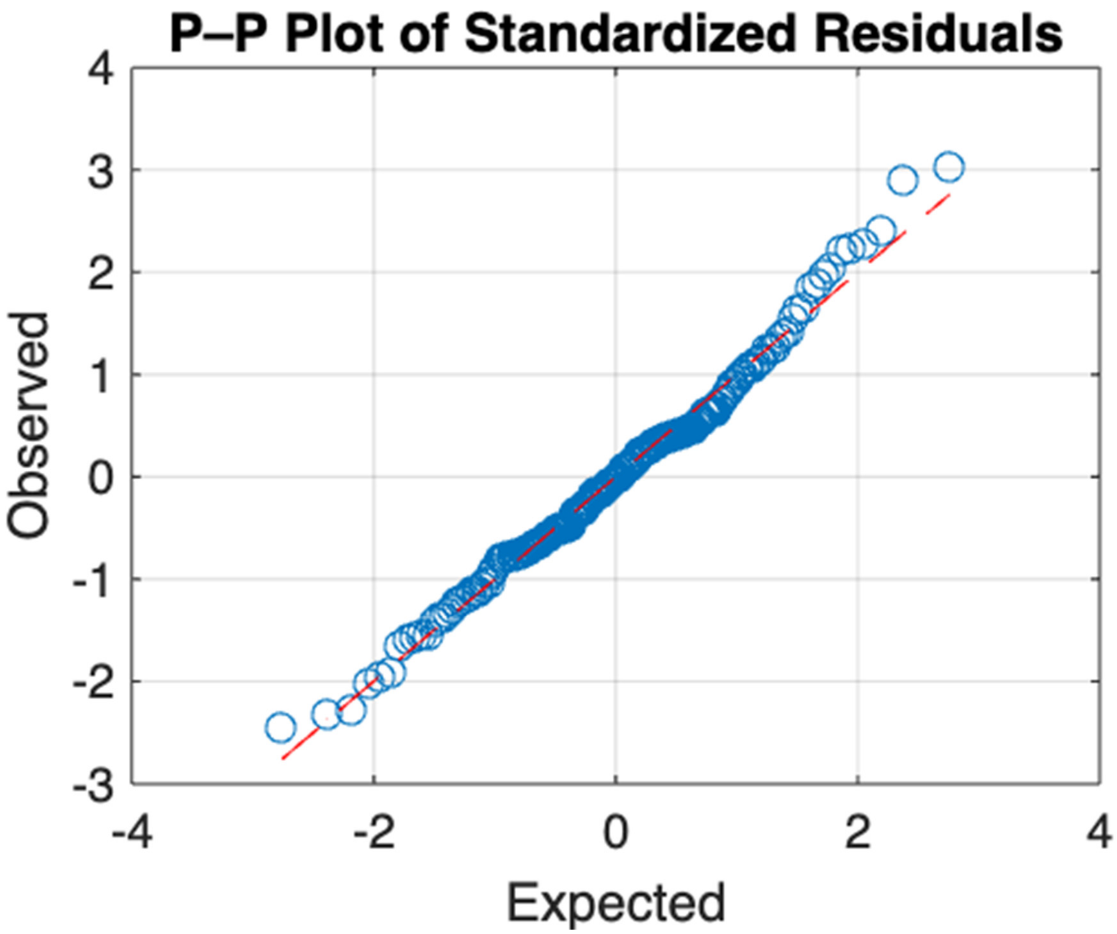
P–P Plot of Standardized Residuals
Observed
Expected

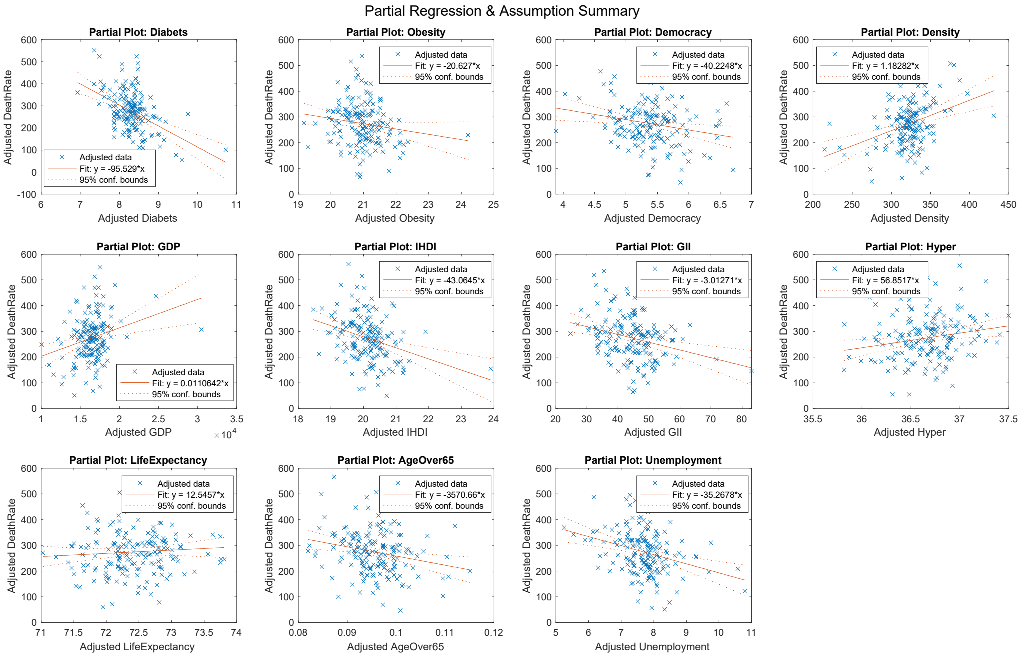
Partial Regression & Assumption Summary
Partial Plot: Diabets
Adjusted data
Fit: y = -95.529*x
95% conf. bounds
Adjusted Diabets
Adjusted DeathRate
Partial Plot: Obesity
Fit: y = -20.627*x
Adjusted Obesity
Partial Plot: Democracy
Fit: y = -40.2248*x
Adjusted Democracy
Partial Plot: Density
Fit: y = 1.18282*x
Adjusted Density
Partial Plot: GDP
Fit: y = 0.0110642*x
Adjusted GDP
Partial Plot: IHDI
Fit: y = -43.0645*x
Adjusted IHDI
Partial Plot: GII
Fit: y = -3.01271*x
Adjusted GII
Partial Plot: Hyper
Fit: y = 56.8517*x
Adjusted Hyper
Partial Plot: LifeExpectancy
Fit: y = 12.5457*x
Adjusted LifeExpectancy
Partial Plot: AgeOver65
Fit: y = -3570.66*x
Adjusted AgeOver65
Partial Plot: Unemployment
Fit: y = -35.2678*x
Adjusted Unemployment

Model Assumption Tests Summary

Durbin-Watson Statistic: 1.896

Breusch–Pagan LM Stat: 28.203 | p-value: 0.0030 → Violated

White Test Stat: 82.359 | p-value: 0.3172 → Satisfied

Variance Inflation Factor (VIF) Values:

Predictor	VIF
Diabets	1.45
Obesity	2.32
Democracy	2.13
Density	1.13
GDP	2.26
IHDI	5.86
GII	4.13
Hyper	1.54
LifeExpectancy	5.97
AgeOver65	3.42
Unemployment	1.26

Analysis 2

Linear regression model:
    DeathRate ~ 1 + Obesity + Hyper + LifeExpectancy + MedianAge

Estimated Coefficients:

	Estimate	SE	tStat	pValue
**(Intercept)**	2100.2	862.05	2.4363	0.019917
**Obesity**	3.8721	1.7324	2.2352	0.031701
**Hyper**	8.9275	3.4115	2.6169	0.012891
**LifeExpectancy**	-35.882	9.8083	-3.6584	0.00080624
**MedianAge**	17.618	4.6396	3.7973	0.00054223

Number of observations: 41, Error degrees of freedom: 36
Root Mean Squared Error: 99.8
R-squared: 0.736, Adjusted R-Squared: 0.706
F-statistic vs. constant model: 25, p-value = 5.65e-10

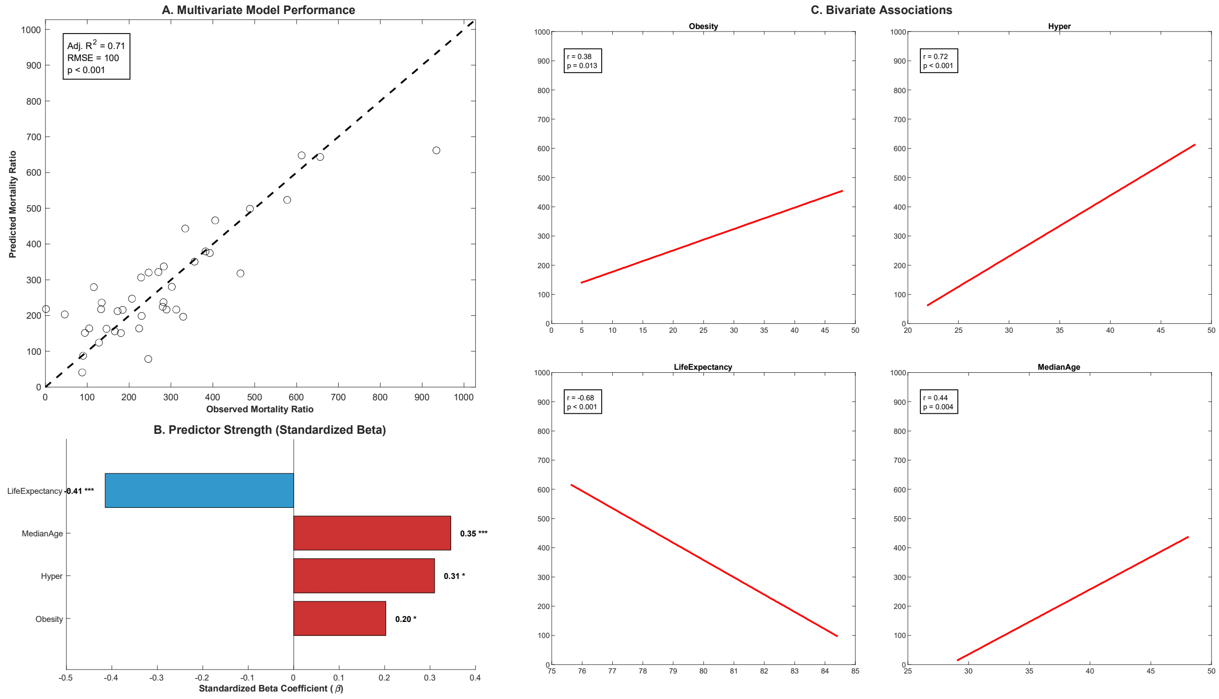

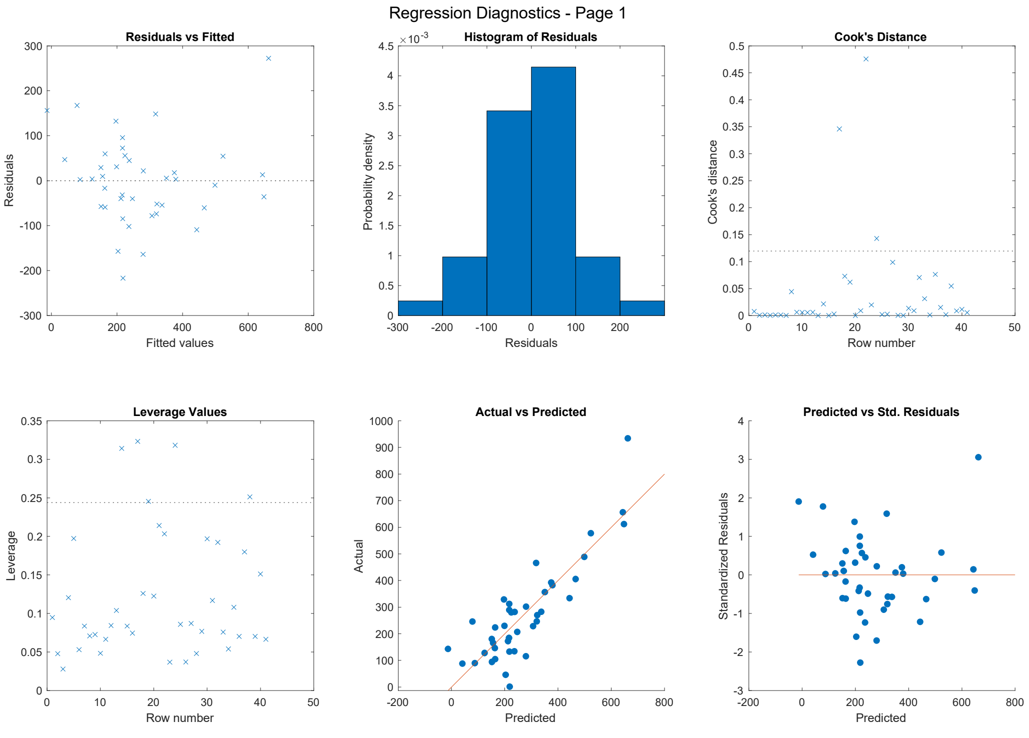
Regression Diagnostics - Page 1
Residuals vs Fitted
Residuals
Fitted values
Histogram of Residuals
Probability density
Residuals
Cook's Distance
Cook's distance
Row number
Leverage Values
Leverage
Row number
Actual vs Predicted
Actual
Predicted
Predicted vs Std. Residuals
Standardized Residuals
Predicted

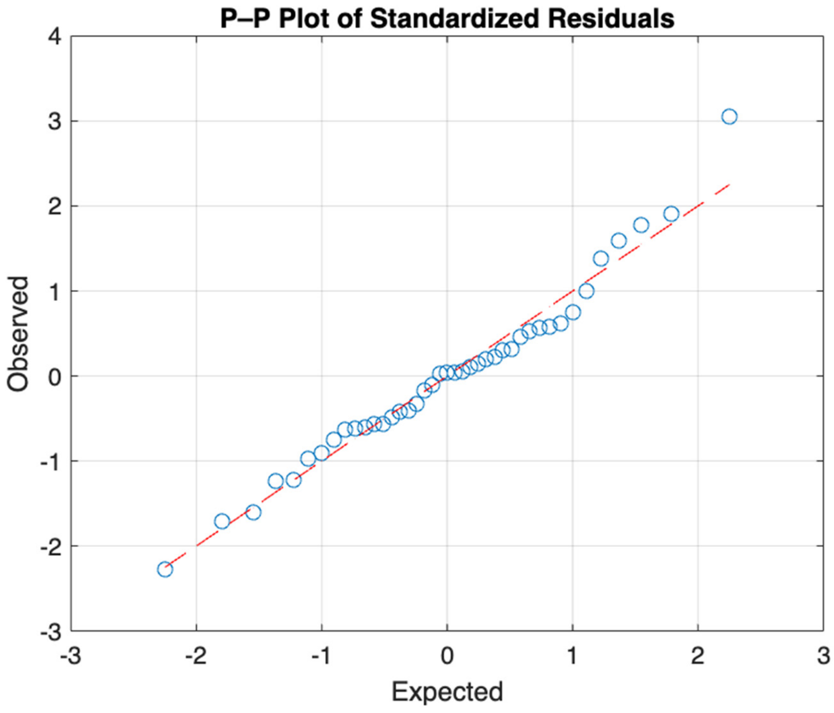
P–P Plot of Standardized Residuals
Observed
Expected

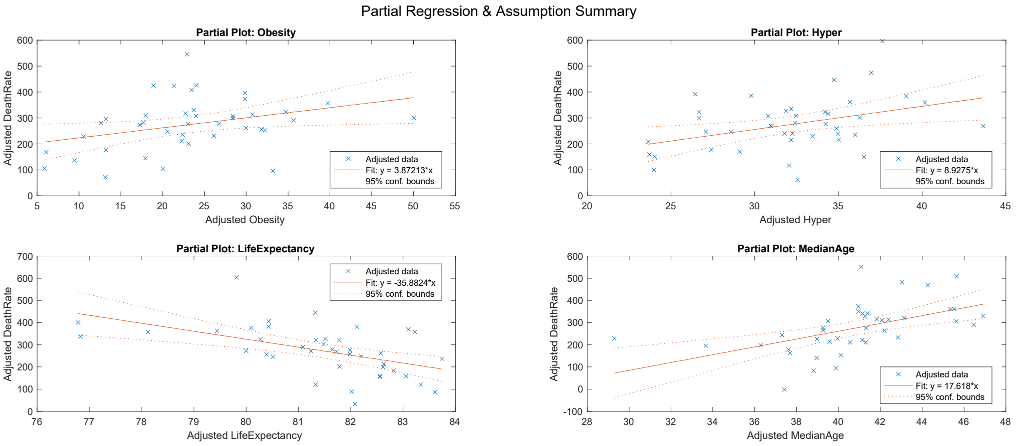

Model Assumption Tests Summary

Durbin-Watson Statistic: 2.150

Breusch–Pagan LM Stat: 2.392 | p-value: 0.6641 → Satisfied

White Test Stat: 14.874 | p-value: 0.3868 → Satisfied

Variance Inflation Factor (VIF) Values:

Predictor	VIF
Obesity	1.12
Hyper	1.91
LifeExpectancy	1.75
MedianAge	1.13

Analysis 3

```
Linear regression model:
    DeathRate ~ 1 + AgeOver65

Estimated Coefficients:
                   Estimate      SE       tStat       pValue  
                   ________    ______    ______    __________

    (Intercept)     43.433     34.824    1.2472        0.2162
    AgeOver65         3304     337.94     9.777    5.024e-15
```

Number of observations: 77, Error degrees of freedom: 75

```
Root Mean Squared Error: 156
R-squared: 0.56,  Adjusted R-Squared: 0.554
F-statistic vs. constant model: 95.6, p-value = 5.02e-15
```

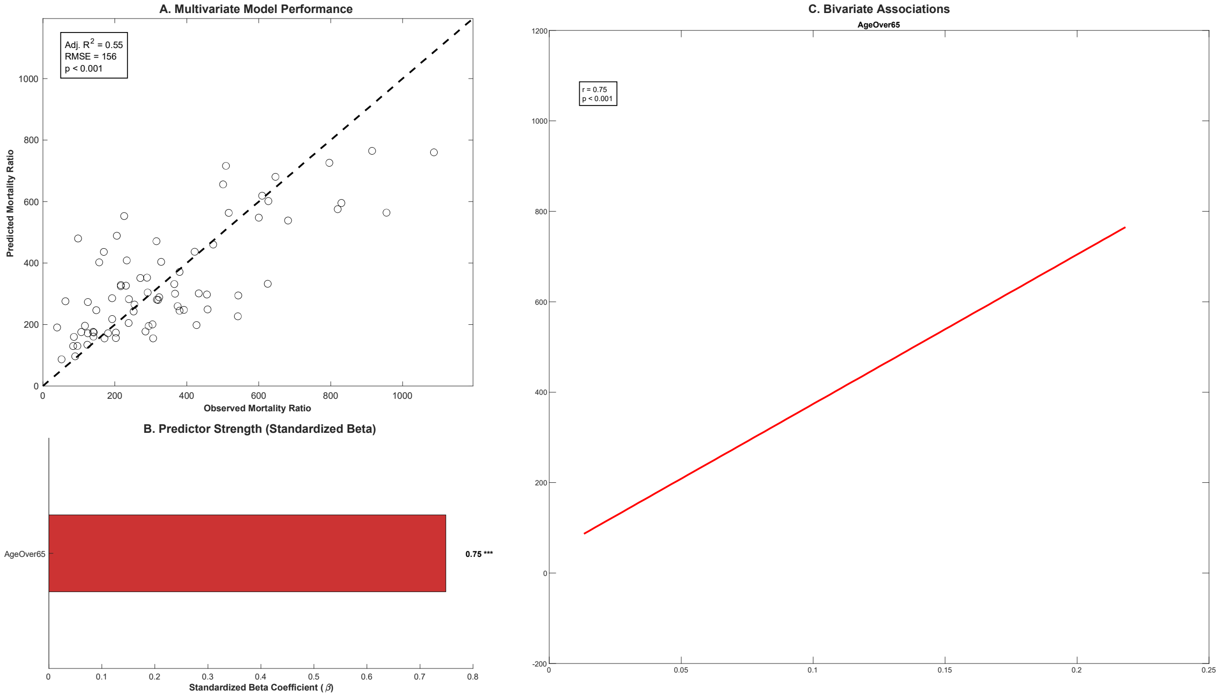

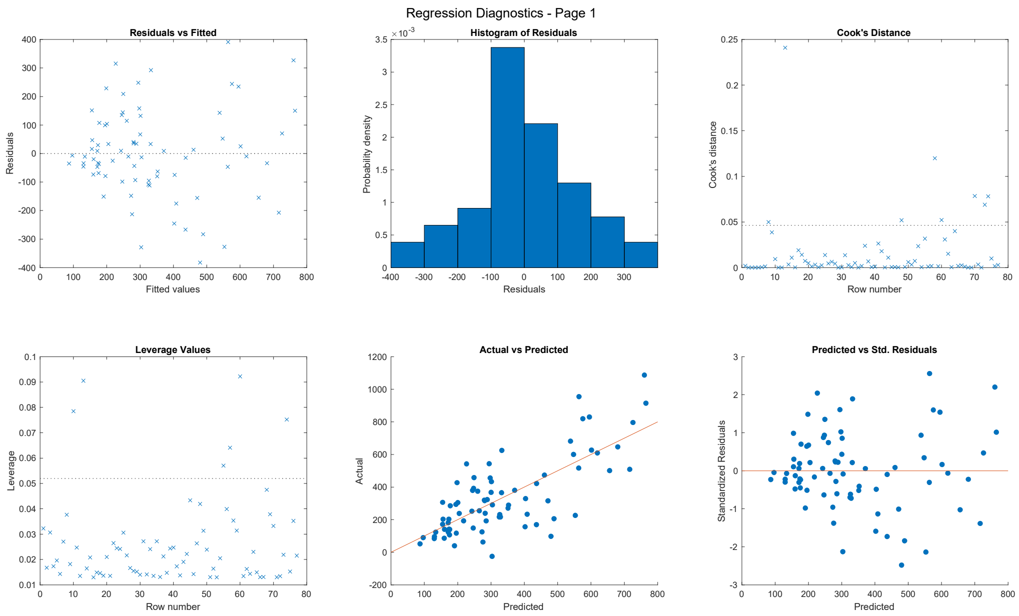

Regression Diagnostics - Page 1
Residuals vs Fitted
Residuals
Fitted values
Histogram of Residuals
Probability density
Residuals
Cook's Distance
Cook's distance
Row number
Leverage Values
Leverage
Row number
Actual vs Predicted
Actual
Predicted
Predicted vs Std. Residuals
Standardized Residuals
Predicted

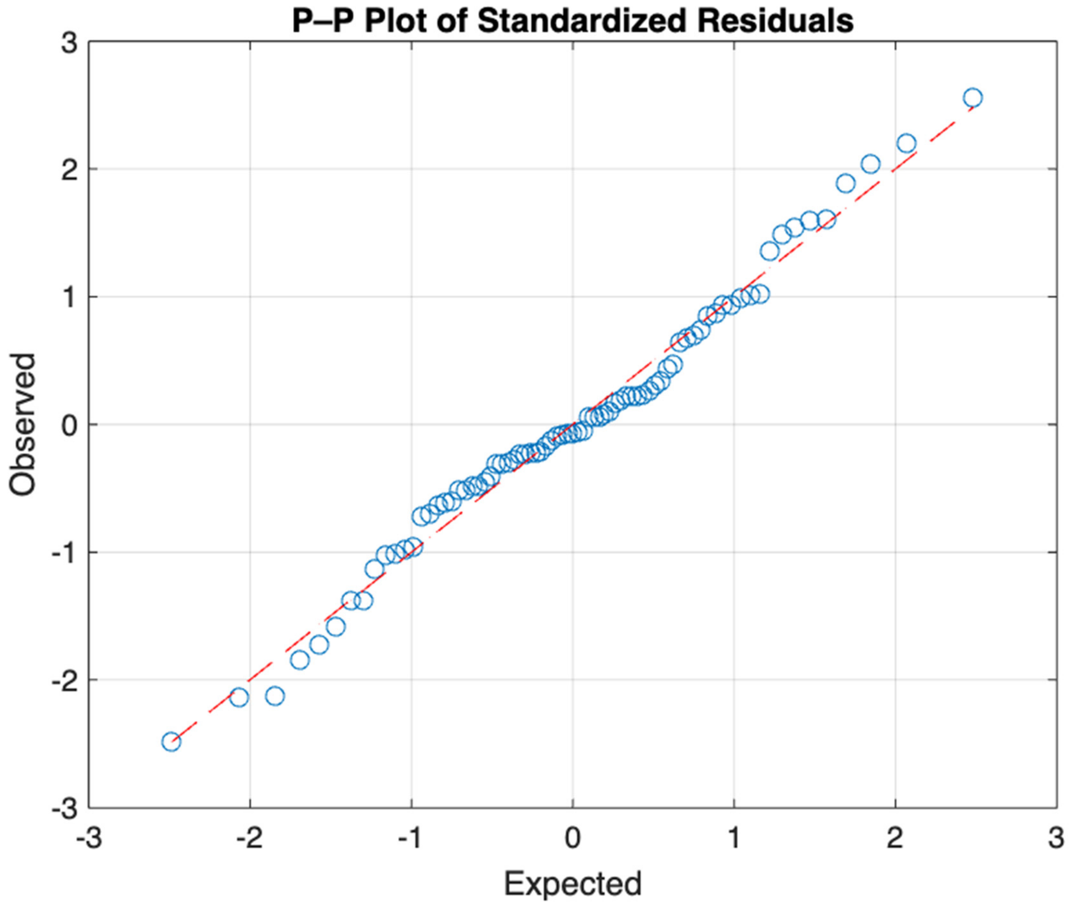

P–P Plot of Standardized Residuals
Observed
Expected

Partial Regression & Assumption Summary

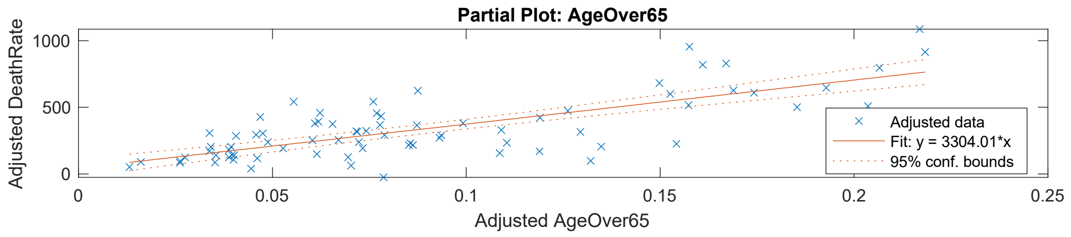

Model Assumption Tests Summary

Durbin-Watson Statistic: 1.892

Breusch–Pagan LM Stat: 9.523 | p-value: 0.0020 → Violated

White Test Stat: 11.156 | p-value: 0.0038 → Violated

Variance Inflation Factor (VIF) Values:

Predictor	VIF
AgeOver65	1.00

Analysis 4

```
Linear regression model (robust fit):
    DeathRate ~ 1 + AgeOver65
```

Estimated Coefficients:

	**Estimate**	**SE**	**tStat**	**pValue**
**(Intercept)**	61.053	19.92	3.0649	0.0033956
**AgeOver65**	3397.8	482.69	7.0394	3.5552e-09

Number of observations: 56, Error degrees of freedom: 54

Root Mean Squared Error: 66.7

R-squared: 0.479, Adjusted R-Squared: 0.469

F-statistic vs. constant model: 49.6, p-value = 3.52e-09

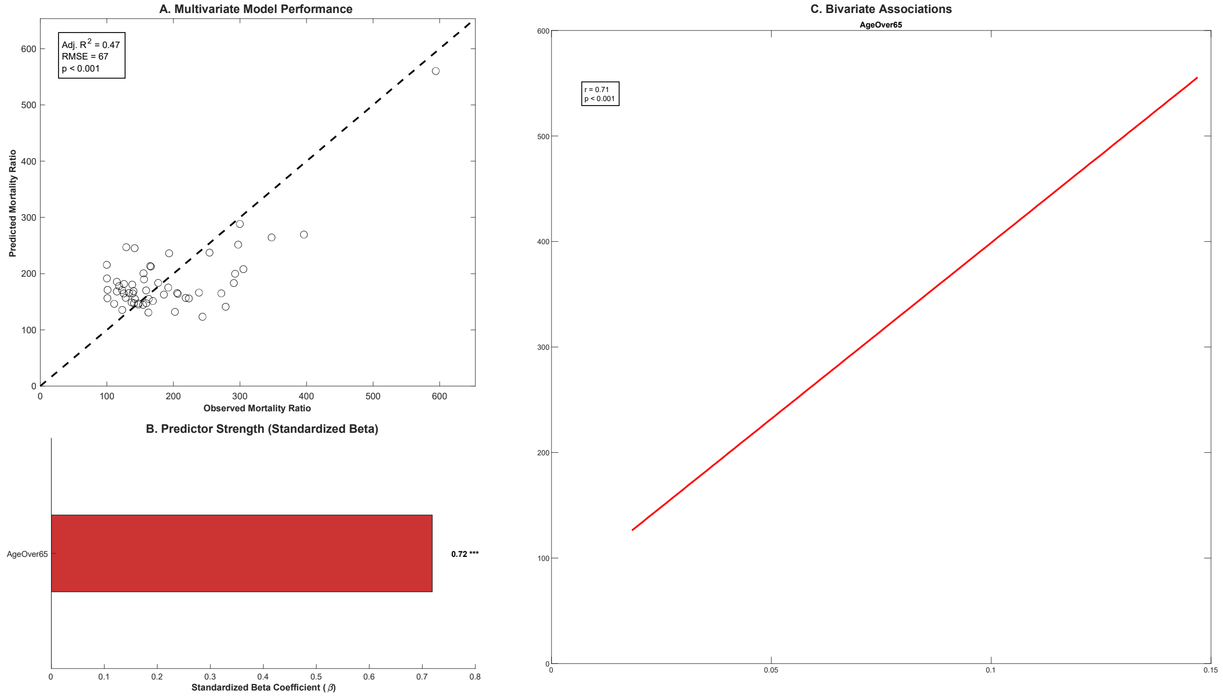

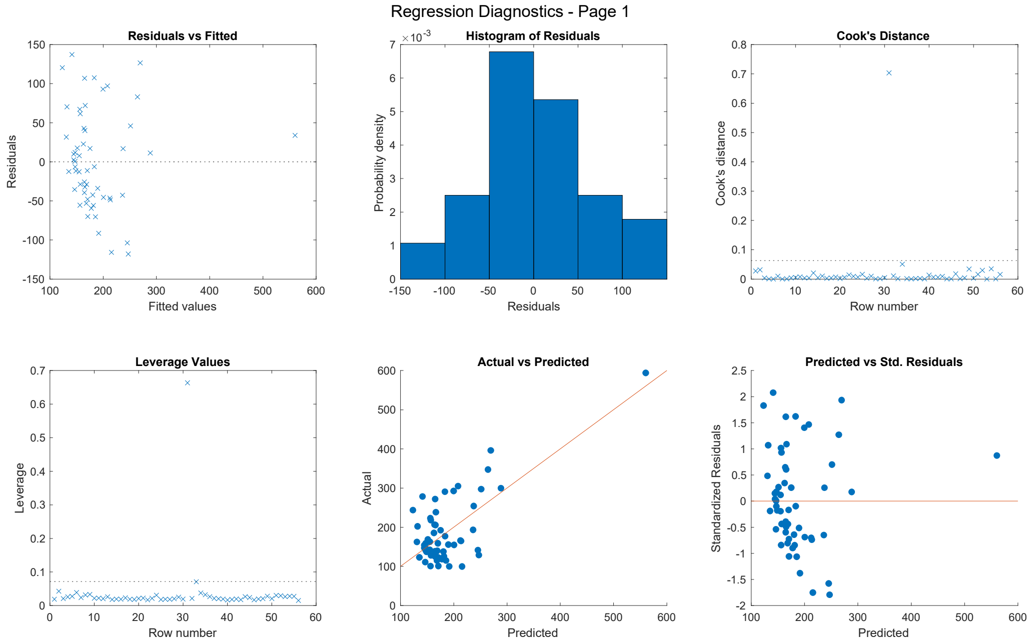
Regression Diagnostics - Page 1
Residuals vs Fitted
Residuals
Fitted values
Histogram of Residuals
Probability density
Residuals
Cook's Distance
Cook's distance
Row number
Leverage Values
Leverage
Row number
Actual vs Predicted
Actual
Predicted
Predicted vs Std. Residuals
Standardized Residuals
Predicted

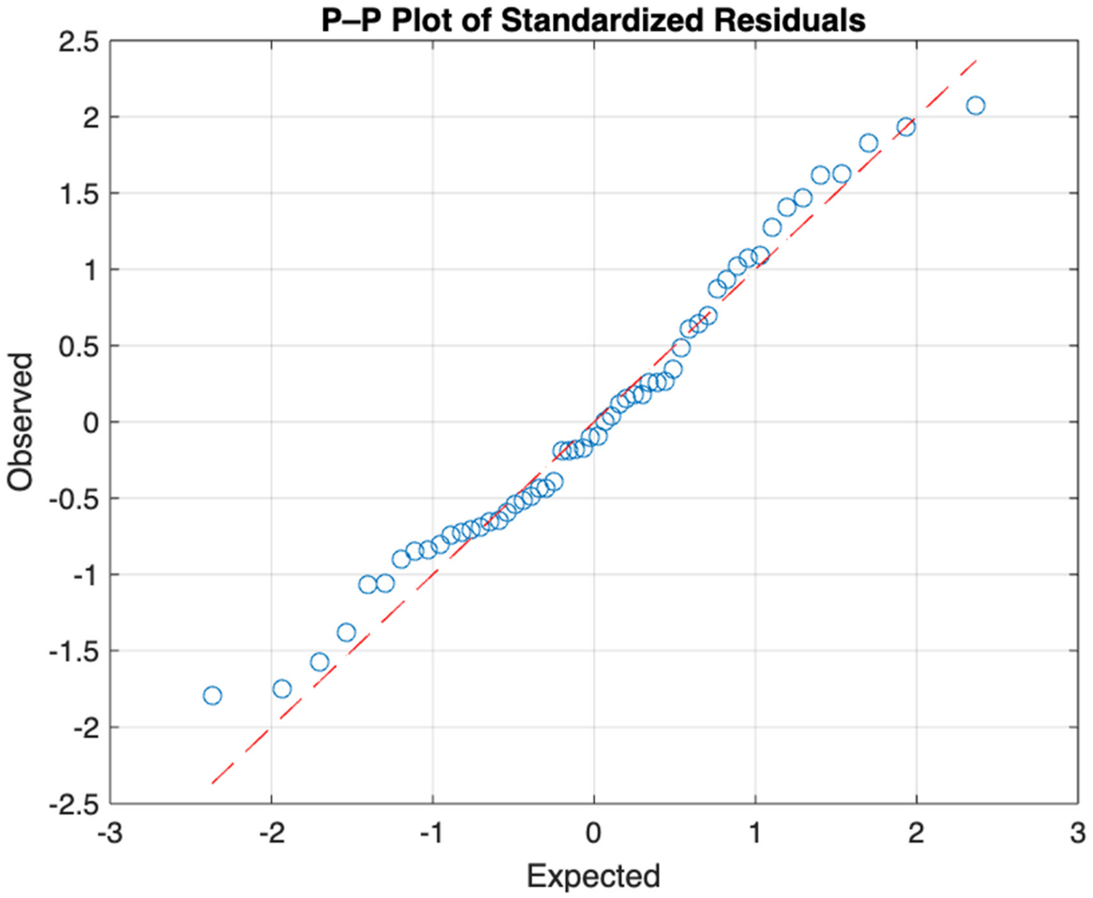
P–P Plot of Standardized Residuals
Observed
Expected

Partial Regression & Assumption Summary

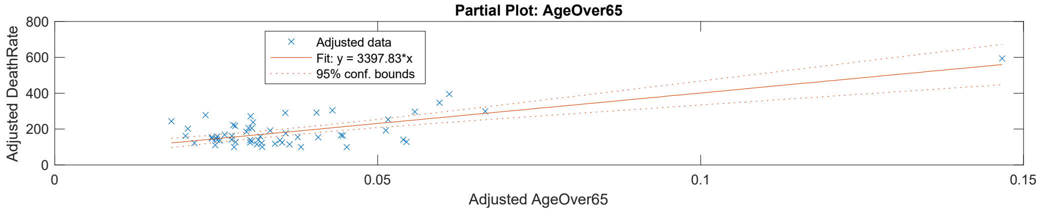

Model Assumption Tests Summary

Durbin-Watson Statistic: 1.690

Breusch–Pagan LM Stat: 0.507 | p-value: 0.4766 → Satisfied

White Test Stat: 3.739 | p-value: 0.1542 → Satisfied

Variance Inflation Factor (VIF) Values:

Predictor	VIF
AgeOver65	1.00

Analysis 5

```
Linear regression model:
    DeathRate ~ 1 + Diabets + Obesity + Gini + GDP + AgeOver65
```

Estimated Coefficients:

	Estimate	SE	tStat	pValue
**(Intercept)**	-1799.8	856.93	-2.1003	0.037945
**Diabets**	-68.348	19.638	-3.4804	0.00071452
**Obesity**	51.848	10	5.1847	9.6933e-07
**Gini**	2016.3	922.95	2.1846	0.031003
**GDP**	-0.01311	0.0040848	-3.2094	0.0017357
**AgeOver65**	14646	1977.8	7.4053	2.6185e-11

Number of observations: 118, Error degrees of freedom: 112

Root Mean Squared Error: 976

R-squared: 0.503, Adjusted R-Squared: 0.481

F-statistic vs. constant model: 22.7, p-value = 1.15e-15

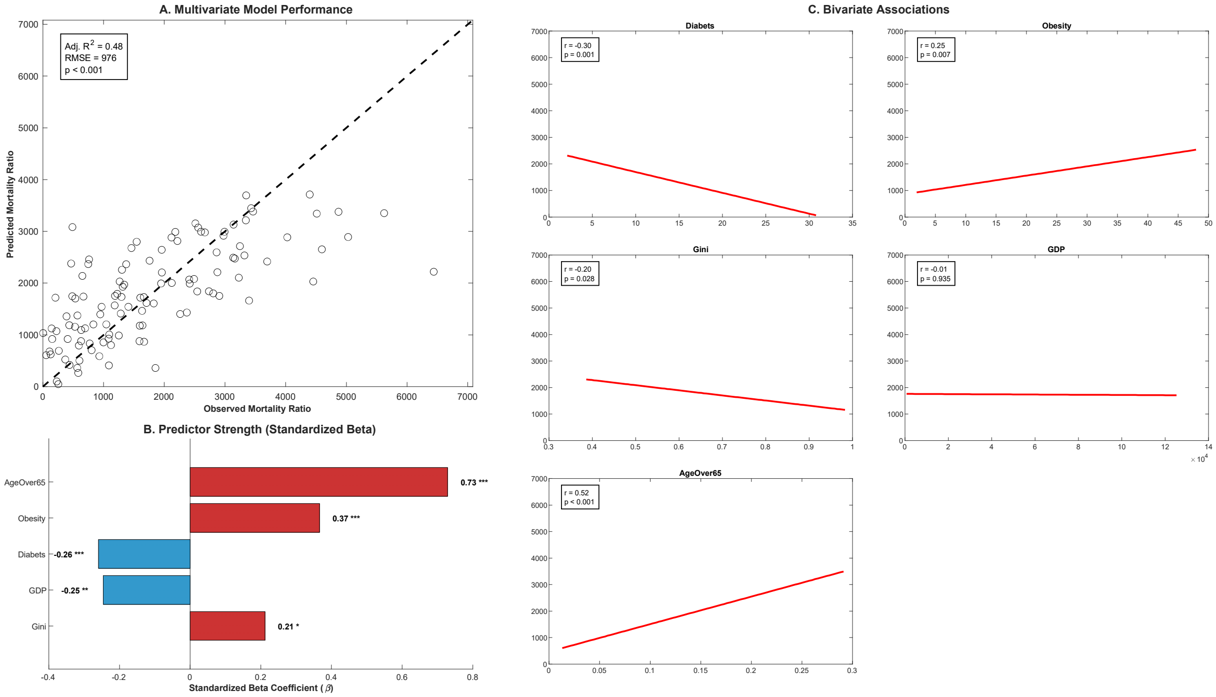

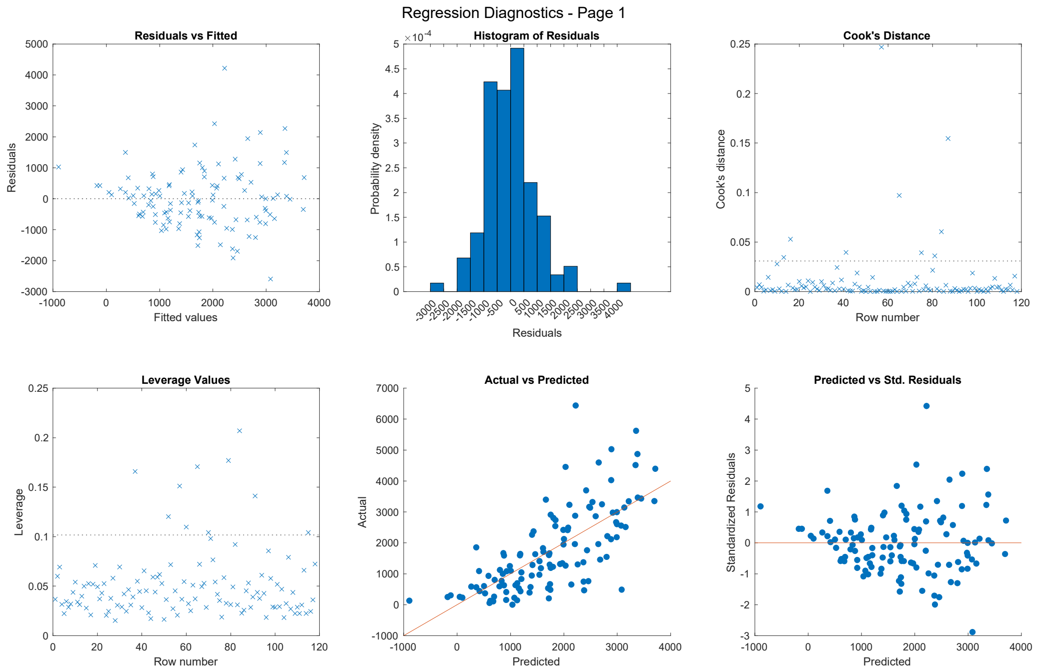
Regression Diagnostics - Page 1
Residuals vs Fitted
Residuals
Fitted values
Histogram of Residuals
Probability density
Residuals
Cook's Distance
Cook's distance
Row number
Leverage Values
Leverage
Row number
Actual vs Predicted
Actual
Predicted
Predicted vs Std. Residuals
Standardized Residuals
Predicted

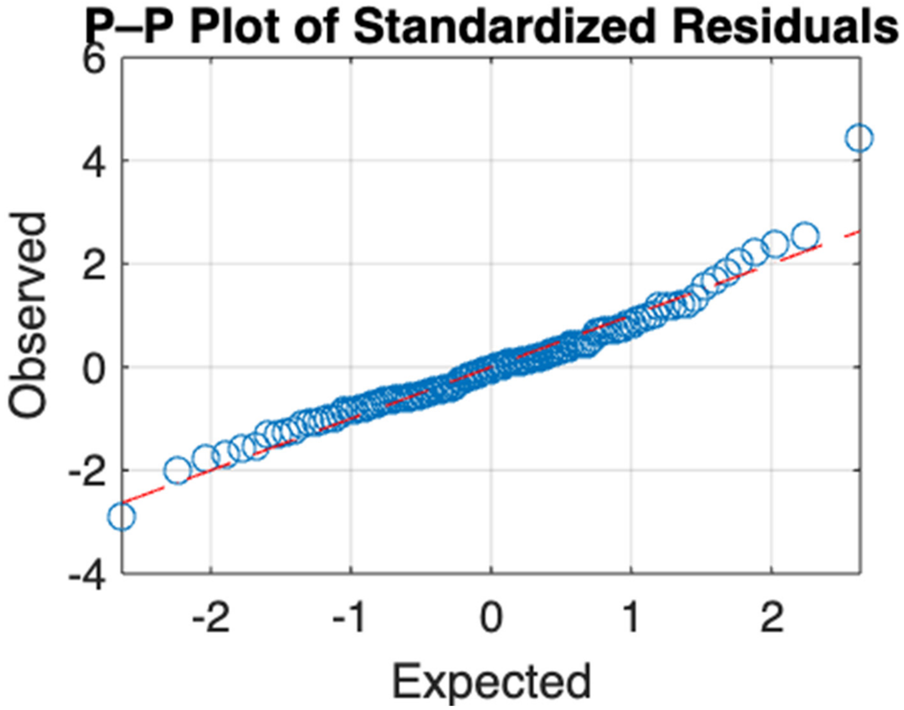
P–P Plot of Standardized Residuals
Observed
Expected

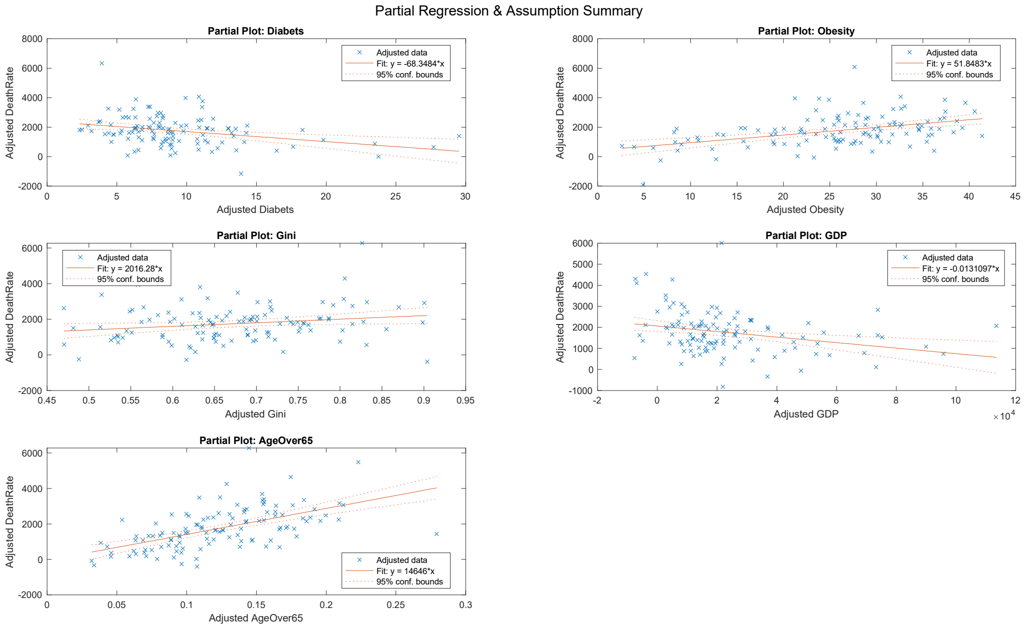

Model Assumption Tests Summary

Durbin-Watson Statistic: 1.986

Breusch–Pagan LM Stat: 7.059 | p-value: 0.2163 → Satisfied

White Test Stat: 31.216 | p-value: 0.0524 → Satisfied

Variance Inflation Factor (VIF) Values:

Predictor	VIF
Diabets	1.25
Obesity	1.13
Gini	2.12
GDP	1.32
AgeOver65	2.18

Analysis 6

Linear regression model (robust fit):
    DeathRate ~ 1 + GDP + Hyper + MedianAge

Estimated Coefficients:

	Estimate	SE	tStat	pValue
**(Intercept)**	-109.05	67.769	-1.6091	0.11098
**GDP**	-0.0023498	0.00053721	-4.374	3.181e-05
**Hyper**	2.9815	1.7737	1.681	0.096125
**MedianAge**	9.5729	1.2822	7.4658	4.3575e-11

Number of observations: 97, Error degrees of freedom: 93
Root Mean Squared Error: 94.7
R-squared: 0.41, Adjusted R-Squared: 0.391
F-statistic vs. constant model: 21.5, p-value = 1.15e-10

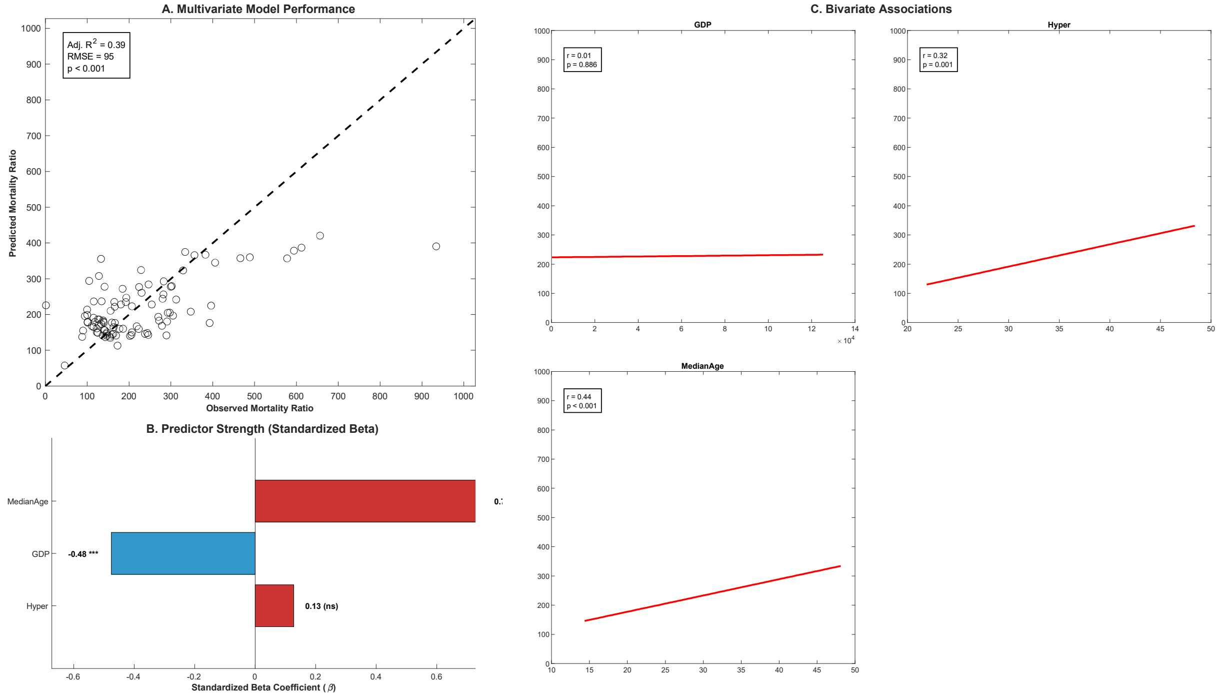

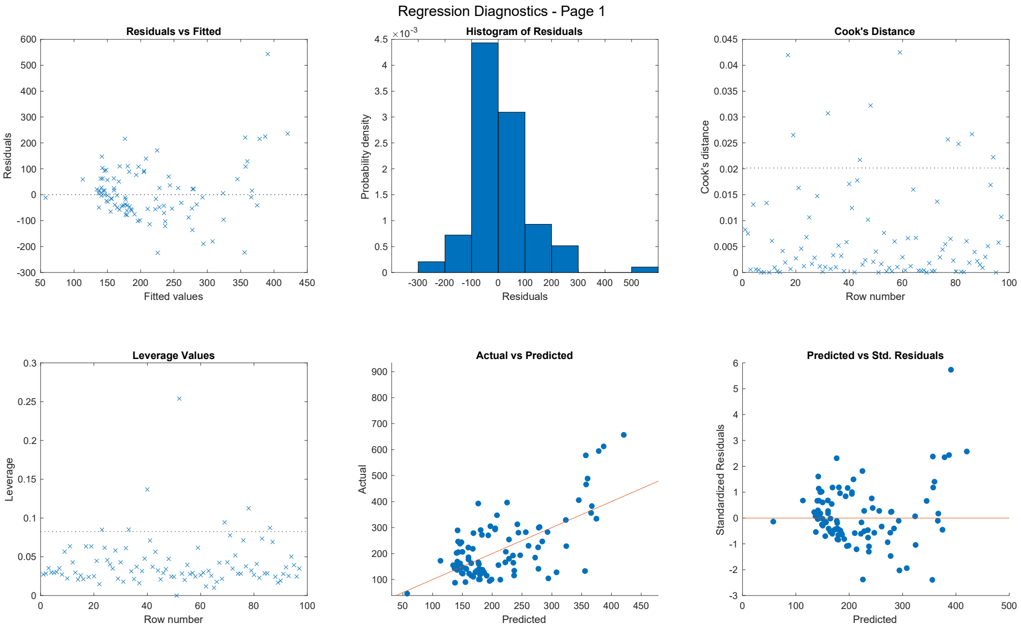
Regression Diagnostics - Page 1
Residuals vs Fitted
Residuals
Fitted values
Histogram of Residuals
Probability density
Residuals
Cook's Distance
Cook's distance
Row number
Leverage Values
Leverage
Row number
Actual vs Predicted
Actual
Predicted
Predicted vs Std. Residuals
Standardized Residuals
Predicted

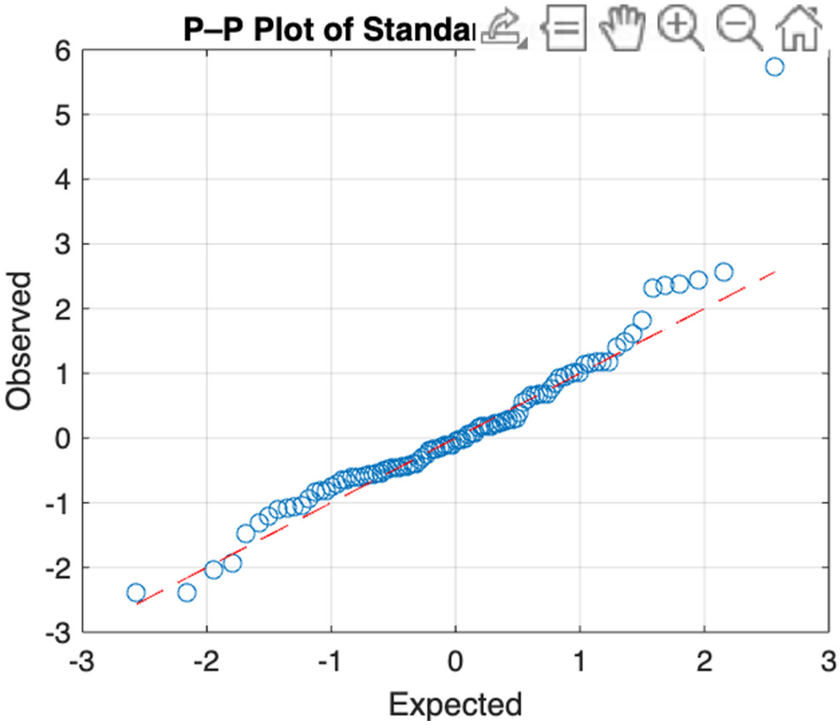
P–P Plot of Standa
Observed
Expected

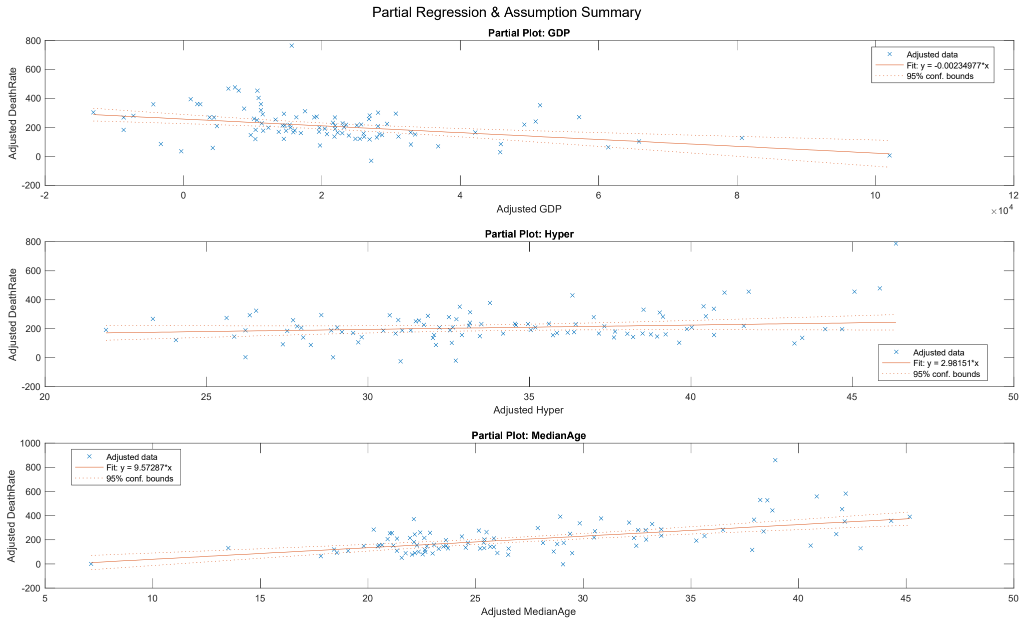

Model Assumption Tests Summary

Durbin-Watson Statistic: 2.073

Breusch–Pagan LM Stat: 20.861 | p-value: 0.0001 → Violated

White Test Stat: 35.305 | p-value: 0.0001 → Violated

Variance Inflation Factor (VIF) Values:

Predictor	VIF
GDP	2.58
Hyper	1.26
MedianAge	2.22

Analysis 7

1. Adding AgeOver65, FStat = 206.8769, pValue = 9.87509e-29
2. Adding Democracy, FStat = 6.1124, pValue = 0.014716

Linear regression model:

DeathRate ~ 1 + Democracy + AgeOver65

Estimated Coefficients:

	Estimate	SE	tStat	pValue
**(Intercept)**	112.86	27.633	4.0844	7.6798e-05
**Democracy**	-14.773	5.9754	-2.4723	0.014716
**AgeOver65**	3421.8	237.4	14.414	9.8828e-29

Number of observations: 133, Error degrees of freedom: 130

Root Mean Squared Error: 123

R-squared: 0.63, Adjusted R-Squared: 0.624

F-statistic vs. constant model: 111, p-value = 9.04e-29

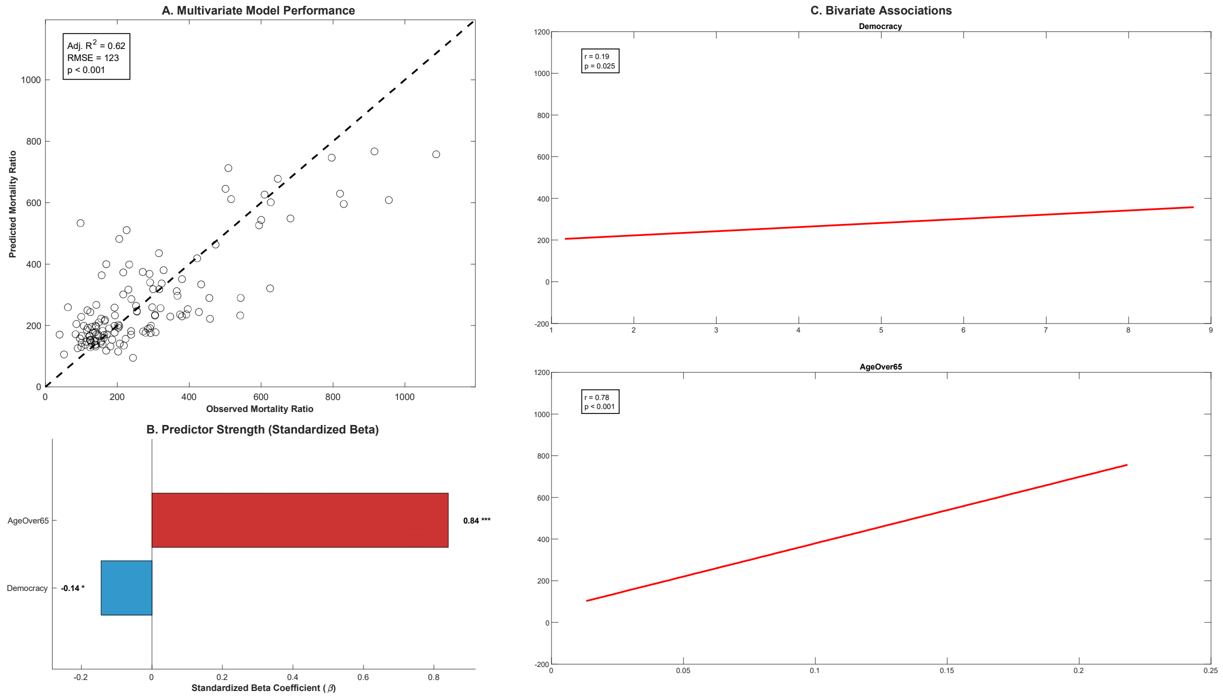

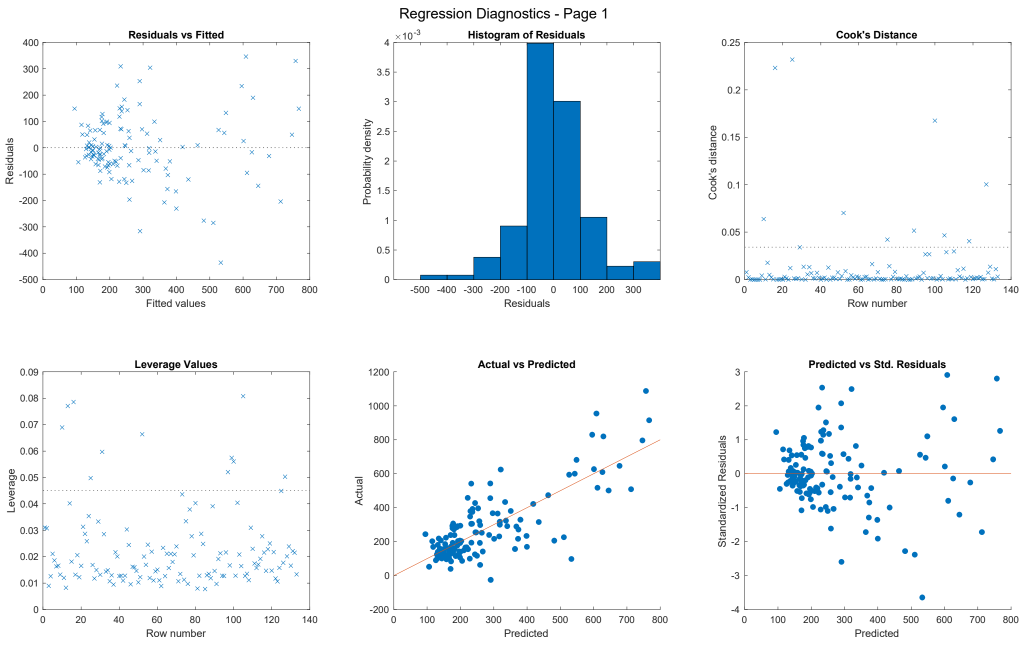
Regression Diagnostics - Page 1
Residuals vs Fitted
Residuals
Fitted values
Histogram of Residuals
Probability density
Residuals
Cook's Distance
Cook's distance
Row number
Leverage Values
Leverage
Row number
Actual vs Predicted
Actual
Predicted
Predicted vs Std. Residuals
Standardized Residuals
Predicted

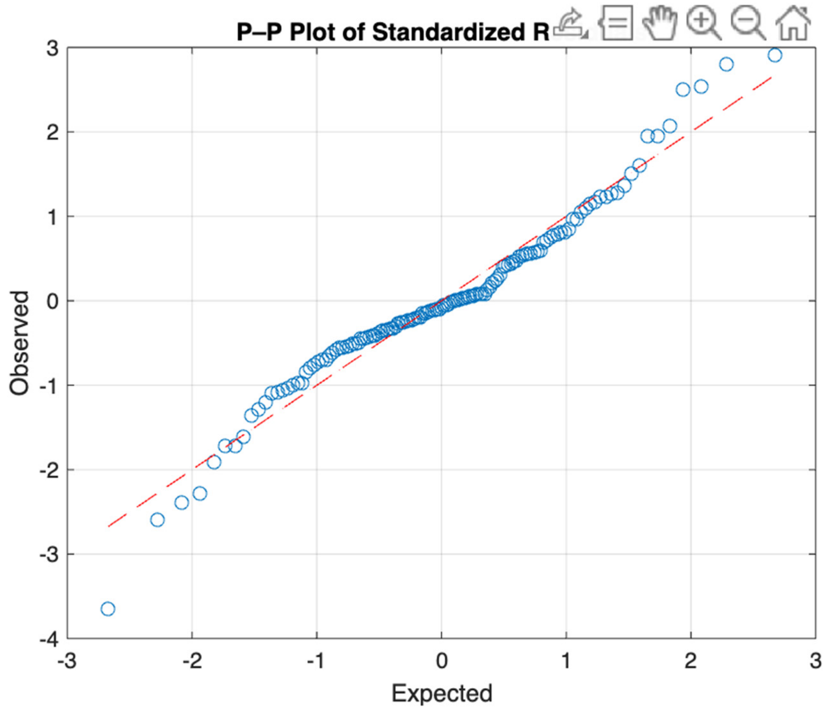
P–P Plot of Standardized R
Observed
Expected

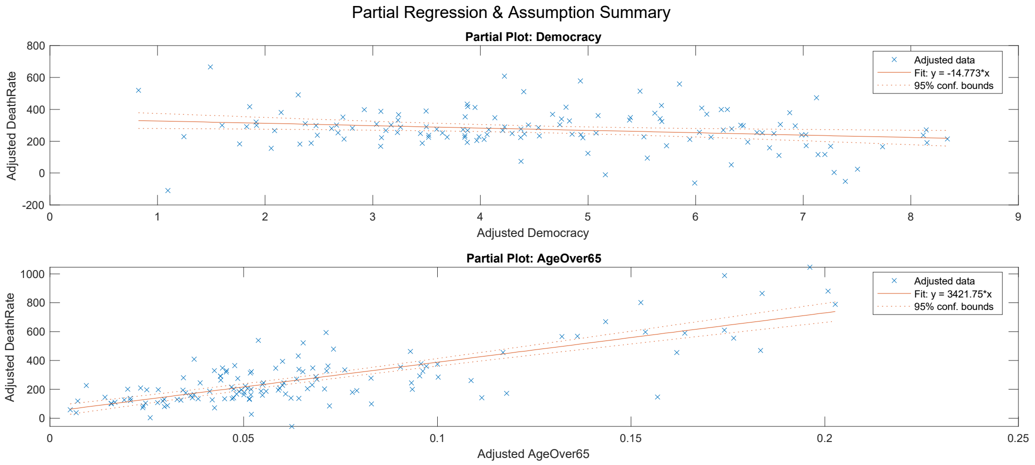

Model Assumption Tests Summary

Durbin-Watson Statistic: 1.979

Breusch–Pagan LM Stat: 23.420 | p-value: 0.0000 → Violated

White Test Stat: 28.827 | p-value: 0.0000 → Violated

Variance Inflation Factor (VIF) Values:

Predictor	VIF
Democracy	1.19
AgeOver65	1.19

Analysis 8

Linear regression model:
    DeathRate ~ 1 + Diabets + Obesity + Democracy + GDP + AgeOver65

Estimated Coefficients:

	Estimate	SE	tStat	pValue
**(Intercept)**	38.756	14.927	2.5963	0.010258
**Diabets**	-2.3765	1.0318	-2.3033	0.022487
**Obesity**	2.2614	0.49452	4.5729	9.2959e-06
**Democracy**	-10.476	2.8365	-3.6933	0.00029903
**GDP**	-0.0010265	0.00025649	-4.0023	9.3939e-05
**AgeOver65**	696.01	97.612	7.1303	2.835e-11

Number of observations: 174, Error degrees of freedom: 168
Root Mean Squared Error: 61.3
R-squared: 0.353, Adjusted R-Squared: 0.334
F-statistic vs. constant model: 18.3, p-value = 1.68e-14

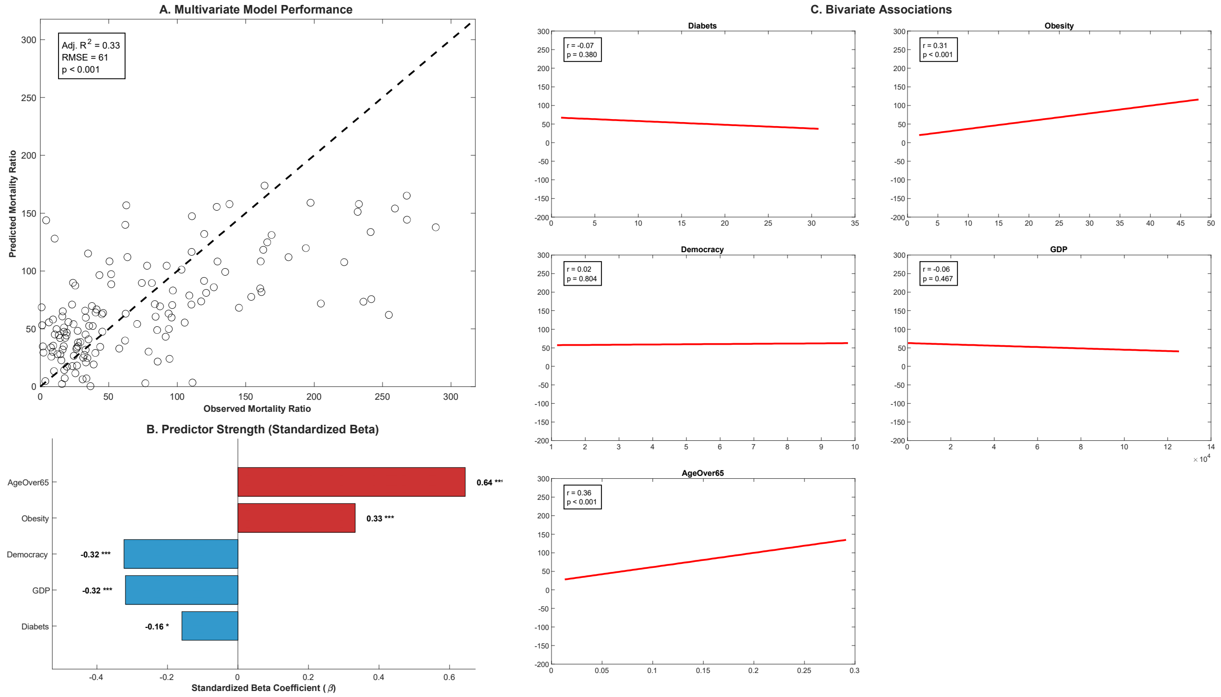

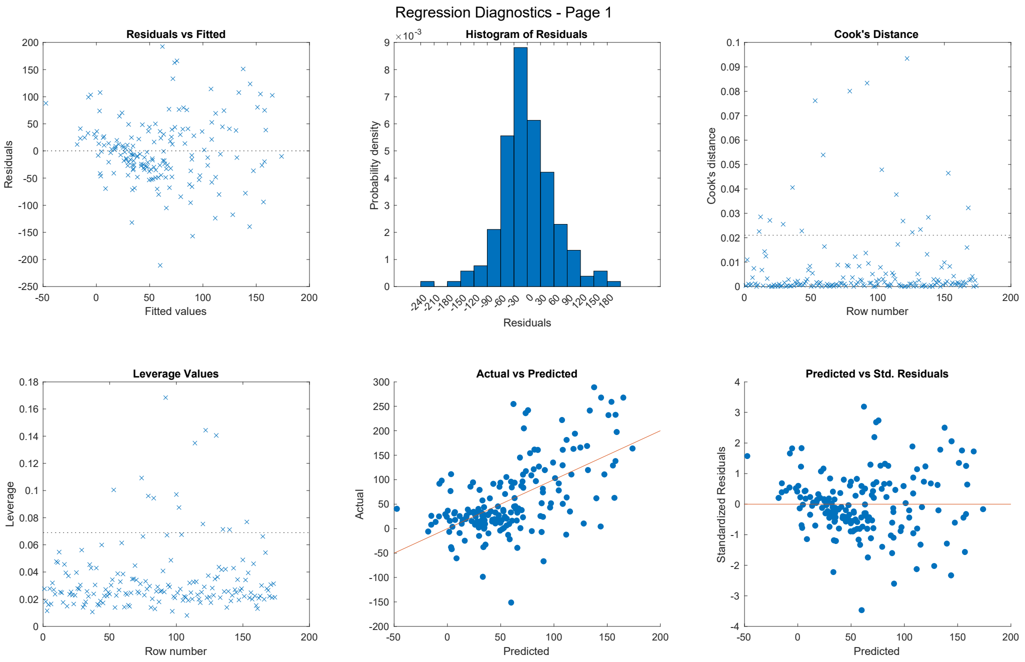
Regression Diagnostics - Page 1
Residuals vs Fitted
Residuals
Fitted values
Histogram of Residuals
Probability density
Residuals
Cook's Distance
Cook's distance
Row number
Leverage Values
Leverage
Row number
Actual vs Predicted
Actual
Predicted
Predicted vs Std. Residuals
Standardized Residuals
Predicted

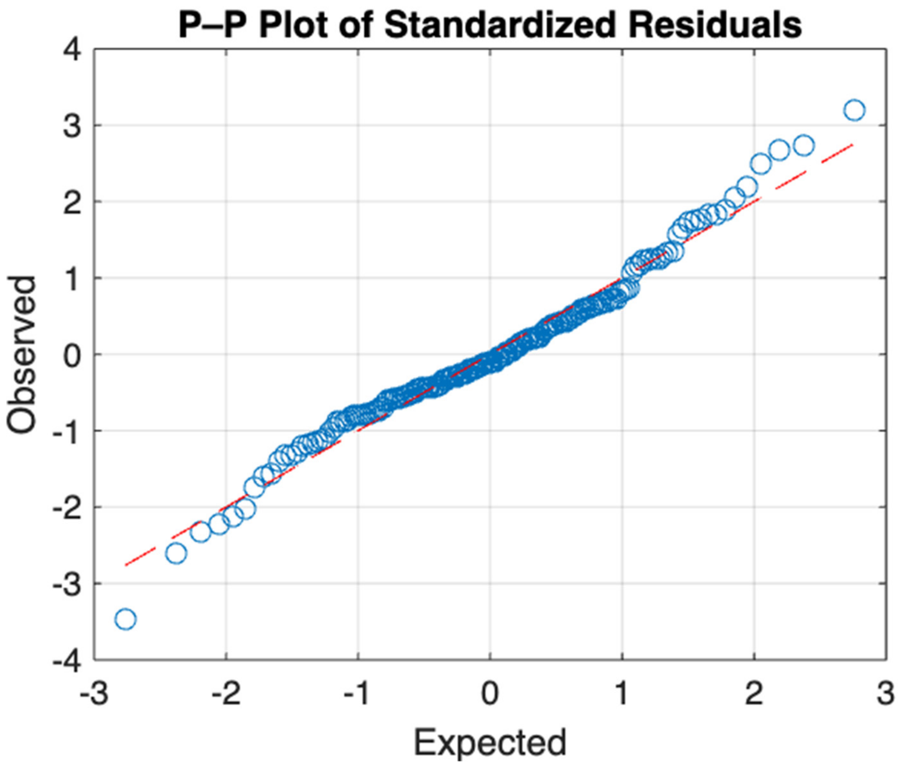
P–P Plot of Standardized Residuals
Observed
Expected

Partial Regression & Assumption Summary

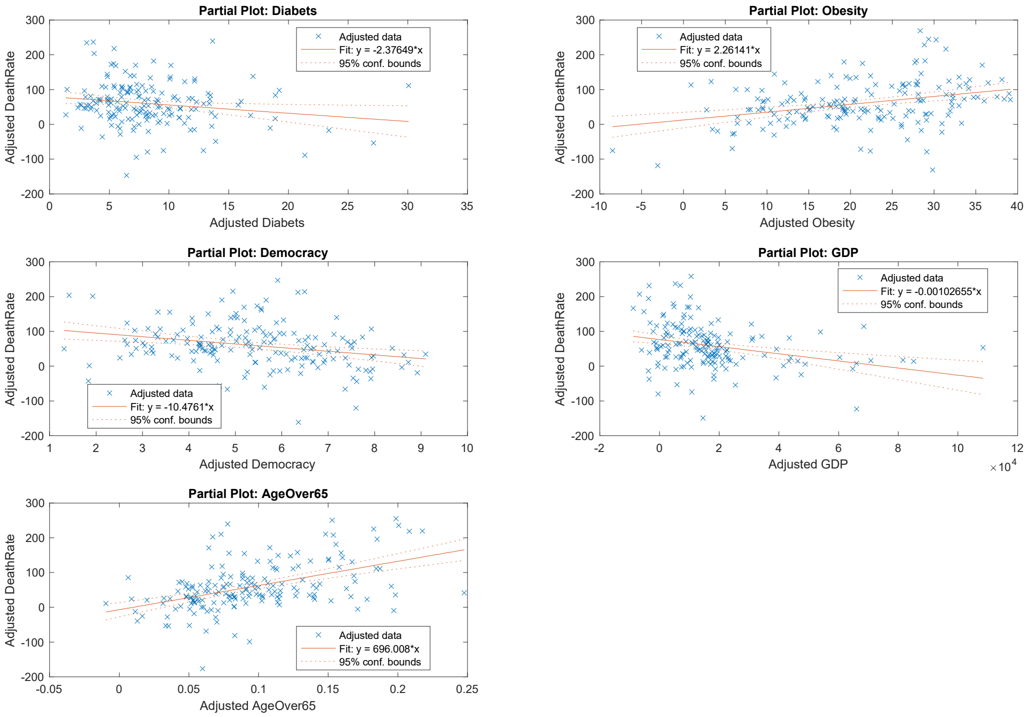

Model Assumption Tests Summary

Durbin-Watson Statistic: 1.928

Breusch–Pagan LM Stat: 16.626 | p-value: 0.0053 → Violated

White Test Stat: 26.720 | p-value: 0.1433 → Satisfied

Variance Inflation Factor (VIF) Values:

Predictor	VIF
Diabets	1.24
Obesity	1.37
Democracy	1.99
GDP	1.65
AgeOver65	2.12

Analysis 9

1. Adding Hyper, FStat = 47.5269, pValue = 9.87884e-11
2. Adding AgeOver65, FStat = 38.8403, pValue = 3.47017e-09
3. Adding GDP, FStat = 37.6757, pValue = 5.70669e-09
4. Adding Obesity, FStat = 7.9157, pValue = 0.0054819

Linear regression model (robust fit):
    DeathRate ~ 1 + Obesity + GDP + Hyper + AgeOver65

Estimated Coefficients:

	**Estimate**	**SE**	**tStat**	**pValue**
**(Intercept)**	-128.22	35.379	-3.6242	0.00038317
**Obesity**	1.6502	0.59208	2.7871	0.0059273
**GDP**	-0.0022462	0.00033217	-6.7621	2.1205e-10
**Hyper**	4.9591	0.98196	5.0502	1.1348e-06
**AgeOver65**	842.23	104.6	8.0519	1.391e-13

Number of observations: 174, Error degrees of freedom: 169
Root Mean Squared Error: 75.8
R-squared: 0.503, Adjusted R-Squared: 0.491
F-statistic vs. constant model: 42.7, p-value = 1.02e-24

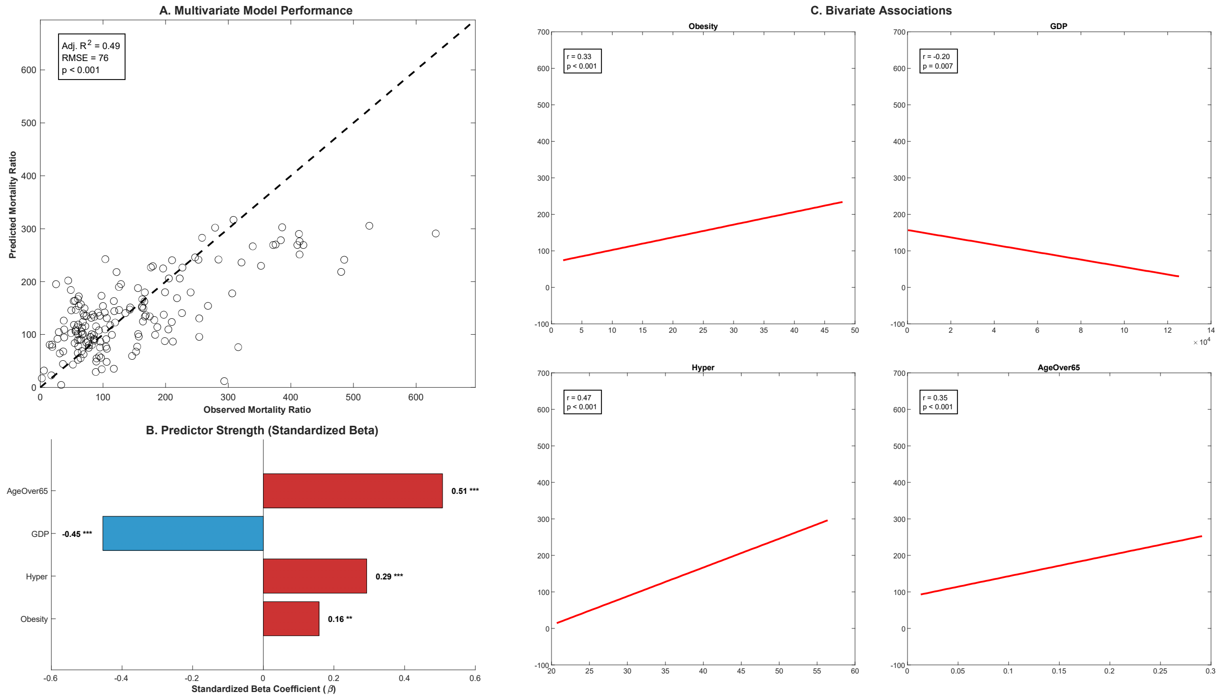

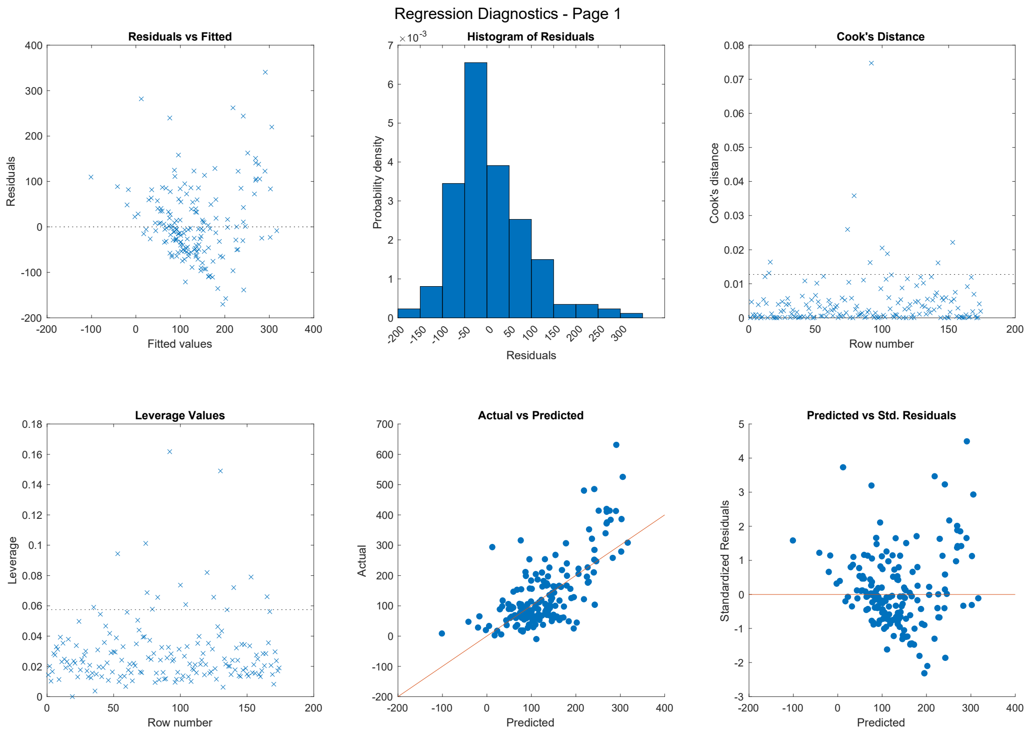
Regression Diagnostics - Page 1
Residuals vs Fitted
Residuals
Fitted values
Histogram of Residuals
Probability density
Residuals
Cook's Distance
Cook's distance
Row number
Leverage Values
Leverage
Row number
Actual vs Predicted
Actual
Predicted
Predicted vs Std. Residuals
Standardized Residuals
Predicted

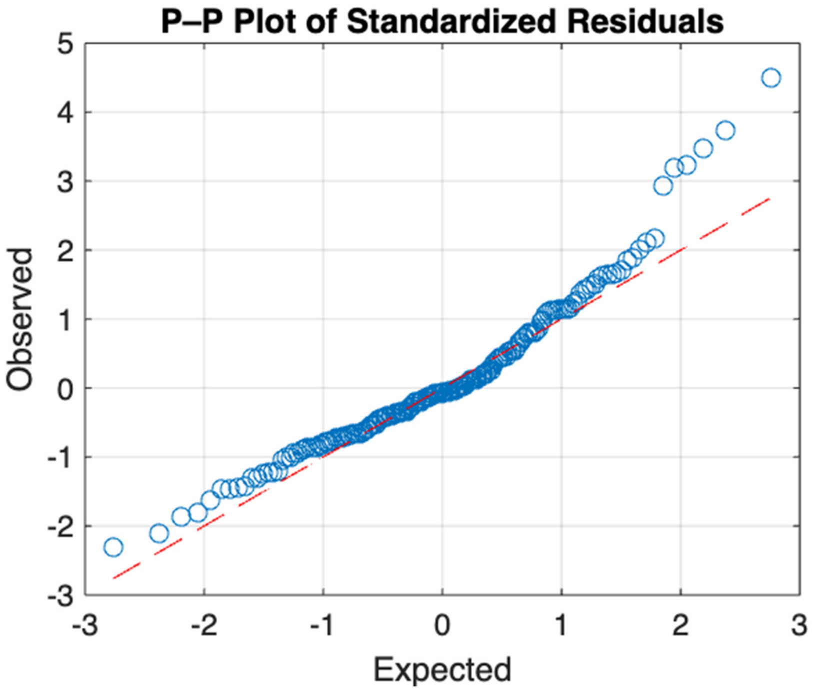
P–P Plot of Standardized Residuals
Observed
Expected

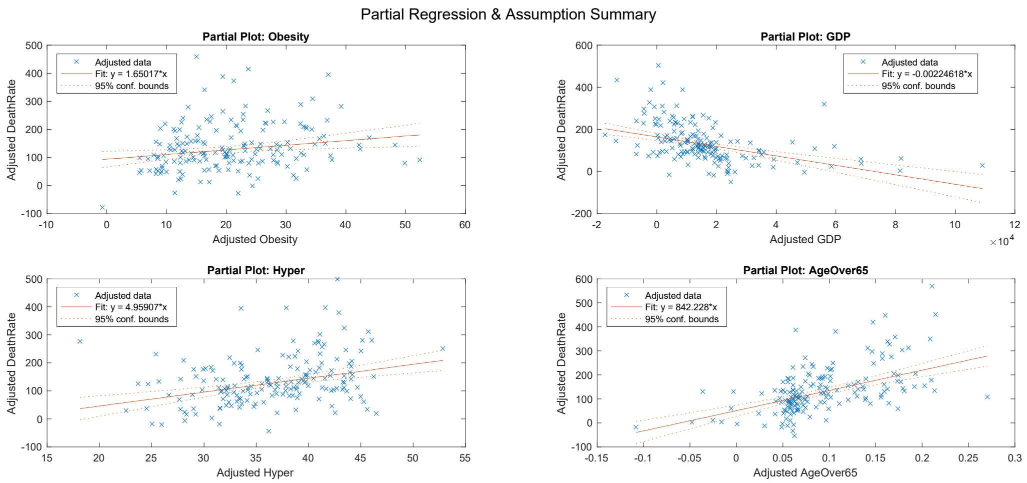

Model Assumption Tests Summary

Durbin-Watson Statistic: 1.955

Breusch–Pagan LM Stat: 20.543 | p-value: 0.0004 → Violated

White Test Stat: 53.630 | p-value: 0.0000 → Violated

Variance Inflation Factor (VIF) Values:

Predictor	VIF
Obesity	1.29
GDP	1.81
Hyper	1.35
AgeOver65	1.59

Analysis 10

```
Linear regression model (robust fit):
    DeathRate ~ 1 + LifeExpectancy + LungDiseases + AgeOver65

Estimated Coefficients:
```

	Estimate	SE	tStat	pValue
**(Intercept)**	136.18	27.075	5.0299	1.2384e-06
**LifeExpectancy**	-1.4777	0.41974	-3.5204	0.00055308
**LungDiseases**	-189.83	111.51	-1.7024	0.090513
**AgeOver65**	556.23	58.912	9.4416	2.8206e-17

Number of observations: 174, Error degrees of freedom: 170

```
Root Mean Squared Error: 31.7
R-squared: 0.439,  Adjusted R-Squared: 0.429
F-statistic vs. constant model: 44.4, p-value = 3.17e-21
```

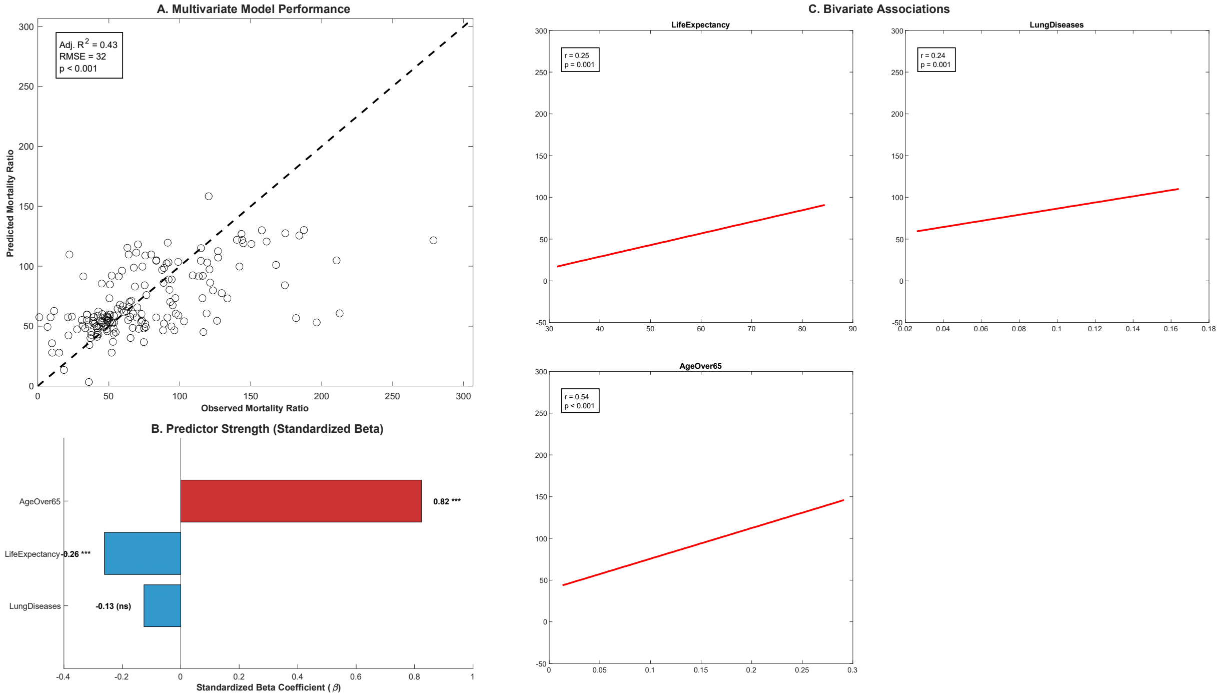

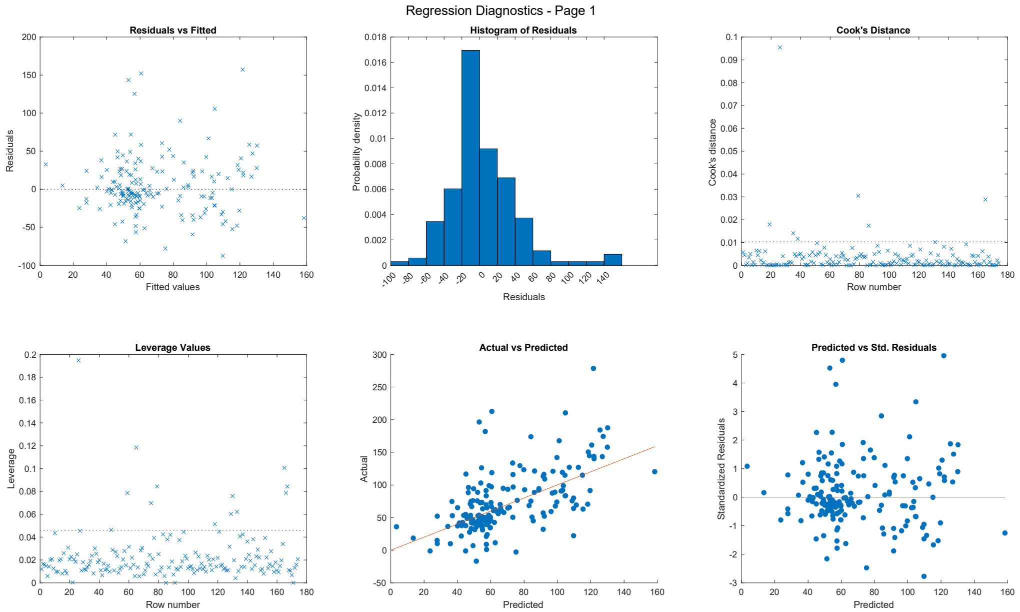
Regression Diagnostics - Page 1
Residuals vs Fitted
Residuals
Fitted values
Histogram of Residuals
Probability density
Residuals
Cook's Distance
Cook's distance
Row number
Leverage Values
Leverage
Row number
Actual vs Predicted
Actual
Predicted
Predicted vs Std. Residuals
Standardized Residuals
Predicted

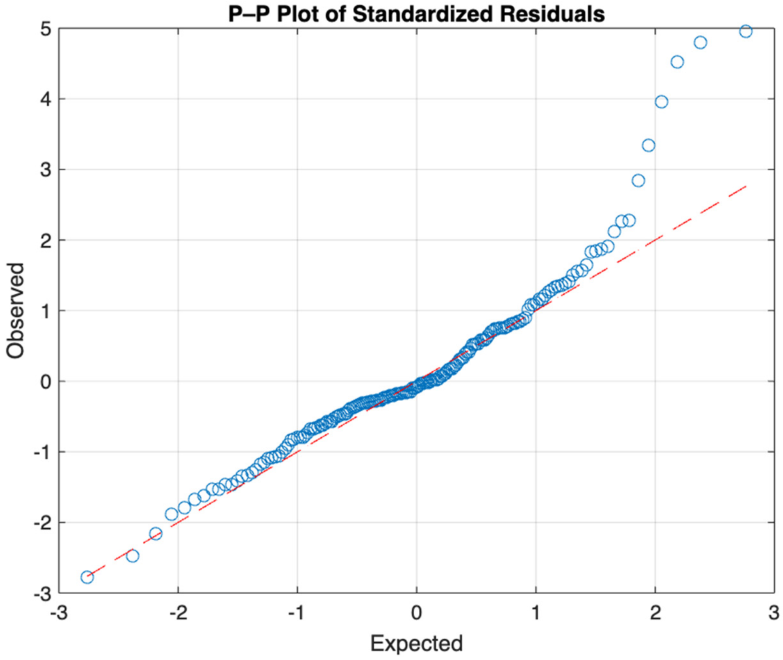
P–P Plot of Standardized Residuals
Observed
Expected

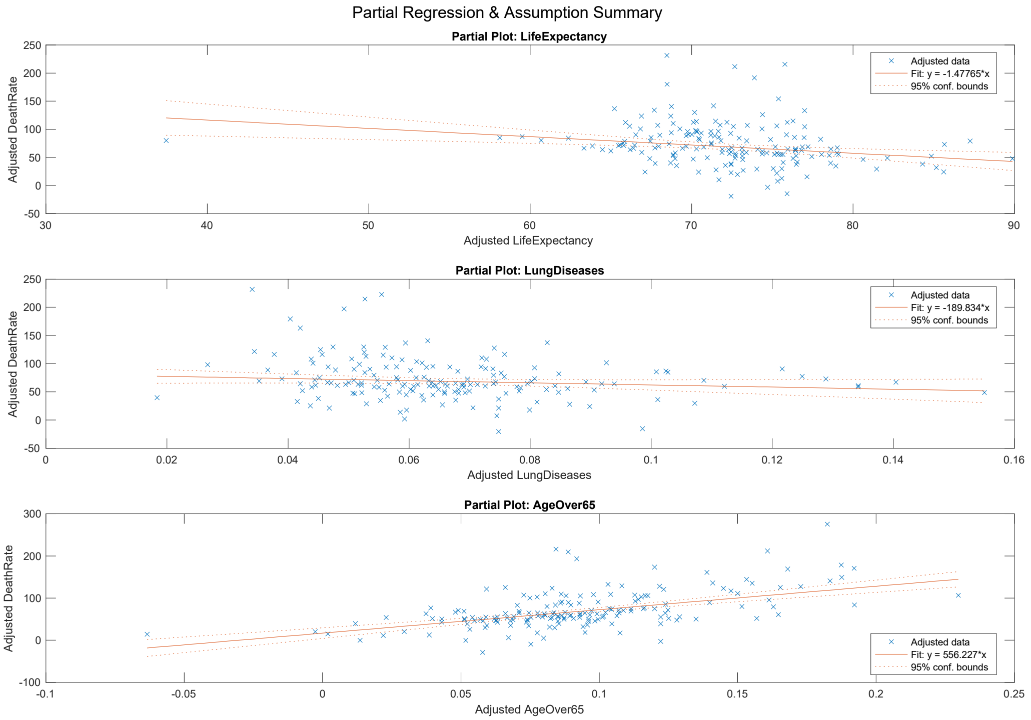

Model Assumption Tests Summary

Durbin-Watson Statistic: 2.160

Breusch–Pagan LM Stat: 7.514 | p-value: 0.0572 → Satisfied

White Test Stat: 12.685 | p-value: 0.1774 → Satisfied

Variance Inflation Factor (VIF) Values:

Predictor	VIF
LifeExpectancy	2.10
LungDiseases	2.08
AgeOver65	2.89
