# Supplementary material for: An Ecological Study on the Mortality Impact of the COVID-19 Pandemic According to Country Development Status and Pandemic Years
Source: Epidemiologia (Basel). 2026 Apr 6;7(2):50. doi: 10.3390/epidemiologia7020050 (PMC13115391; doi:10.3390/epidemiologia7020050)
Supplement: Supplementary file 1 [file epidemiologia-07-00050-s001.zip › Sensitivity Analysis Regression Models Excluding Referenced Countries (N=165).pdf]

# Regression Results and Diagnostics for 165 Countries

## Analysis 1 Model A

Linear regression model (robust fit):
DeathRate ~ 1 + Diabetes + Obesity + Gini + GDP + Hyper + AgeOver65

Estimated Coefficients:

| | Estimate | SE | tStat | pValue |
|---|---|---|---|---|
| **(Intercept)** | -3141.1 | 605.23 | -5.19 | 6.3804e-07 |
| **Diabetes** | -29.91 | 12.674 | -2.3599 | 0.019501 |
| **Obesity** | 30.326 | 6.6273 | 4.5759 | 9.5379e-06 |
| **Gini** | 1895.9 | 576.79 | 3.287 | 0.0012477 |
| **GDP** | -0.0069549 | 0.0032377 | -2.1481 | 0.033233 |
| **Hyper** | 34.383 | 9.7789 | 3.516 | 0.00057188 |
| **AgeOver65** | 15636 | 1208.9 | 12.934 | 1.4713e-26 |

Number of observations: 165, Error degrees of freedom: 158
Root Mean Squared Error: 717
R-squared: 0.702, Adjusted R-Squared: 0.69
F-statistic vs. constant model: 61.9, p-value = 5.34e-39

## Analysis 1 Model B

Linear regression model:
DeathRate ~ 1 + Obesity + Diabetes*Hyper + Cardio*Hyper + Cardio*Unemployment + Democracy*GDP + Democracy*GII + Democracy*MedianAge + Democracy*AgeOver65 + Density*IHDI + Density*Hyper + Density*AgeOver65 + GII*MedianAge + Hyper*LifeExpectancy + Hyper*AgeOver65 + MedianAge*Unemployment

Estimated Coefficients:

| | Estimate | SE | tStat | pValue |
|---|---|---|---|---|
| **(Intercept)** | -10389 | 6648 | -1.5627 | 0.12042 |
| **Diabetes** | -214.24 | 79.933 | -2.6803 | 0.0082586 |
| **Obesity** | 21.907 | 8.1068 | 2.7023 | 0.007758 |
| **Cardio** | 33800 | 8823.1 | 3.8308 | 0.00019377 |
| **Democracy** | -871.73 | 261.98 | -3.3275 | 0.0011253 |
| **Density** | 5.1991 | 1.9514 | 2.6643 | 0.0086407 |
| **GDP** | 0.053747 | 0.017976 | 2.9899 | 0.0033096 |

| | | | | |
|---|---|---|---|---|
| **IHDI** | 13.136 | 14.833 | 0.88561 | 0.37738 |
| **GII** | -32.996 | 9.838 | -3.3539 | 0.0010308 |
| **Hyper** | 402.19 | 168.92 | 2.3809 | 0.018646 |
| **LifeExpectancy** | 228.86 | 98.323 | 2.3276 | 0.021397 |
| **MedianAge** | -249.55 | 69.033 | -3.6149 | 0.00042116 |
| **AgeOver65** | -4577.6 | 14719 | -0.31101 | 0.75627 |
| **Unemployment** | -169.97 | 47.605 | -3.5705 | 0.00049197 |
| **Diabetes:Hyper** | 5.0195 | 2.1614 | 2.3224 | 0.021686 |
| **Cardio:Hyper** | -955.69 | 248.74 | -3.8422 | 0.00018581 |
| **Cardio:Unemployment** | 453.02 | 271.68 | 1.6675 | 0.097697 |
| **Democracy:GDP** | -0.0062408 | 0.0021293 | -2.9309 | 0.0039609 |
| **Democracy:GII** | 3.376 | 1.3575 | 2.4869 | 0.014087 |
| **Democracy:MedianAge** | 41.802 | 11.109 | 3.7629 | 0.00024815 |
| **Democracy:AgeOver65** | -4234.7 | 1413.4 | -2.9961 | 0.0032472 |
| **Density:IHDI** | -0.081429 | 0.037409 | -2.1768 | 0.031212 |
| **Density:Hyper** | -0.092126 | 0.033129 | -2.7809 | 0.0061853 |
| **Density:AgeOver65** | -12.009 | 5.3572 | -2.2416 | 0.026593 |
| **GII:MedianAge** | 0.9687 | 0.36315 | 2.6675 | 0.0085633 |
| **Hyper:LifeExpectancy** | -6.1259 | 2.4717 | -2.4784 | 0.014412 |
| **Hyper:AgeOver65** | 1023.1 | 229.35 | 4.4608 | 1.6872e-05 |
| **MedianAge:Unemployment** | 6.1661 | 1.442 | 4.2762 | 3.5417e-05 |

Number of observations: 165, Error degrees of freedom: 137
Root Mean Squared Error: 690
R-squared: 0.791, Adjusted R-Squared: 0.75
F-statistic vs. constant model: 19.2, p-value = 2.38e-34

## Analysis 2 Model A

Linear regression model:
    DeathRate ~ 1 + Hyper + LifeExpectancy + LungDiseases + Unemployment

Estimated Coefficients:

| | Estimate | SE | tStat | pValue |
|---|---|---|---|---|
| **(Intercept)** | 12058 | 6165.4 | 1.9558 | 0.058289 |
| **Hyper** | 84.161 | 23.919 | 3.5186 | 0.0011951 |
| **LifeExpectancy** | -184.6 | 70.213 | -2.6291 | 0.01251 |
| **LungDiseases** | 14887 | 4005.1 | 3.717 | 0.00068239 |
| **Unemployment** | 124.52 | 40.238 | 3.0945 | 0.0038015 |

Number of observations: 41, Error degrees of freedom: 36
Root Mean Squared Error: 722
R-squared: 0.627, Adjusted R-Squared: 0.586
F-statistic vs. constant model: 15.1, p-value = 2.39e-07

## Analysis 3 Model A

Linear regression model:
    DeathRate ~ 1 + Diabets + Obesity + Gini + AgeOver65

Estimated Coefficients:

| | Estimate | SE | tStat | pValue |
|---|---|---|---|---|
| **(Intercept)** | -3220.1 | 1098.4 | -2.9316 | 0.00461 |
| **Diabets** | -60.883 | 24.782 | -2.4567 | 0.016619 |
| **Obesity** | 42.738 | 13.44 | 3.1799 | 0.0022318 |
| **Gini** | 3390.3 | 1201.9 | 2.8207 | 0.0062982 |
| **AgeOver65** | 20644 | 2780.4 | 7.4248 | 2.6178e-10 |

Number of observations: 72, Error degrees of freedom: 67
Root Mean Squared Error: 988
R-squared: 0.579, Adjusted R-Squared: 0.554
F-statistic vs. constant model: 23, p-value = 5.37e-12

## Analysis 4 Model A

Linear regression model (robust fit):
    DeathRate ~ 1 + Obesity + AgeOver65 + Age145

```
Estimated Coefficients:
                   Estimate      SE       tStat       pValue
                   ________    ______    _______    _________

    (Intercept)    -381.43     294.96    -1.2931      0.20216
    Obesity         8.9024     2.8938     3.0763    0.0034558
    AgeOver65       7390.4     1682.3     4.3931   6.1432e-05
    Age145          570.67     938.05    0.60835      0.54582


Number of observations: 52, Error degrees of freedom: 48
Root Mean Squared Error: 131
R-squared: 0.634,  Adjusted R-Squared: 0.611
F-statistic vs. constant model: 27.7, p-value = 1.52e-10
```

## Analysis 5 Model A

```
Linear regression model:
    DeathRate ~ 1 + Diabets + Obesity + Gini + GDP + AgeOver65

Estimated Coefficients:
                   Estimate       SE         tStat       pValue
                   ________    _________    _______    __________

    (Intercept)     -1799.8       856.93    -2.1003      0.037945
    Diabets         -68.348       19.638    -3.4804    0.00071452
    Obesity          51.848           10     5.1847    9.6933e-07
    Gini             2016.3       922.95     2.1846      0.031003
    GDP            -0.01311    0.0040848    -3.2094     0.0017357
    AgeOver65         14646       1977.8     7.4053    2.6185e-11


Number of observations: 118, Error degrees of freedom: 112
Root Mean Squared Error: 976
R-squared: 0.503,  Adjusted R-Squared: 0.481
F-statistic vs. constant model: 22.7, p-value = 1.15e-15
```

## Analysis 6 Model A

```
Linear regression model (robust fit):
    DeathRate ~ 1 + Obesity + Hyper + AgeOver65

Estimated Coefficients:
                   Estimate      SE       tStat       pValue
                   ________    ______    _______    _________
```

| | | | | |
|---|---|---|---|---|
| **(Intercept)** | -1807.1 | 387.11 | -4.6682 | 1.068e-05 |
| **Obesity** | 11.823 | 7.8225 | 1.5114 | 0.13424 |
| **Hyper** | 38.326 | 10.704 | 3.5806 | 0.00055797 |
| **AgeOver65** | 12433 | 1015.1 | 12.249 | 8.5173e-21 |

Number of observations: 93, Error degrees of freedom: 89
Root Mean Squared Error: 592
R-squared: 0.768, Adjusted R-Squared: 0.76
F-statistic vs. constant model: 98, p-value = 4.12e-28

## Analysis 7 Model A

Linear regression model:
DeathRate ~ 1 + Diabets + Obesity + Gini + Democracy + AgeOver65

Estimated Coefficients:

| | **Estimate** | **SE** | **tStat** | **pValue** |
|---|---|---|---|---|
| **(Intercept)** | -2352.2 | 571.63 | -4.1149 | 7.2057e-05 |
| **Diabets** | -45.474 | 15.484 | -2.9369 | 0.0039871 |
| **Obesity** | 35.311 | 7.6526 | 4.6143 | 1.0098e-05 |
| **Gini** | 1877.8 | 716.82 | 2.6196 | 0.0099588 |
| **Democracy** | 95.568 | 41.037 | 2.3288 | 0.021569 |
| **AgeOver65** | 18278 | 1887.5 | 9.6837 | 1.1276e-16 |

Number of observations: 124, Error degrees of freedom: 118
Root Mean Squared Error: 775
R-squared: 0.696, Adjusted R-Squared: 0.683
F-statistic vs. constant model: 54, p-value = 6.53e-29

## Analysis 8 Model A

Linear regression model:
DeathRate ~ 1 + Diabets + Obesity + AgeOver65

Estimated Coefficients:

| | **Estimate** | **SE** | **tStat** | **pValue** |
|---|---|---|---|---|
| **(Intercept)** | -111.2 | 93.253 | -1.1924 | 0.23485 |
| **Diabets** | -25.101 | 7.9895 | -3.1418 | 0.001999 |
| **Obesity** | 18.545 | 3.7699 | 4.9191 | 2.1304e-06 |
| **AgeOver65** | 3739.9 | 547.88 | 6.8261 | 1.6865e-10 |

```
Number of observations: 165, Error degrees of freedom: 161
Root Mean Squared Error: 460
R-squared: 0.409,  Adjusted R-Squared: 0.398
F-statistic vs. constant model: 37.2, p-value = 2.55e-18
```

### Analysis 9 Model A

```
Linear regression model (robust fit):
    DeathRate ~ 1 + Obesity + Democracy + GDP + GII + Hyper + MedianAge

Estimated Coefficients:
```

| | Estimate | SE | tStat | pValue |
|---|---|---|---|---|
| **(Intercept)** | -2478.5 | 303.95 | -8.1546 | 1.0191e-13 |
| **Obesity** | 14.934 | 3.9162 | 3.8133 | 0.00019619 |
| **Democracy** | 64.328 | 20.583 | 3.1253 | 0.0021142 |
| **GDP** | -0.011195 | 0.00211 | -5.3057 | 3.7375e-07 |
| **GII** | 2.7673 | 1.5088 | 1.8342 | 0.068512 |
| **Hyper** | 27.285 | 6.0353 | 4.521 | 1.2015e-05 |
| **MedianAge** | 50.523 | 7.1639 | 7.0524 | 5.1815e-11 |

```
Number of observations: 165, Error degrees of freedom: 158
Root Mean Squared Error: 448
R-squared: 0.616,  Adjusted R-Squared: 0.601
F-statistic vs. constant model: 42.3, p-value = 1.79e-30
```

### Analysis 10 Model A

```
Linear regression model:
    DeathRate ~ 1 + Diabets + Gini + GDP + AgeOver65

Estimated Coefficients:
```

| | Estimate | SE | tStat | pValue |
|---|---|---|---|---|
| **(Intercept)** | -296.61 | 103.6 | -2.863 | 0.0047594 |
| **Diabets** | -5.5102 | 2.551 | -2.16 | 0.032264 |
| **Gini** | 335.19 | 125.98 | 2.6606 | 0.0085945 |
| **GDP** | 0.0023013 | 0.00064707 | 3.5565 | 0.00049468 |
| **AgeOver65** | 2473.5 | 255.21 | 9.6919 | 9.1793e-18 |

```
Number of observations: 165, Error degrees of freedom: 160
Root Mean Squared Error: 159
R-squared: 0.582,  Adjusted R-Squared: 0.572
F-statistic vs. constant model: 55.7, p-value = 2.26e-29
```
