# Supplementary material for: An Ecological Study on the Mortality Impact of the COVID-19 Pandemic According to Country Development Status and Pandemic Years
Source: Epidemiologia (Basel). 2026 Apr 6;7(2):50. doi: 10.3390/epidemiologia7020050 (PMC13115391; doi:10.3390/epidemiologia7020050)
Supplement: Supplementary file 1 [file epidemiologia-07-00050-s001.zip › Supplementary material - AMR Regression Results and Diagnostics.pdf]

AMR Regression Results and Diagnostics

Tabla de contenido

Analysis 1 2
Analysis 1 Model with interactions 5
Analysis 2 9
Analysis 3 12
Analysis 4 16
Analysis 5 19
Analysis 6 22
Analysis 7 24
Analysis 8 28
Analysis 9 32
Analysis 10 36

Analysis 1

```
Linear regression model (robust fit):
    DeathRate ~ 1 + Diabetes + Obesity + Gini + GDP + Hyper + AgeOver65
```

Estimated Coefficients:

	Estimate	SE	tStat	pValue
**(Intercept)**	-2914.3	561.32	-5.1919	5.9911e-07
**Diabetes**	-29.311	11.954	-2.452	0.015234
**Obesity**	30.361	6.2483	4.8591	2.7002e-06
**Gini**	1758.4	528.9	3.3246	0.0010884
**GDP**	-0.0067009	0.0030971	-2.1636	0.031916
**Hyper**	31.45	9.2372	3.4047	0.0008294
**AgeOver65**	15101	1137.7	13.274	6.4715e-28

```
Number of observations: 174, Error degrees of freedom: 167
Root Mean Squared Error: 693
R-squared: 0.702,  Adjusted R-Squared: 0.691
F-statistic vs. constant model: 65.5, p-value = 2.47e-41
```

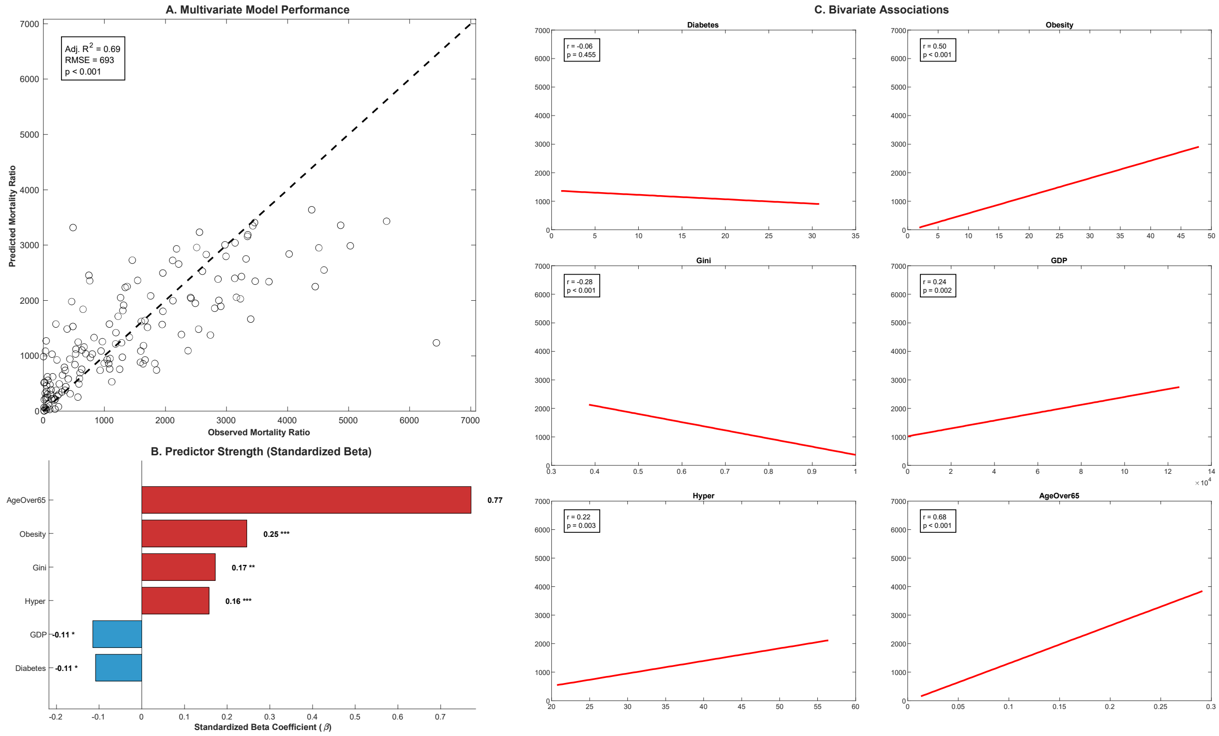

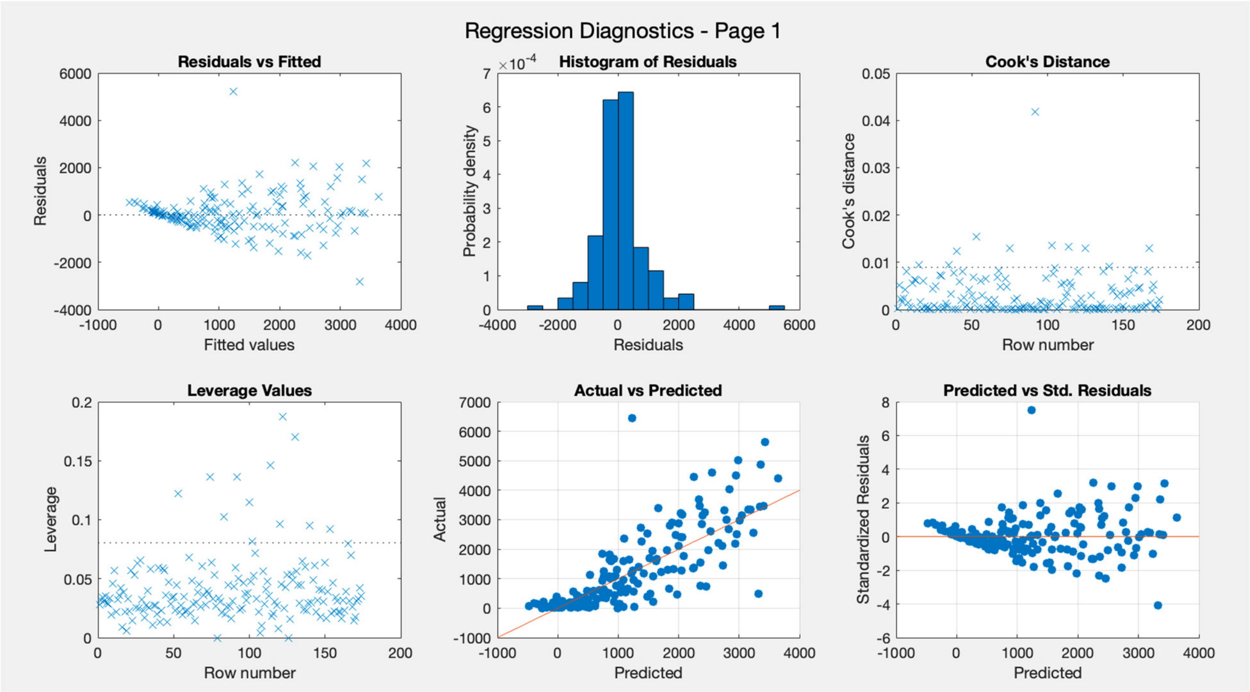
Regression Diagnostics - Page 1
Residuals vs Fitted
Residuals
Fitted values
Histogram of Residuals
Probability density
×10^-4
Residuals
Cook's Distance
Cook's distance
Row number
Leverage Values
Leverage
Row number
Actual vs Predicted
Actual
Predicted
Predicted vs Std. Residuals
Standardized Residuals
Predicted

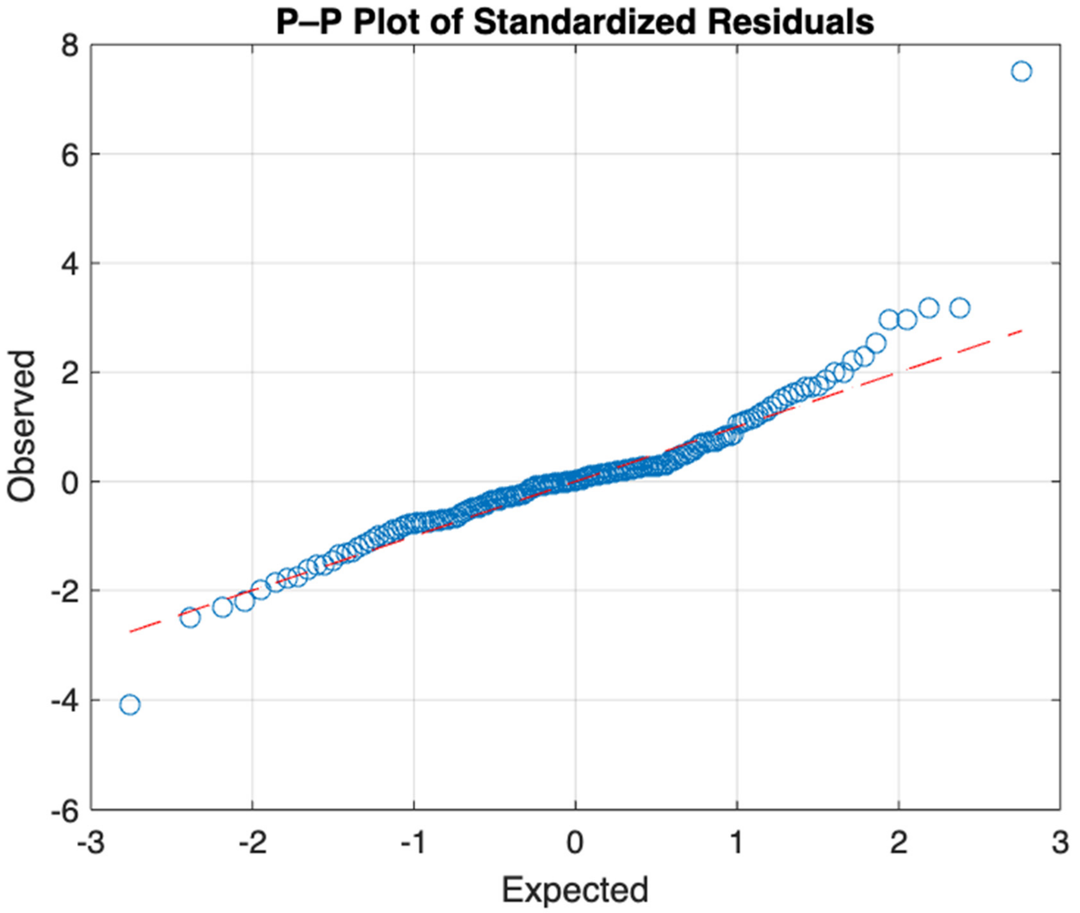
P–P Plot of Standardized Residuals
Observed
Expected

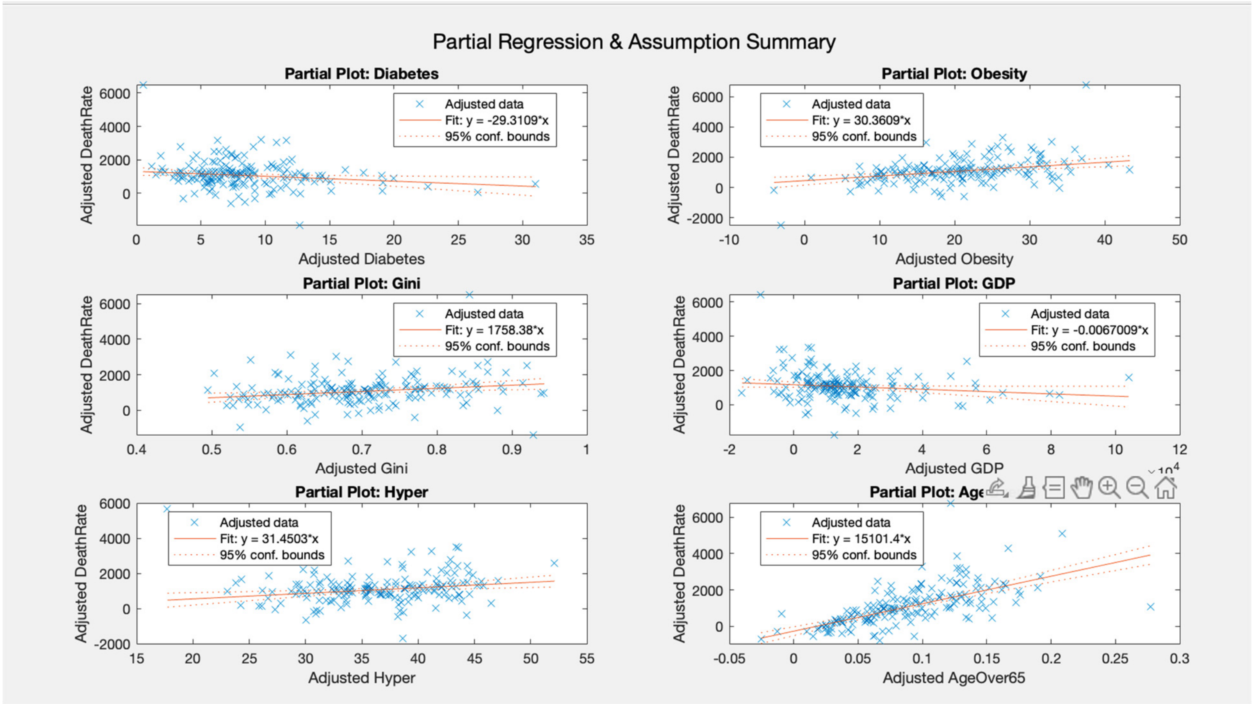

Model Assumption Tests Summary

Durbin-Watson Statistic: 1.861

Breusch–Pagan LM Stat: 18.792 | p-value: 0.0045 → Violated

White Test Stat: 93.068 | p-value: 0.0000 → Violated

Variance Inflation Factor (VIF) Values:

Predictor	VIF
Diabetes	1.30
Obesity	1.71
Gini	1.79
GDP	1.88
Hyper	1.43
AgeOver65	2.26

Analysis 1 Model with interactions

```
Linear regression model:
    DeathRate ~ 1 + Obesity + Diabets*Hyper + Diabets*Unemployment +
Cardio*GDP + Cardio*Hyper + Cardio*LifeExpectancy + Democracy*GDP +
Democracy*GII + Democracy*MedianAge + Democracy*AgeOver65 + Density*IHDI +
Density*Hyper + Density*AgeOver65 + GDP*AgeOver65 + GII*AgeOver65 +
Hyper*LifeExpectancy + Hyper*AgeOver65 + MedianAge*Unemployment

Estimated Coefficients:
```

	Estimate	SE	tStat	pValue
**(Intercept)**	-10985	6746	-1.6283	0.10565
**Diabetes**	-161.72	76.05	-2.1266	0.035173
**Obesity**	28.535	8.0008	3.5665	0.00049261
**Cardio**	884.24	20810	0.04249	0.96617
**Democracy**	-742.3	255.81	-2.9018	0.0042983
**Density**	4.6802	1.8199	2.5716	0.011142
**GDP**	0.055167	0.019044	2.8969	0.0043619
**IHDI**	14.82	13.314	1.1132	0.26751
**GII**	-18.962	6.8897	-2.7522	0.006687
**Hyper**	421.24	163.49	2.5764	0.010995
**LifeExpectancy**	223.72	99.959	2.2381	0.026761
**MedianAge**	-225.7	63.622	-3.5474	0.00052655
**AgeOver65**	-13800	15027	-0.91835	0.35998
**Unemployment**	-93.448	37.366	-2.5009	0.013516
**Diabets:Hyper**	4.6559	2.0388	2.2836	0.023869
**Diabets:Unemployment**	-6.2742	2.6709	-2.349	0.02019
**Cardio:GDP**	-0.19848	0.090271	-2.1988	0.029501
**Cardio:Hyper**	-859.46	254.79	-3.3732	0.0009561
**Cardio:LifeExpectancy**	506.55	263.62	1.9215	0.056653

**Democracy:GDP**	-0.0094265	0.003012	-3.1297	0.0021217
**Democracy:GII**	2.7017	1.3696	1.9725	0.050476
**Democracy:MedianAge**	39.052	10.673	3.6591	0.00035521
**Democracy:AgeOver65**	-4386	1394.2	-3.146	0.0020144
**Density:IHDI**	-0.071291	0.034769	-2.0504	0.042149
**Density:Hyper**	-0.083021	0.030698	-2.7044	0.0076733
**Density:AgeOver65**	-11.235	5.0246	-2.2359	0.026908
**GDP:AgeOver65**	0.26491	0.11315	2.3413	0.020599
**GII:AgeOver65**	192.26	69.61	2.7619	0.0065016
**Hyper:LifeExpectancy**	-6.5293	2.4002	-2.7203	0.0073311
**Hyper:AgeOver65**	1095.5	239.31	4.5776	1.0128e-05
**MedianAge:Unemployment**	6.7259	1.4182	4.7428	5.0488e-06

```
Number of observations: 174, Error degrees of freedom: 143
Root Mean Squared Error: 672
R-squared: 0.798,  Adjusted R-Squared: 0.756
F-statistic vs. constant model: 18.9, p-value = 3.39e-36
```

- In Modeling Approach B (Interaction-Inclusive), specific granular age cohorts (0–4, 5–14, 15–24, 25–64), HDI and the prevalence of Lung Diseases were manually excluded from the candidate pool to prevent a rank-deficient design matrix (which yielded NaN statistics in preliminary runs). In contrast, while the retained aggregate demographic predictors—specifically Median Age (VIF=21.94) and Age >65 (VIF=15.38)—exhibited elevated VIF values, they were preserved in the final model. These elevations are attributable to 'structural multicollinearity', a natural artifact of including interaction terms involving these main effects. Removing them would violate the principle of marginality (hierarchy), rendering the significant interaction terms uninterpretable; thus, their inclusion is methodologically required..

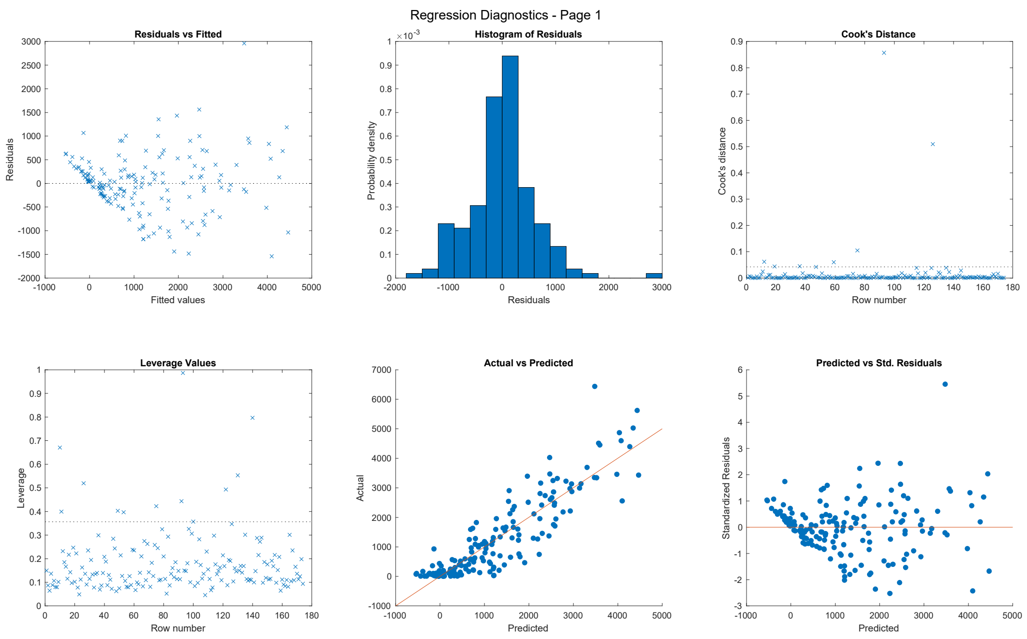
Regression Diagnostics - Page 1
Residuals vs Fitted
Residuals
Fitted values
Histogram of Residuals
Probability density
Residuals
Cook's Distance
Cook's distance
Row number
Leverage Values
Leverage
Row number
Actual vs Predicted
Actual
Predicted
Predicted vs Std. Residuals
Standardized Residuals
Predicted

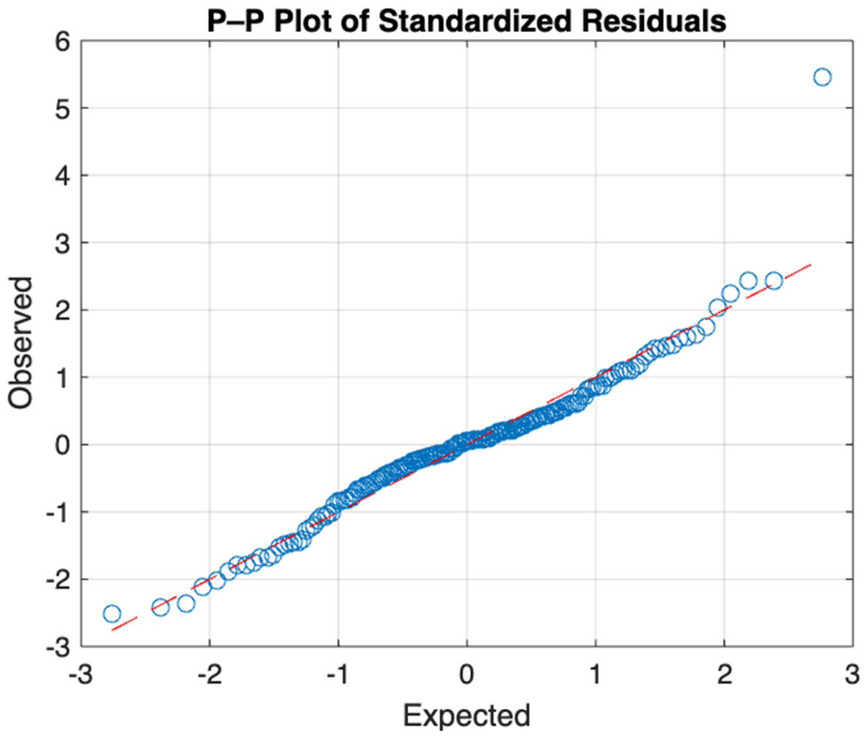
P–P Plot of Standardized Residuals
Observed
Expected

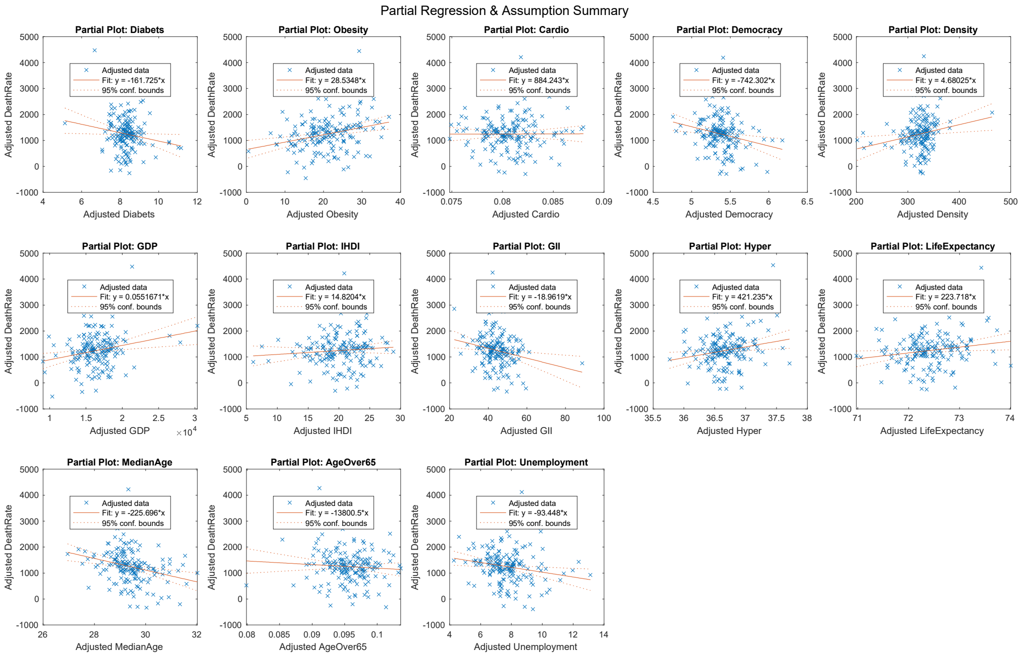

Model Assumption Tests Summary

Durbin-Watson Statistic: 1.757

Breusch–Pagan LM Stat: 22.919 | p-value: 0.0427 → Violated

White Test Stat: 137.685 | p-value: 0.0151 → Violated

Variance Inflation Factor (VIF) Values:

Predictor	VIF
Diabets	1.46
Obesity	2.35
Cardio	1.11
Democracy	2.21
Density	1.15
GDP	2.27
IHDI	5.95
GII	4.71
Hyper	1.63
LifeExpectancy	6.49
MedianAge	21.46
AgeOver65	13.63
Unemployment	1.29

Analysis 2

Linear regression model:
    DeathRate ~ 1 + Hyper + LifeExpectancy + LungDiseases + Unemployment

Estimated Coefficients:

	Estimate	SE	tStat	pValue
**(Intercept)**	12058	6165.4	1.9558	0.058289
**Hyper**	84.161	23.919	3.5186	0.0011951
**LifeExpectancy**	-184.6	70.213	-2.6291	0.01251
**LungDiseases**	14887	4005.1	3.717	0.00068239
**Unemployment**	124.52	40.238	3.0945	0.0038015

Number of observations: 41, Error degrees of freedom: 36
Root Mean Squared Error: 722
R-squared: 0.627, Adjusted R-Squared: 0.586
F-statistic vs. constant model: 15.1, p-value = 2.39e-07

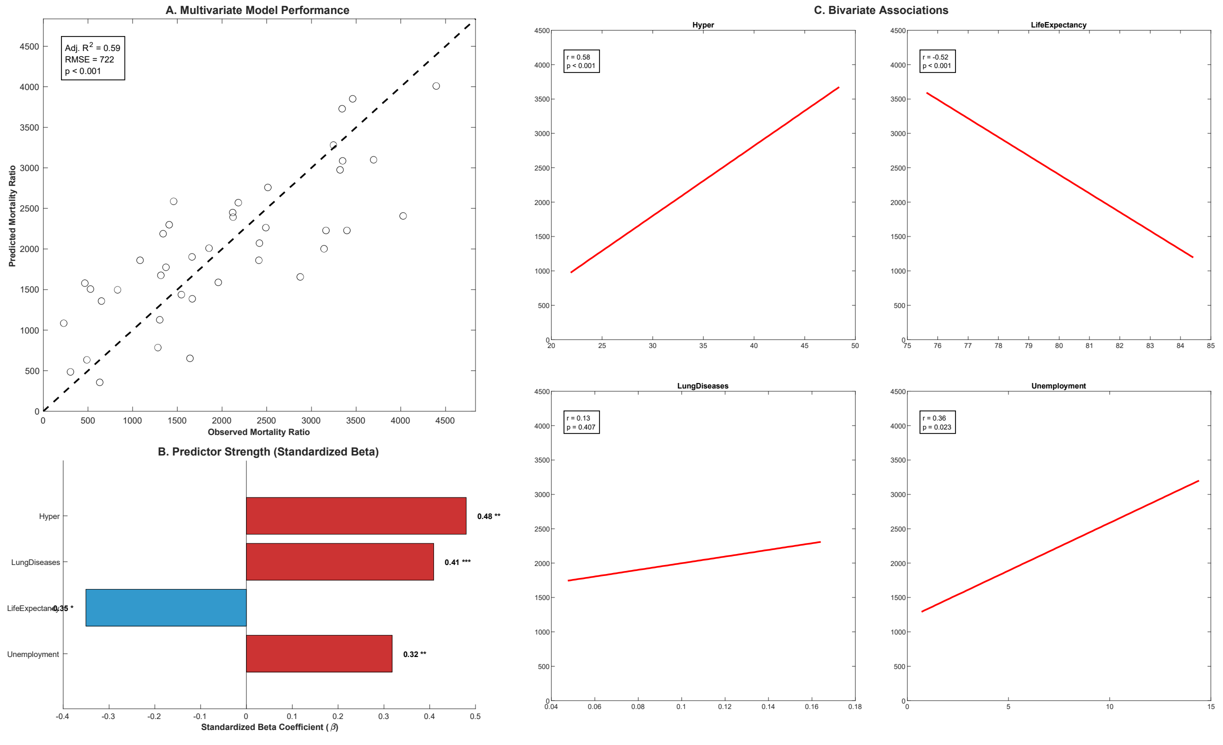

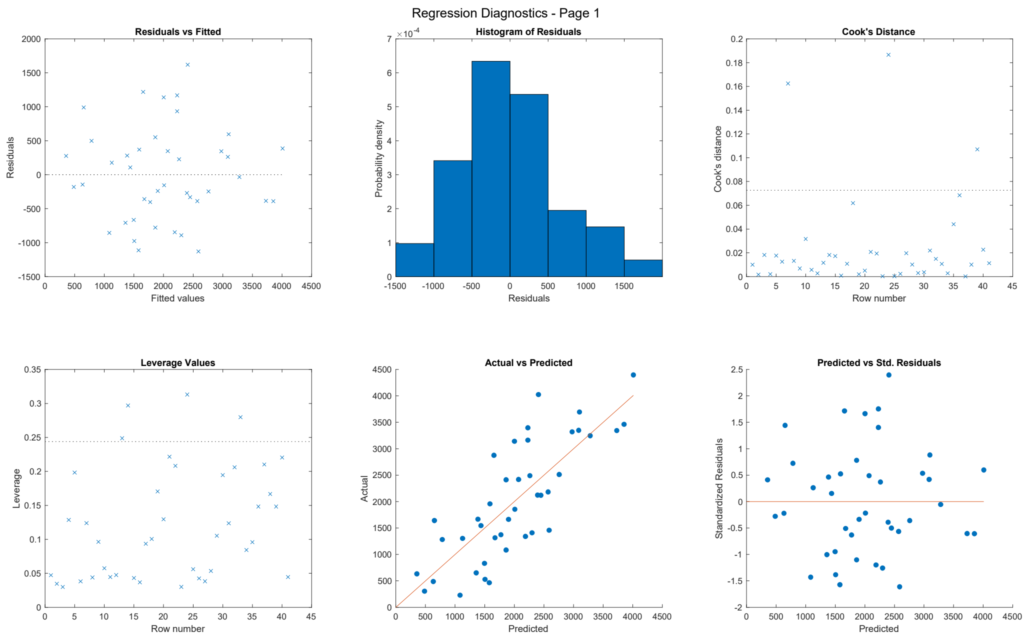
Regression Diagnostics - Page 1
Residuals vs Fitted
Residuals
Fitted values
Histogram of Residuals
Probability density
Residuals
Cook's Distance
Cook's distance
Row number
Leverage Values
Leverage
Row number
Actual vs Predicted
Actual
Predicted
Predicted vs Std. Residuals
Standardized Residuals
Predicted

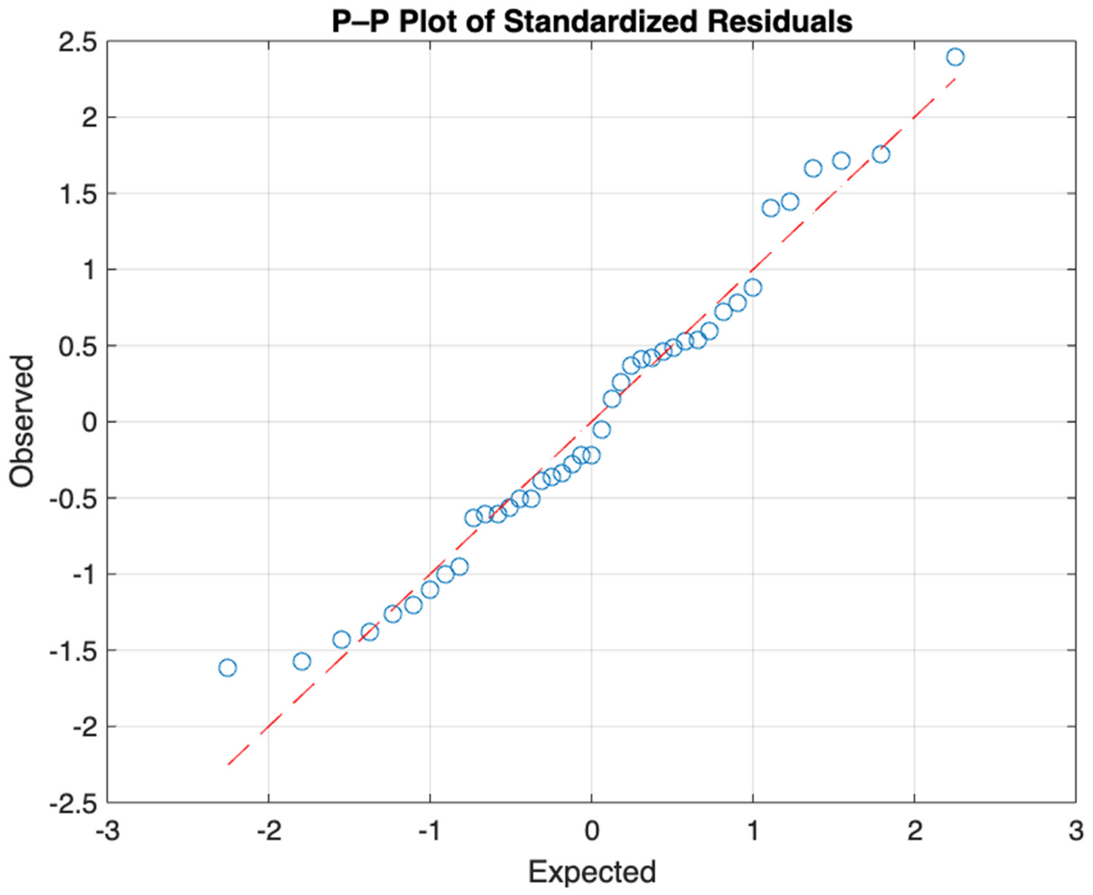
P–P Plot of Standardized Residuals
Observed
Expected

Partial Regression & Assumption Summary

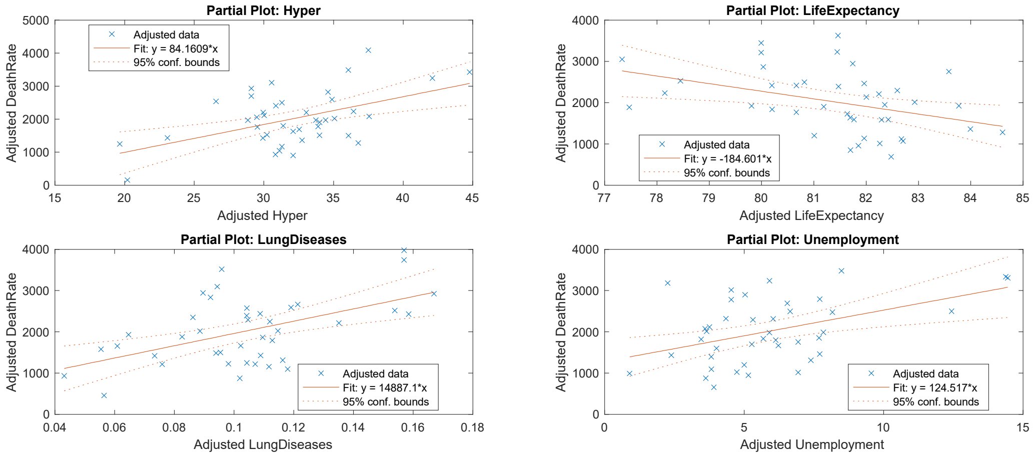

Model Assumption Tests Summary

Durbin-Watson Statistic: 1.974

Breusch–Pagan LM Stat: 1.778 | p-value: 0.7765 → Satisfied

White Test Stat: 16.769 | p-value: 0.2687 → Satisfied

Variance Inflation Factor (VIF) Values:

Predictor	VIF
Hyper	1.79
LifeExpectancy	1.71
LungDiseases	1.17
Unemployment	1.02

Analysis 3

Linear regression model:
    DeathRate ~ 1 + Diabets + Obesity + Gini + AgeOver65

Estimated Coefficients:

	Estimate	SE	tStat	pValue
**(Intercept)**	-2890.6	1064.5	-2.7155	0.0082782
**Diabets**	-61.061	24.025	-2.5416	0.013188
**Obesity**	43.878	12.906	3.3998	0.0011031
**Gini**	2915.3	1130.6	2.5786	0.011964
**AgeOver65**	20158	2712.9	7.4305	1.7646e-10

Number of observations: 77, Error degrees of freedom: 72
Root Mean Squared Error: 977
R-squared: 0.571, Adjusted R-Squared: 0.547
F-statistic vs. constant model: 24, p-value = 1.27e-12

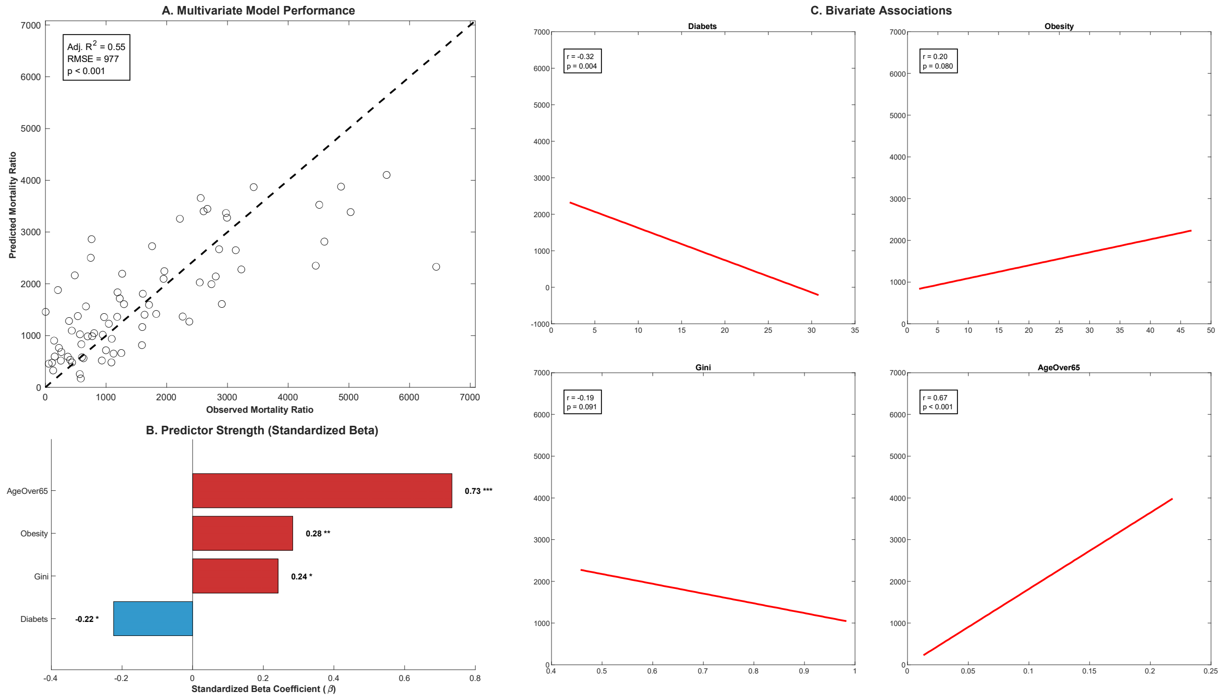

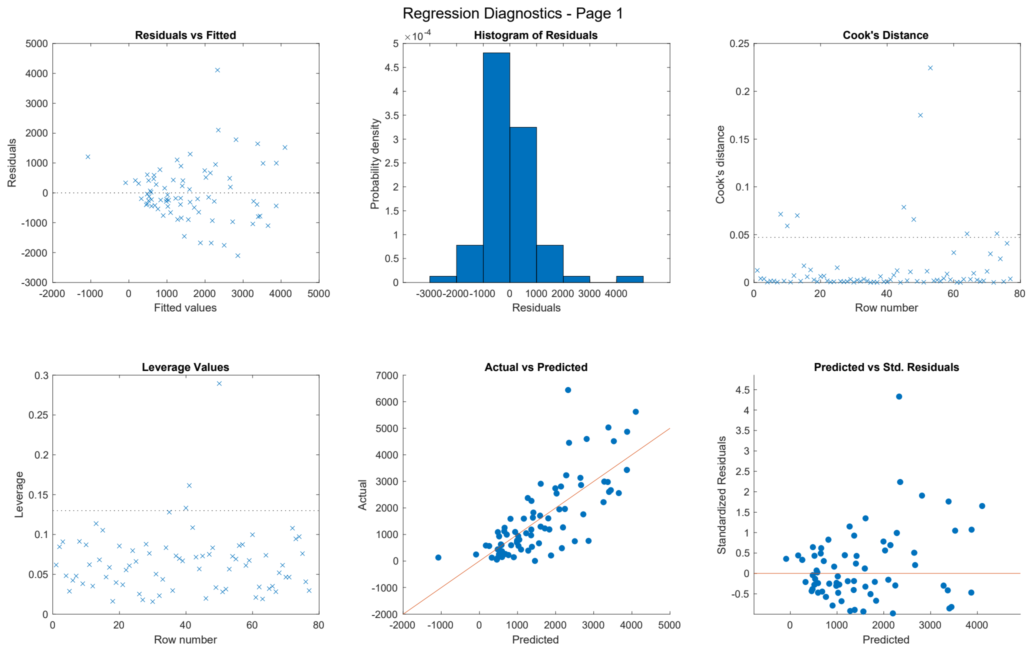
Regression Diagnostics - Page 1
Residuals vs Fitted
Residuals
Fitted values
Histogram of Residuals
Probability density
Residuals
Cook's Distance
Cook's distance
Row number
Leverage Values
Leverage
Row number
Actual vs Predicted
Actual
Predicted
Predicted vs Std. Residuals
Standardized Residuals
Predicted

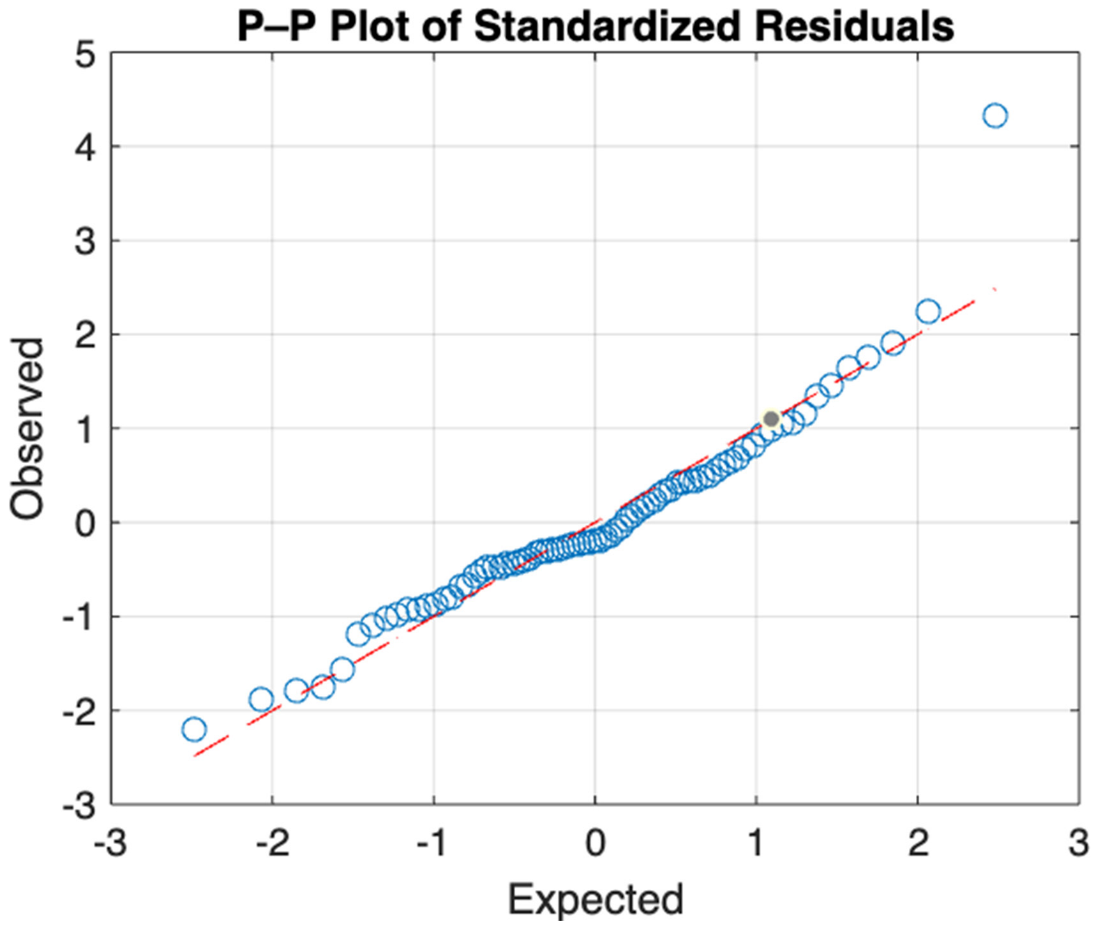
P–P Plot of Standardized Residuals
Observed
Expected

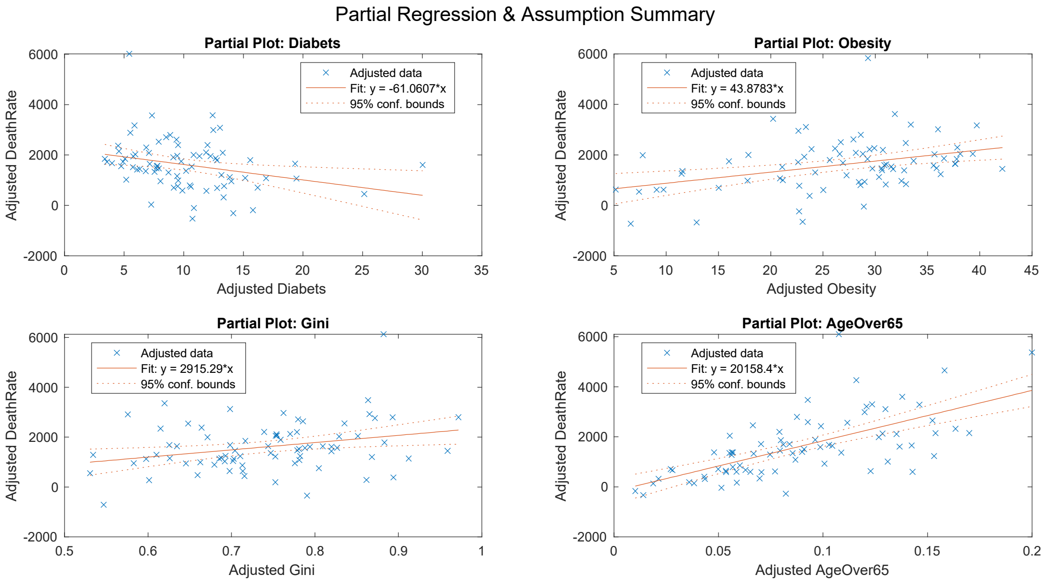
Partial Regression & Assumption Summary
Partial Plot: Diabets
Adjusted data
Fit: y = -61.0607*x
95% conf. bounds
Adjusted DeathRate
Adjusted Diabets
Partial Plot: Obesity
Adjusted data
Fit: y = 43.8783*x
95% conf. bounds
Adjusted DeathRate
Adjusted Obesity
Partial Plot: Gini
Adjusted data
Fit: y = 2915.29*x
95% conf. bounds
Adjusted DeathRate
Adjusted Gini
Partial Plot: AgeOver65
Adjusted data
Fit: y = 20158.4*x
95% conf. bounds
Adjusted DeathRate
Adjusted AgeOver65

Model Assumption Tests Summary

Durbin-Watson Statistic: 1.659

Breusch–Pagan LM Stat: 6.786 | p-value: 0.1476 → Satisfied

White Test Stat: 23.101 | p-value: 0.0587 → Satisfied

Variance Inflation Factor (VIF) Values:

Predictor	VIF
Diabets	1.30
Obesity	1.17
Gini	1.48
AgeOver65	1.64

Analysis 4

Linear regression model (robust fit):
    DeathRate ~ 1 + Obesity + AgeOver65 + Age145

Estimated Coefficients:

	**Estimate**	**SE**	**tStat**	**pValue**
**(Intercept)**	-505.67	279.81	-1.8072	0.07652
**Obesity**	10.051	2.71	3.7089	0.00050687
**AgeOver65**	8503.6	1612.2	5.2745	2.6175e-06
**Age145**	877.52	885.7	0.99076	0.32639

Number of observations: 56, Error degrees of freedom: 52
Root Mean Squared Error: 126
R-squared: 0.671, Adjusted R-Squared: 0.652
F-statistic vs. constant model: 35.4, p-value = 1.32e-12

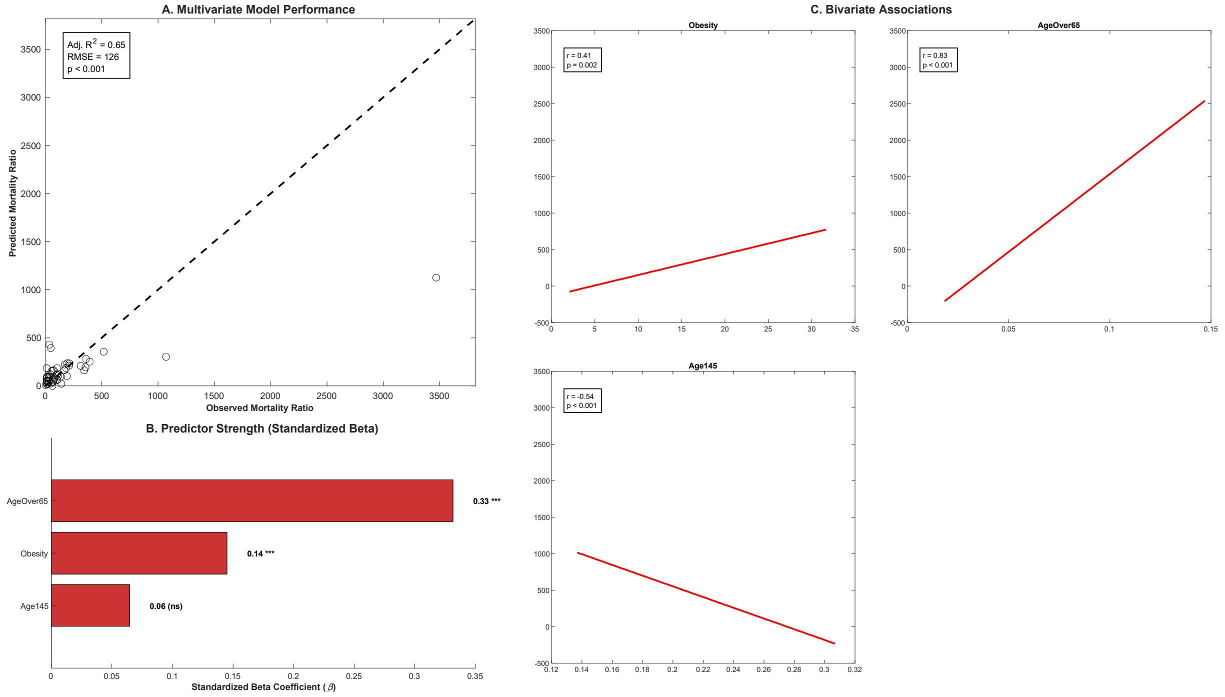

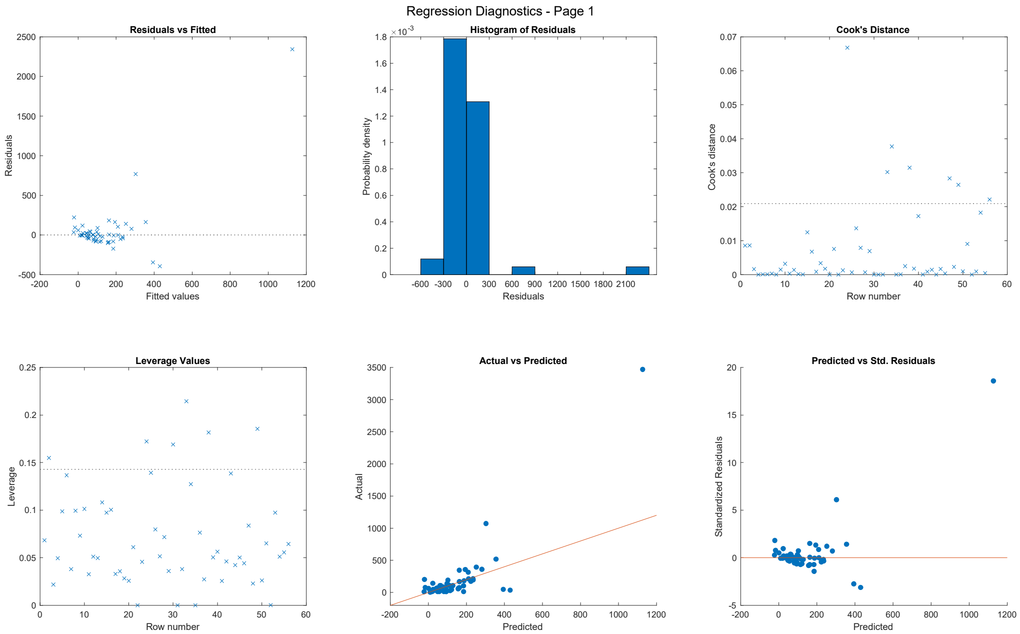
Regression Diagnostics - Page 1
Residuals vs Fitted
Residuals
Fitted values
Histogram of Residuals
Probability density
Residuals
Cook's Distance
Cook's distance
Row number
Leverage Values
Leverage
Row number
Actual vs Predicted
Actual
Predicted
Predicted vs Std. Residuals
Standardized Residuals
Predicted

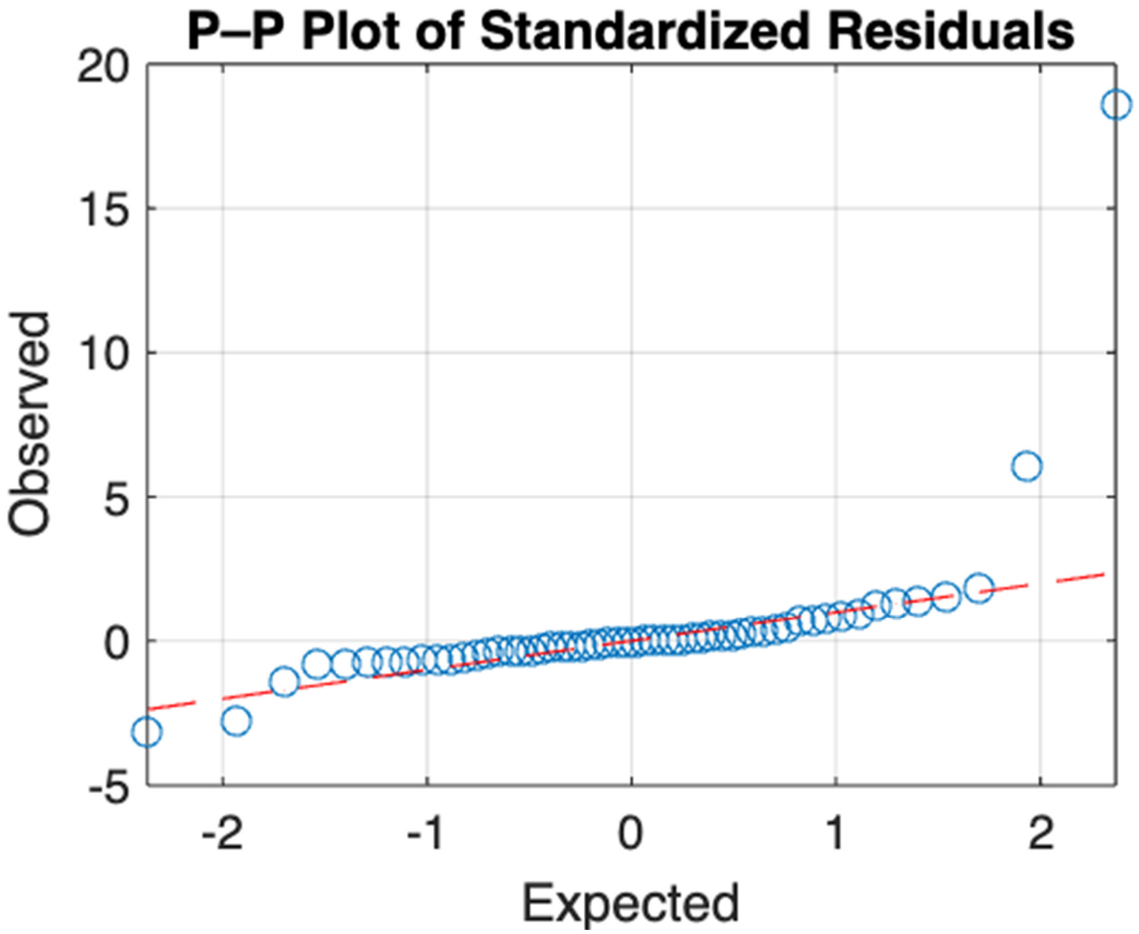
P–P Plot of Standardized Residuals
Observed
Expected

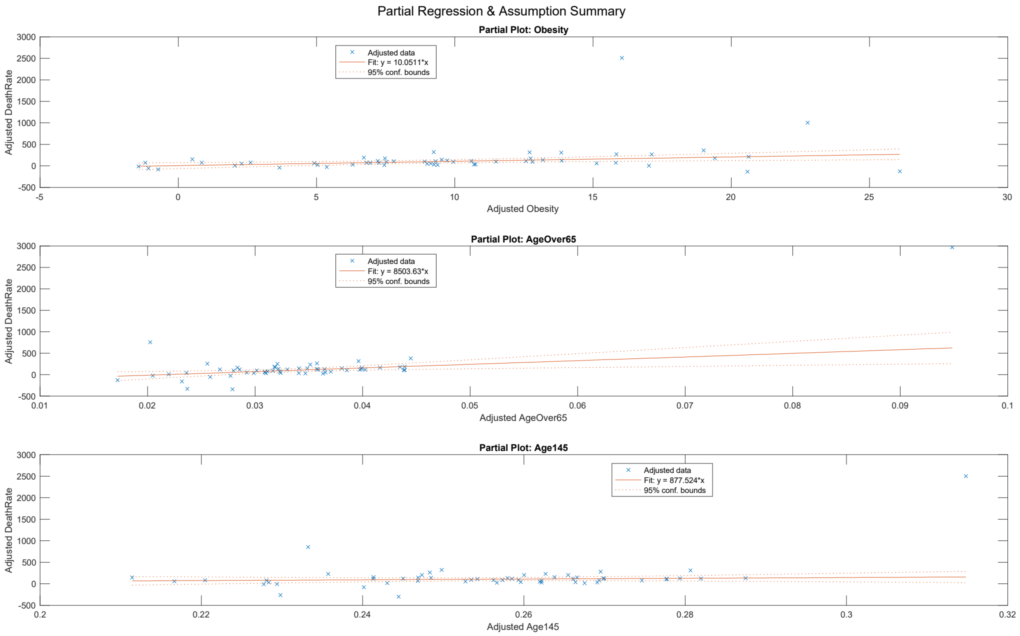

Model Assumption Tests Summary

Durbin-Watson Statistic: 2.018

Breusch–Pagan LM Stat: 45.900 | p-value: 0.0000 → Violated

White Test Stat: 55.575 | p-value: 0.0000 → Violated

Variance Inflation Factor (VIF) Values:

Predictor	VIF
Obesity	1.21
AgeOver65	3.13
Age145	3.36

Analysis 5

Linear regression model:
DeathRate ~ 1 + Diabets + Obesity + Gini + GDP + AgeOver65

Estimated Coefficients:

	Estimate	SE	tStat	pValue
**(Intercept)**	-1799.8	856.93	-2.1003	0.037945
**Diabets**	-68.348	19.638	-3.4804	0.00071452
**Obesity**	51.848	10	5.1847	9.6933e-07
**Gini**	2016.3	922.95	2.1846	0.031003
**GDP**	-0.01311	0.0040848	-3.2094	0.0017357
**AgeOver65**	14646	1977.8	7.4053	2.6185e-11

Number of observations: 118, Error degrees of freedom: 112
Root Mean Squared Error: 976
R-squared: 0.503, Adjusted R-Squared: 0.481
F-statistic vs. constant model: 22.7, p-value = 1.15e-15

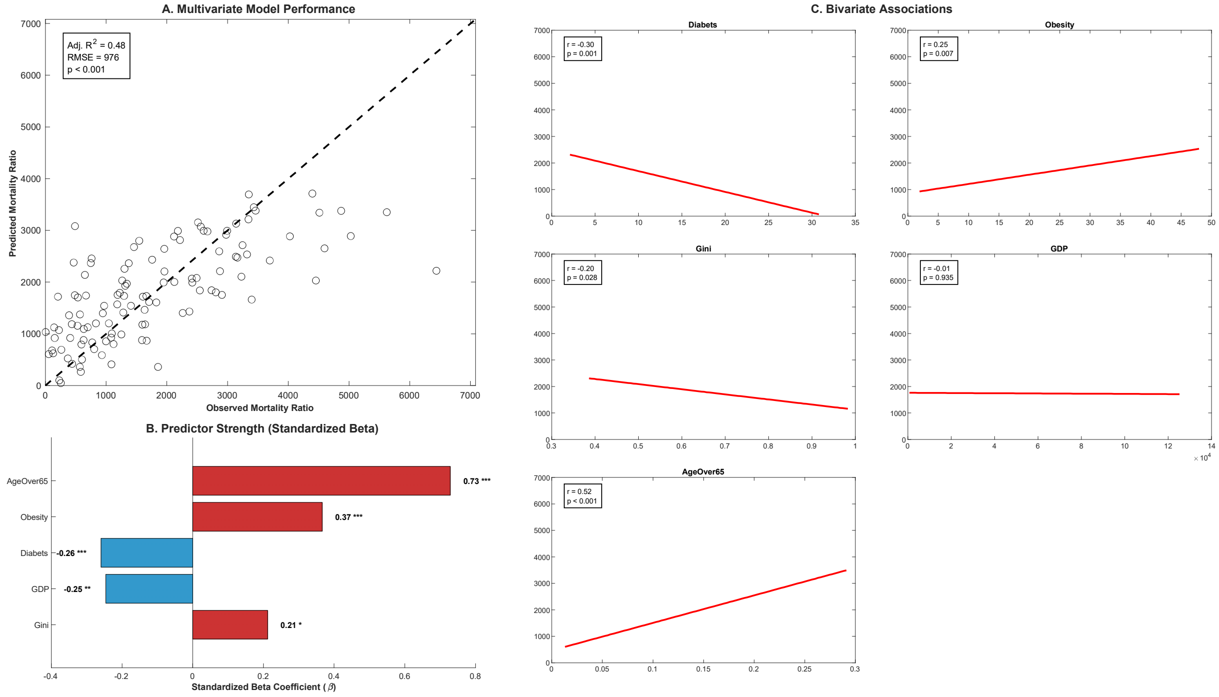

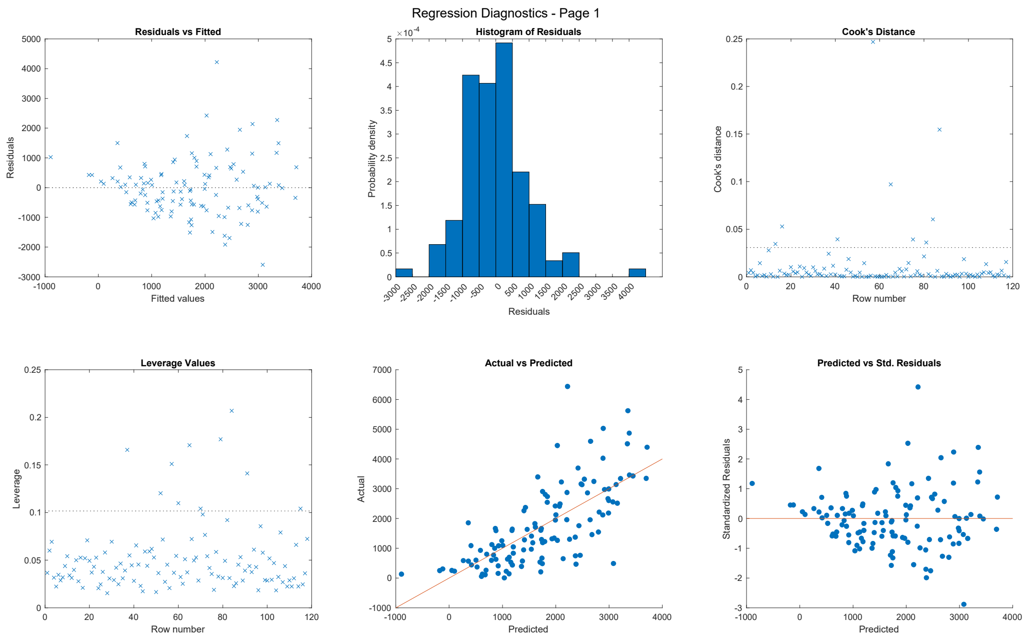
Regression Diagnostics - Page 1
Residuals vs Fitted
Residuals
Fitted values
Histogram of Residuals
Probability density
Residuals
Cook's Distance
Cook's distance
Row number
Leverage Values
Leverage
Row number
Actual vs Predicted
Actual
Predicted
Predicted vs Std. Residuals
Standardized Residuals
Predicted

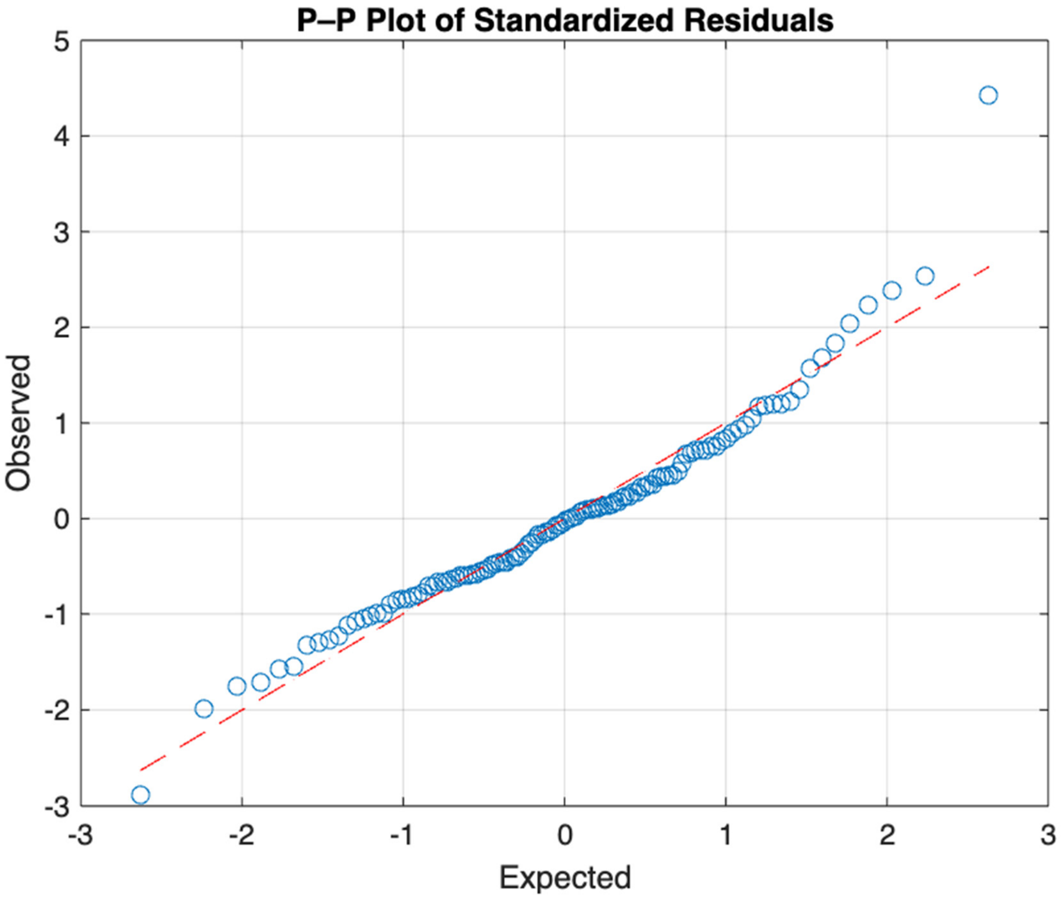
P–P Plot of Standardized Residuals
Observed
Expected

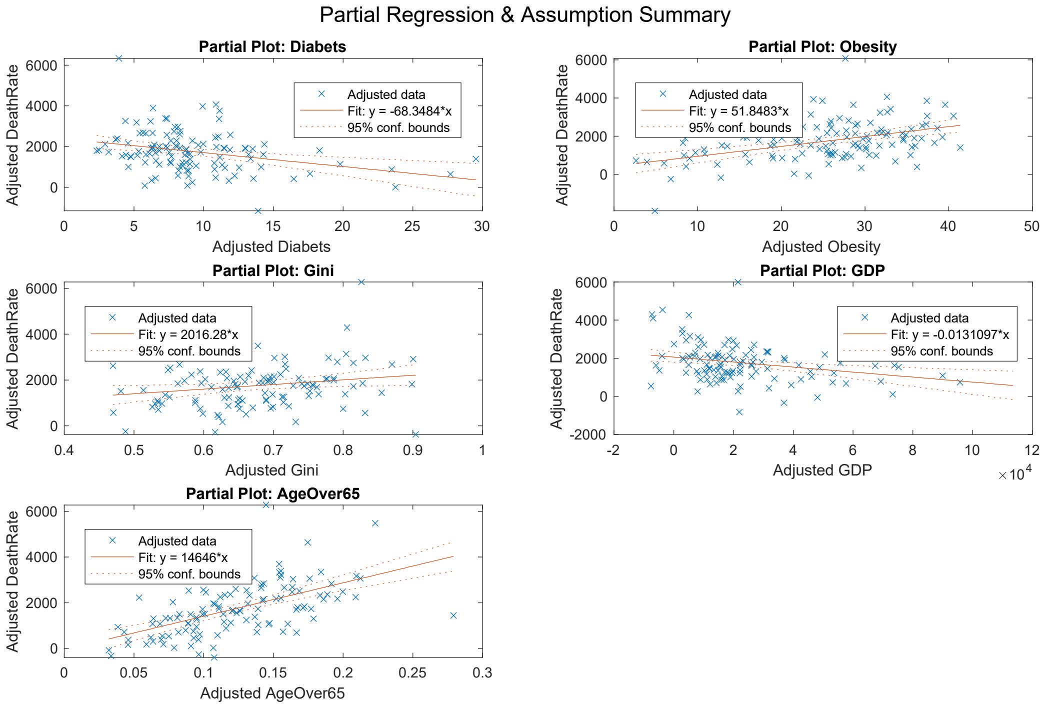

Model Assumption Tests Summary

Durbin-Watson Statistic: 1.986

Breusch–Pagan LM Stat: 7.059 | p-value: 0.2163 → Satisfied

White Test Stat: 31.216 | p-value: 0.0524 → Satisfied

Variance Inflation Factor (VIF) Values:

Predictor	VIF
Diabets	1.25
Obesity	1.13
Gini	2.12
GDP	1.32
AgeOver65	2.18

Analysis 6

Linear regression model (robust fit):
    DeathRate ~ 1 + Obesity + Hyper + AgeOver65

Estimated Coefficients:

	Estimate	SE	tStat	pValue
**(Intercept)**	-296.24	216.13	-1.3706	0.17379
**Obesity**	15.582	4.3998	3.5416	0.00062379
**Hyper**	0.413	5.9623	0.069269	0.94492
**AgeOver65**	6781.5	565.89	11.984	1.4591e-20

Number of observations: 97, Error degrees of freedom: 93
Root Mean Squared Error: 337
R-squared: 0.824, Adjusted R-Squared: 0.818
F-statistic vs. constant model: 145, p-value = 6.58e-35

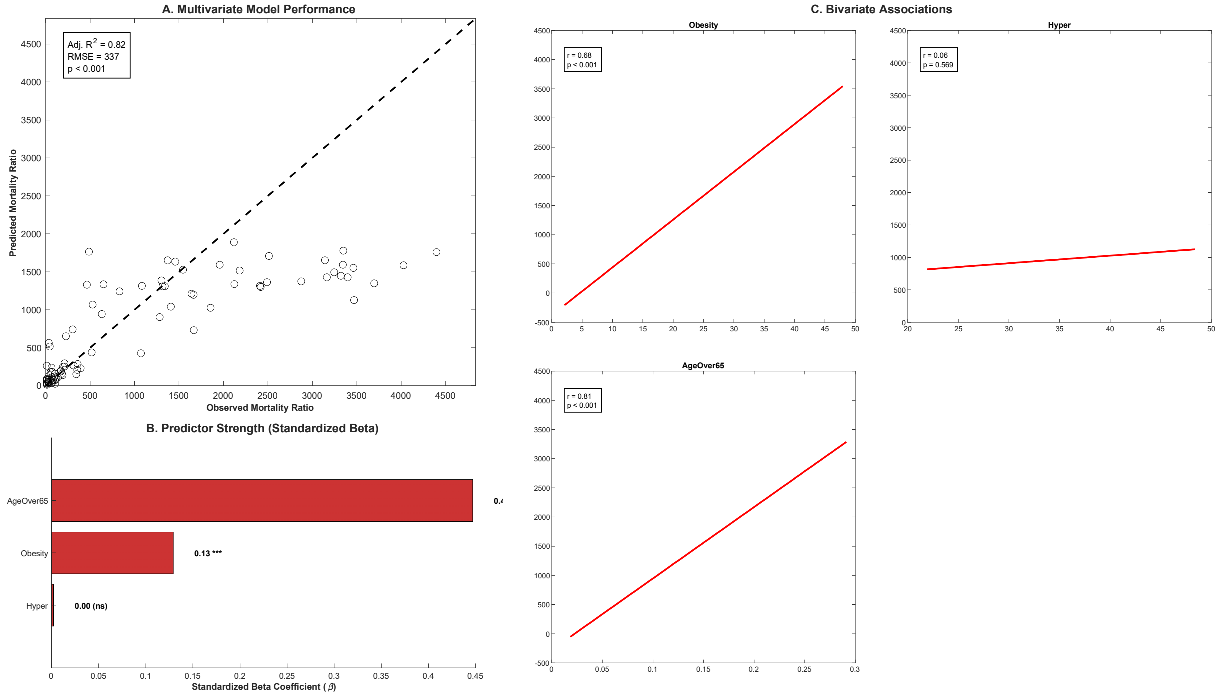

Regression Diagnostics - Page 1

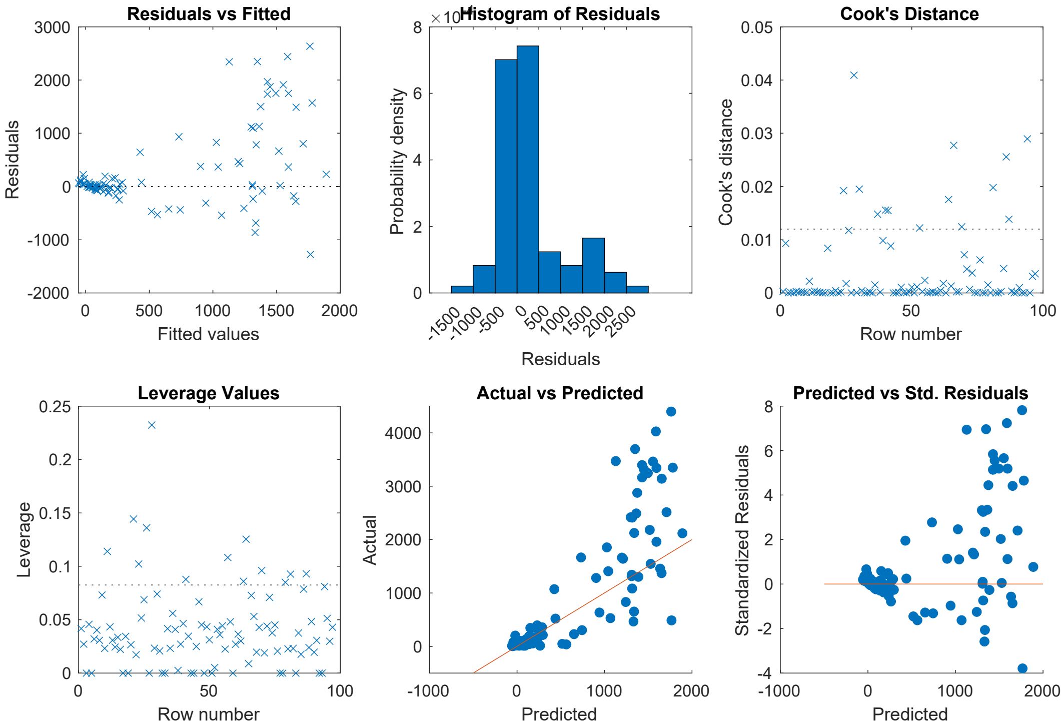

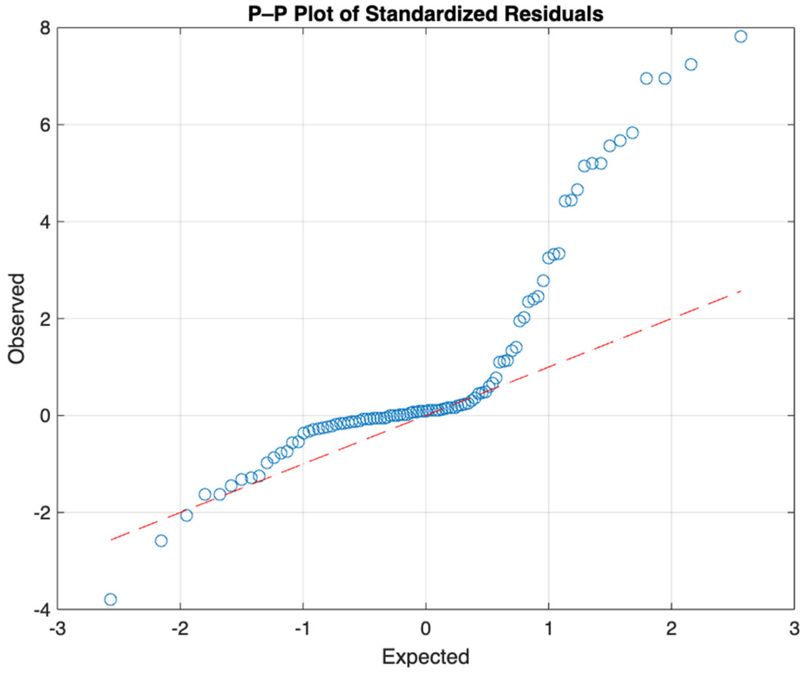

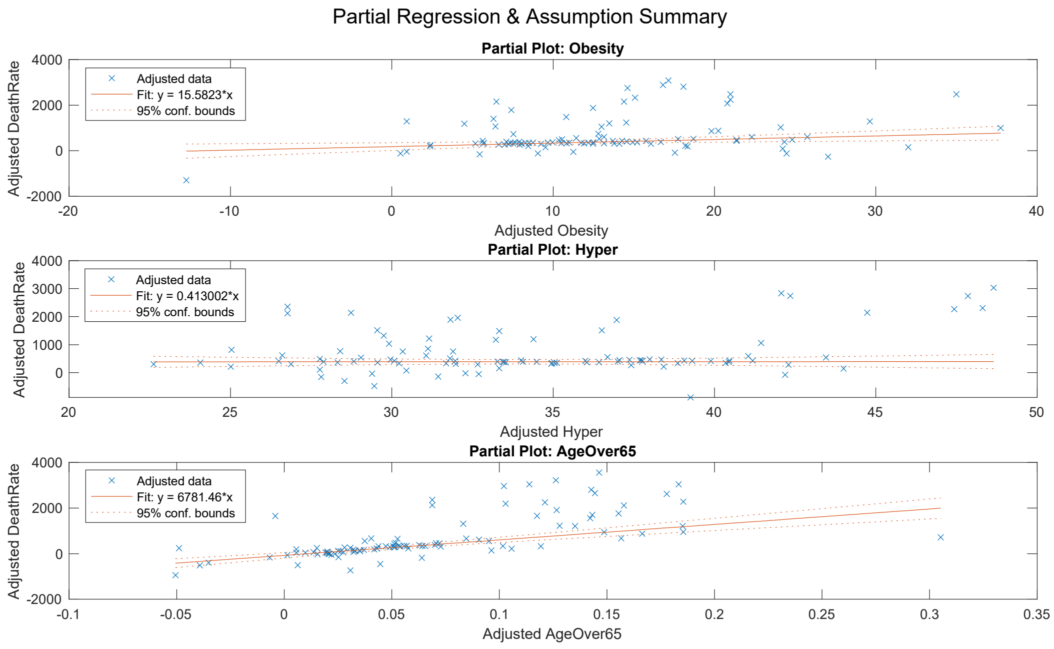

Model Assumption Tests Summary

Durbin-Watson Statistic: 1.705

Breusch–Pagan LM Stat: 46.425 | p-value: 0.0000 → Violated

White Test Stat: 63.675 | p-value: 0.0000 → Violated

Variance Inflation Factor (VIF) Values:

Predictor	VIF
Obesity	1.66
Hyper	1.12
AgeOver65	1.74

Analysis 7

```
Linear regression model:
    DeathRate ~ 1 + Diabets + Obesity + Gini + Democracy + AgeOver65
```

Estimated Coefficients:

	Estimate	SE	tStat	pValue
**(Intercept)**	-2166.9	532.78	-4.0672	8.2976e-05
**Diabets**	-45.391	14.654	-3.0975	0.0024025
**Obesity**	34.091	7.3584	4.6329	8.8046e-06
**Gini**	1675	654.94	2.5574	0.01172
**Democracy**	94.63	37.743	2.5072	0.013431
**AgeOver65**	18048	1753.4	10.293	1.9317e-18

Number of observations: 133, Error degrees of freedom: 127
Root Mean Squared Error: 760
R-squared: 0.692, Adjusted R-Squared: 0.68
F-statistic vs. constant model: 57.1, p-value = 7.23e-31

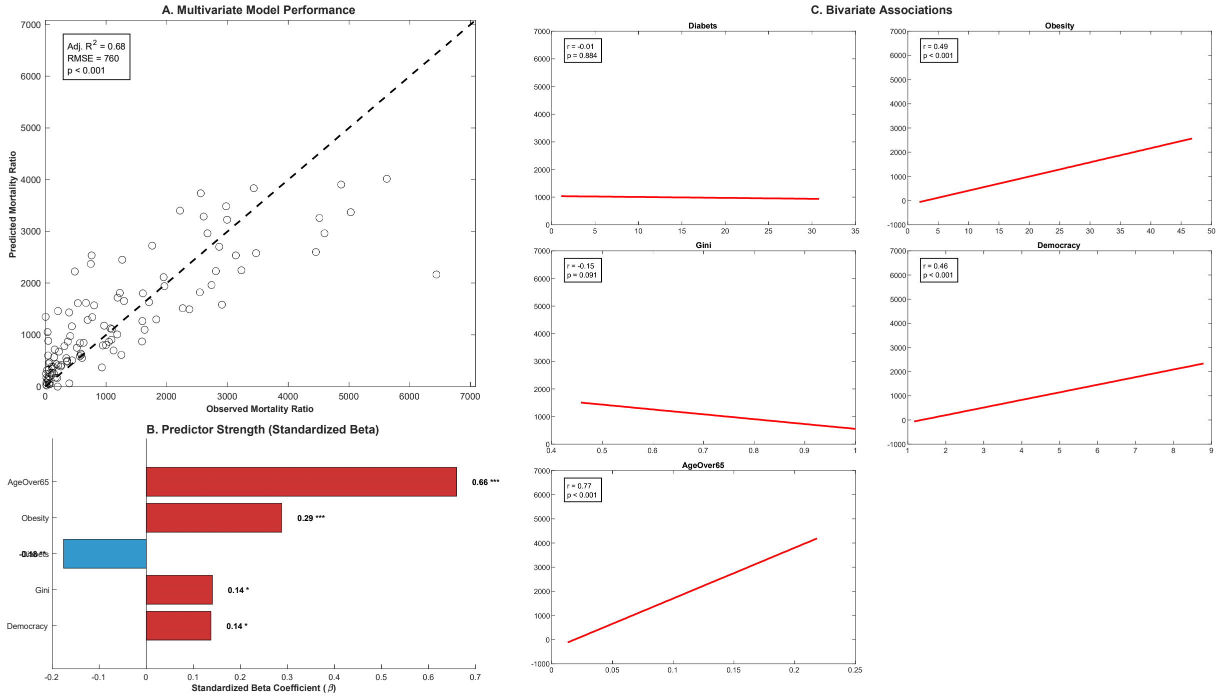

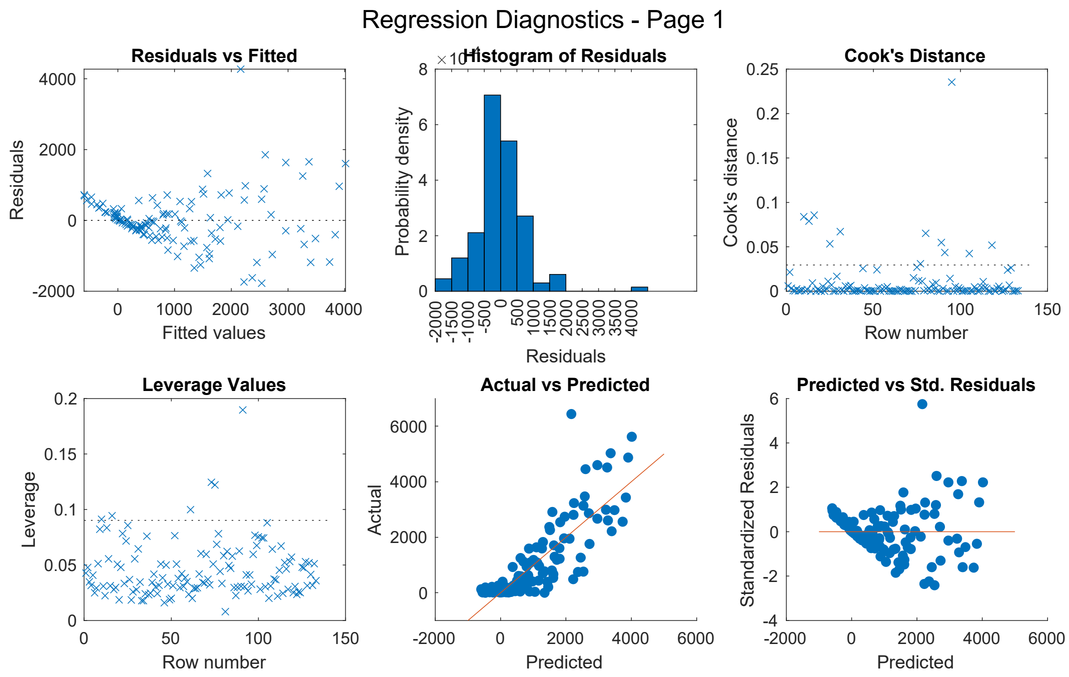

Regression Diagnostics - Page 1
Residuals vs Fitted
Residuals
Fitted values
Histogram of Residuals
Probability density
Residuals
Cook's Distance
Cook's distance
Row number
Leverage Values
Leverage
Row number
Actual vs Predicted
Actual
Predicted
Predicted vs Std. Residuals
Standardized Residuals
Predicted

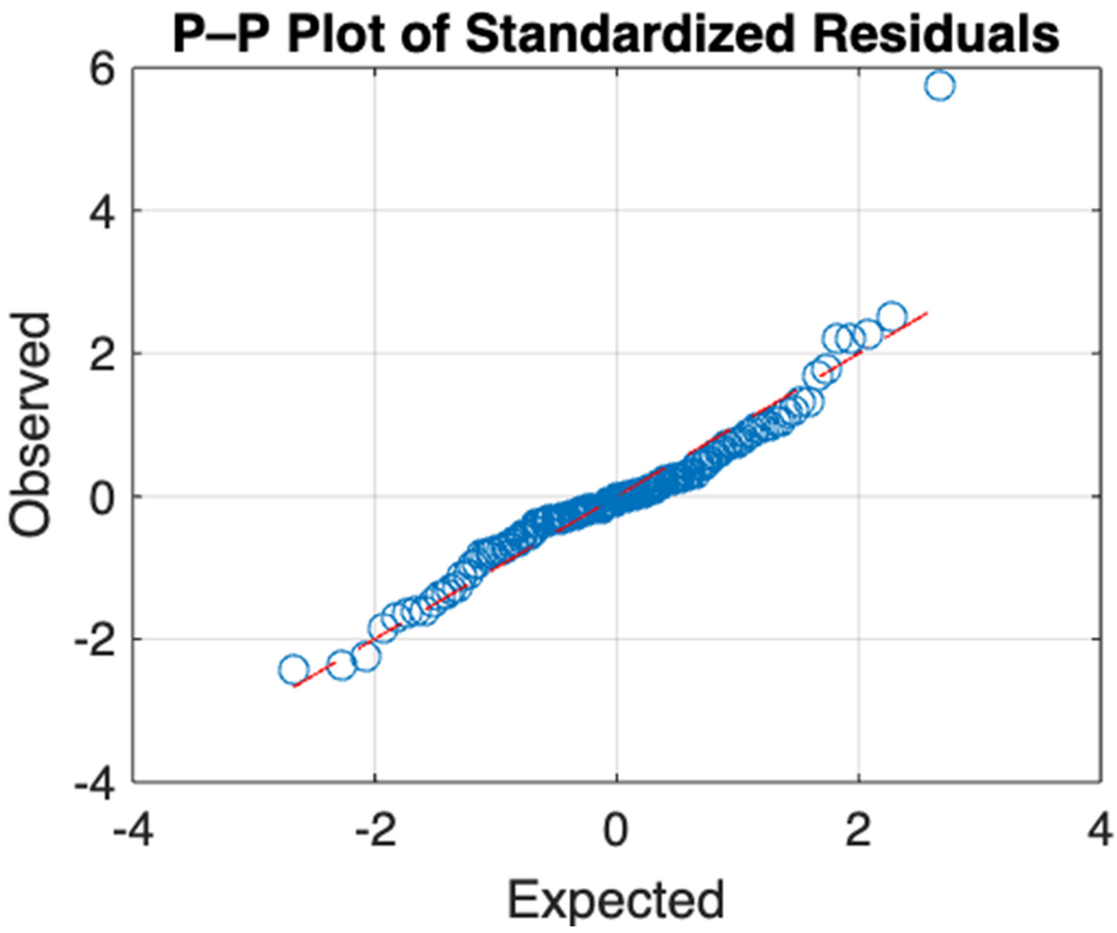

P–P Plot of Standardized Residuals
Observed
Expected

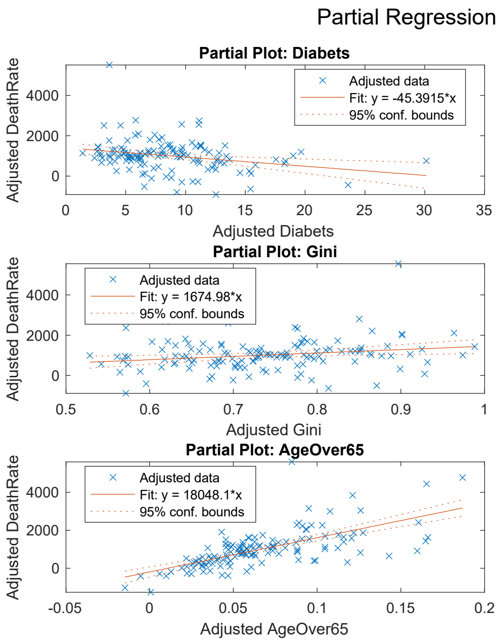

Model Assumption Tests Summary

Durbin-Watson Statistic: 1.824

Breusch–Pagan LM Stat: 15.376 | p-value: 0.0089 → Violated

White Test Stat: 29.199 | p-value: 0.0839 → Satisfied

Variance Inflation Factor (VIF) Values:

Predictor	VIF
Diabets	1.33
Obesity	1.60
Gini	1.24
Democracy	1.24
AgeOver65	1.69

Analysis 8

Linear regression model:
    DeathRate ~ 1 + Diabets + Obesity + AgeOver65

Estimated Coefficients:

	Estimate	SE	tStat	pValue
**(Intercept)**	-105.5	88.778	-1.1883	0.23636
**Diabets**	-25.59	7.5875	-3.3726	0.00092193
**Obesity**	17.997	3.6215	4.9696	1.6263e-06
**AgeOver65**	3792.2	529.26	7.165	2.2588e-11

Number of observations: 174, Error degrees of freedom: 170
Root Mean Squared Error: 451
R-squared: 0.415, Adjusted R-Squared: 0.405
F-statistic vs. constant model: 40.2, p-value = 1.1e-19

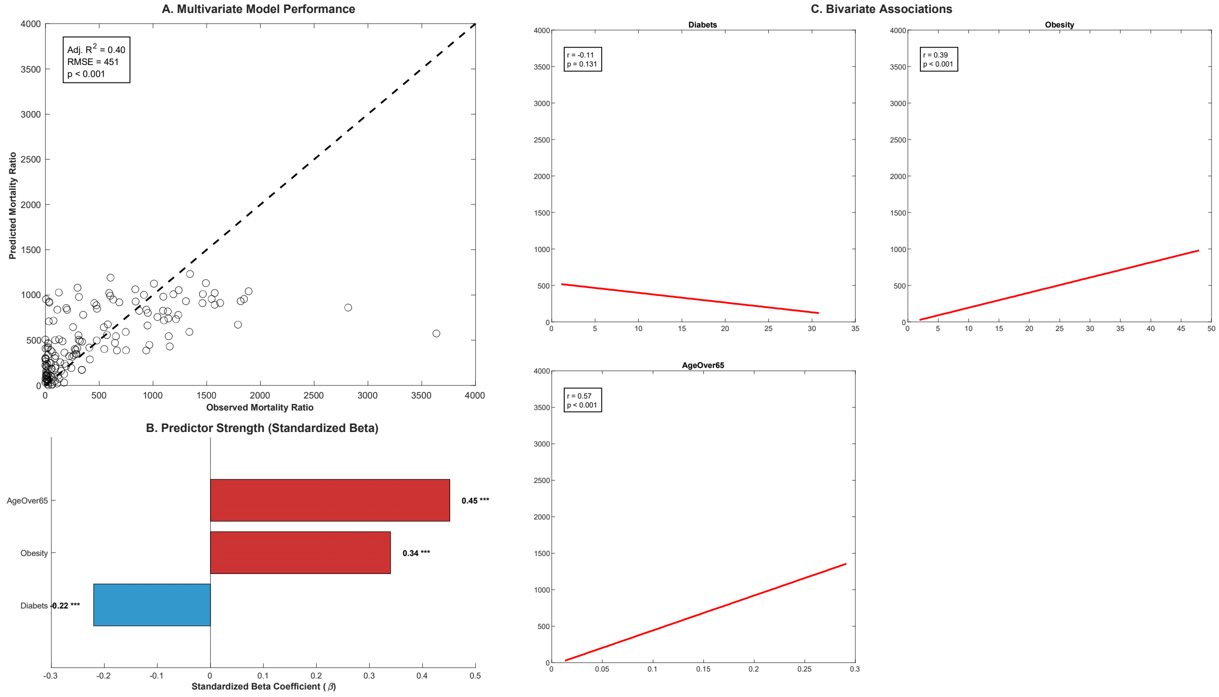

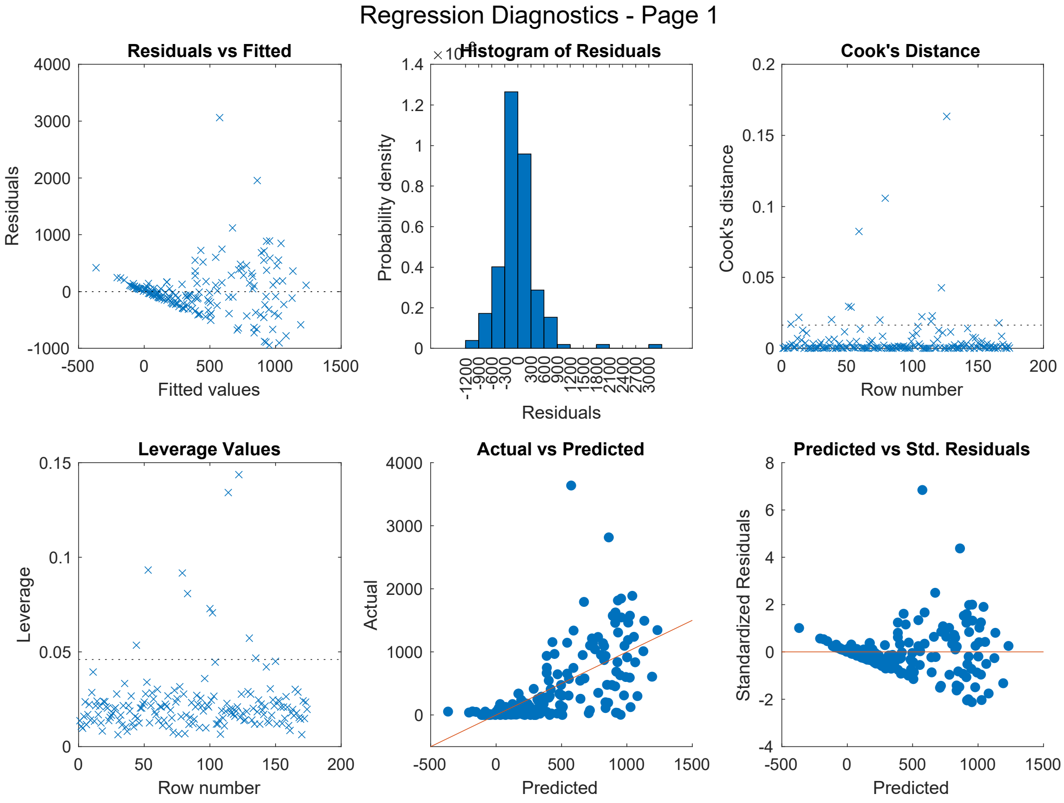
Regression Diagnostics - Page 1
Residuals vs Fitted
Residuals
Fitted values
Histogram of Residuals
Probability density
Residuals
Cook's Distance
Cook's distance
Row number
Leverage Values
Leverage
Row number
Actual vs Predicted
Actual
Predicted
Predicted vs Std. Residuals
Standardized Residuals
Predicted

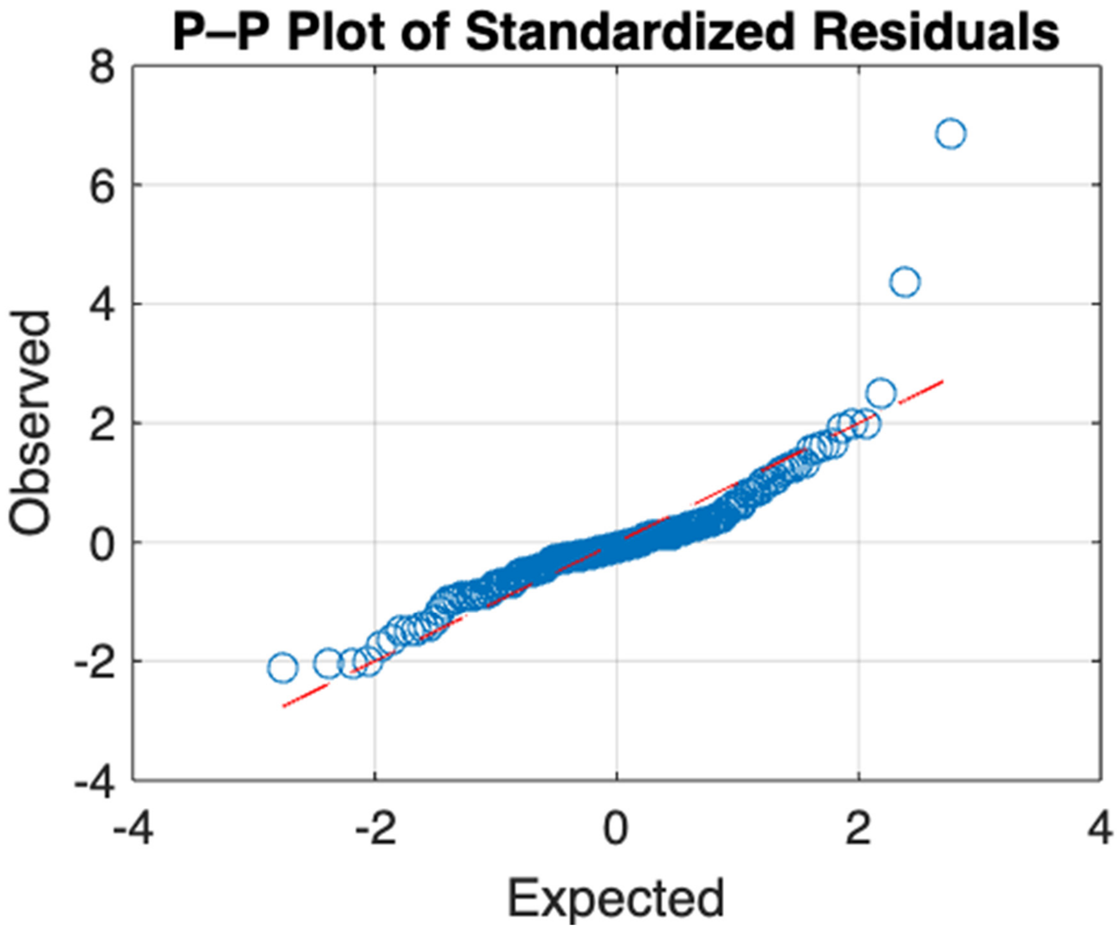
P–P Plot of Standardized Residuals
Observed
Expected
8
6
4
2
0
-2
-4
-4
-2
0
2
4

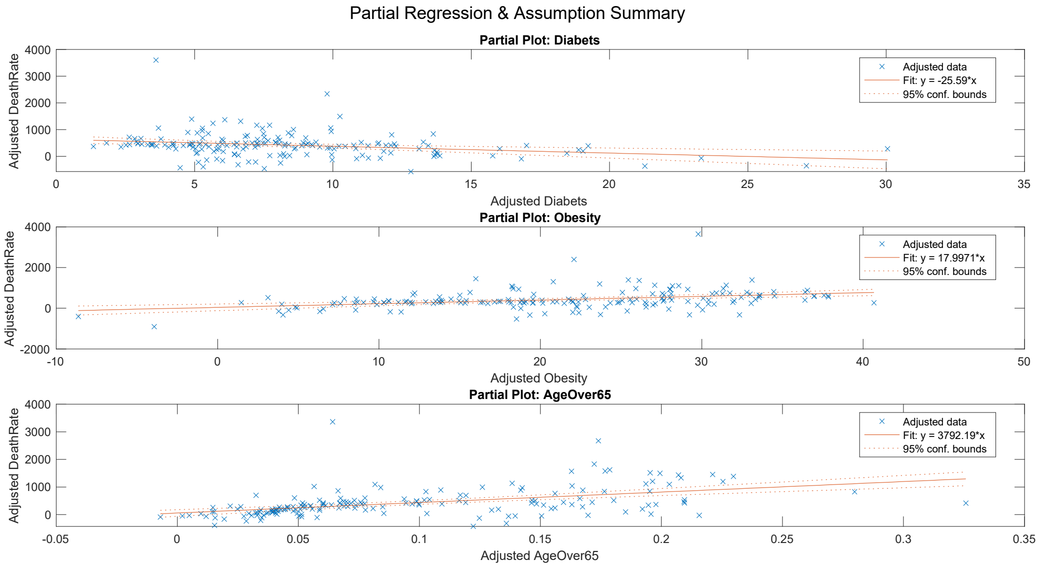

Model Assumption Tests Summary

Durbin-Watson Statistic: 2.185

Breusch–Pagan LM Stat: 7.106 | p-value: 0.0686 → Satisfied

White Test Stat: 8.920 | p-value: 0.4447 → Satisfied

Variance Inflation Factor (VIF) Values:

Predictor	VIF
Diabets	1.24
Obesity	1.36
AgeOver65	1.15

Analysis 9

```
Linear regression model (robust fit):
    DeathRate ~ 1 + Obesity + Democracy + GDP + GII + Hyper + MedianAge

Estimated Coefficients:
```

	Estimate	SE	tStat	pValue
**(Intercept)**	-2402.8	291	-8.257	4.3453e-14
**Obesity**	13.62	3.6989	3.6821	0.00031201
**Democracy**	55.684	19.437	2.8649	0.0047078
**GDP**	-0.011043	0.0020707	-5.333	3.1007e-07
**GII**	2.4261	1.4507	1.6724	0.096325
**Hyper**	26.916	5.8088	4.6336	7.203e-06
**MedianAge**	51.189	6.9202	7.397	6.4433e-12

```
Number of observations: 174, Error degrees of freedom: 167
Root Mean Squared Error: 443
R-squared: 0.61,  Adjusted R-Squared: 0.596
F-statistic vs. constant model: 43.6, p-value = 9.19e-32
```

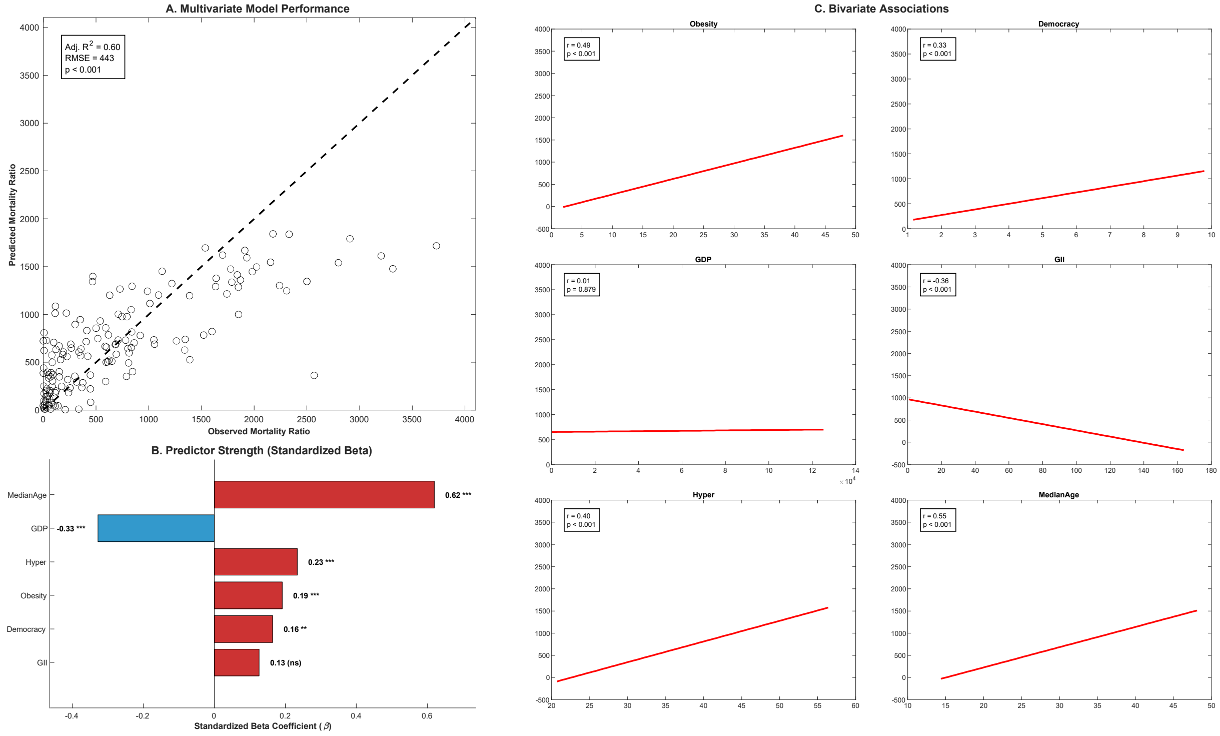

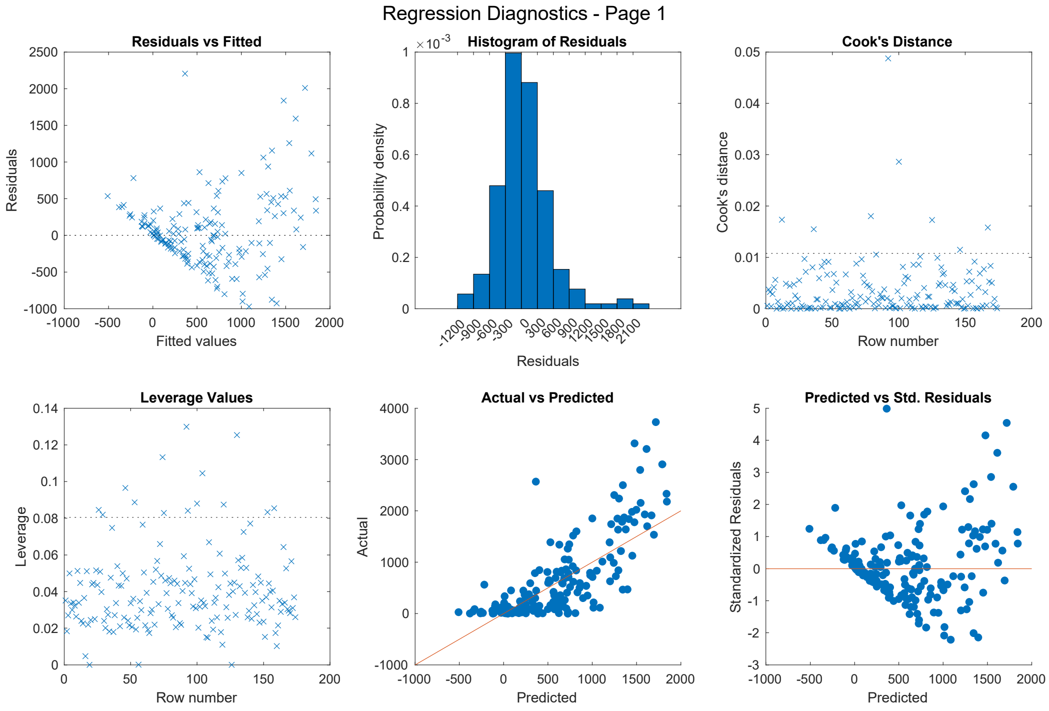
Regression Diagnostics - Page 1
Residuals vs Fitted
Residuals
Fitted values
Histogram of Residuals
×10^-3
Probability density
Residuals
Cook's Distance
Cook's distance
Row number
Leverage Values
Leverage
Row number
Actual vs Predicted
Actual
Predicted
Predicted vs Std. Residuals
Standardized Residuals
Predicted

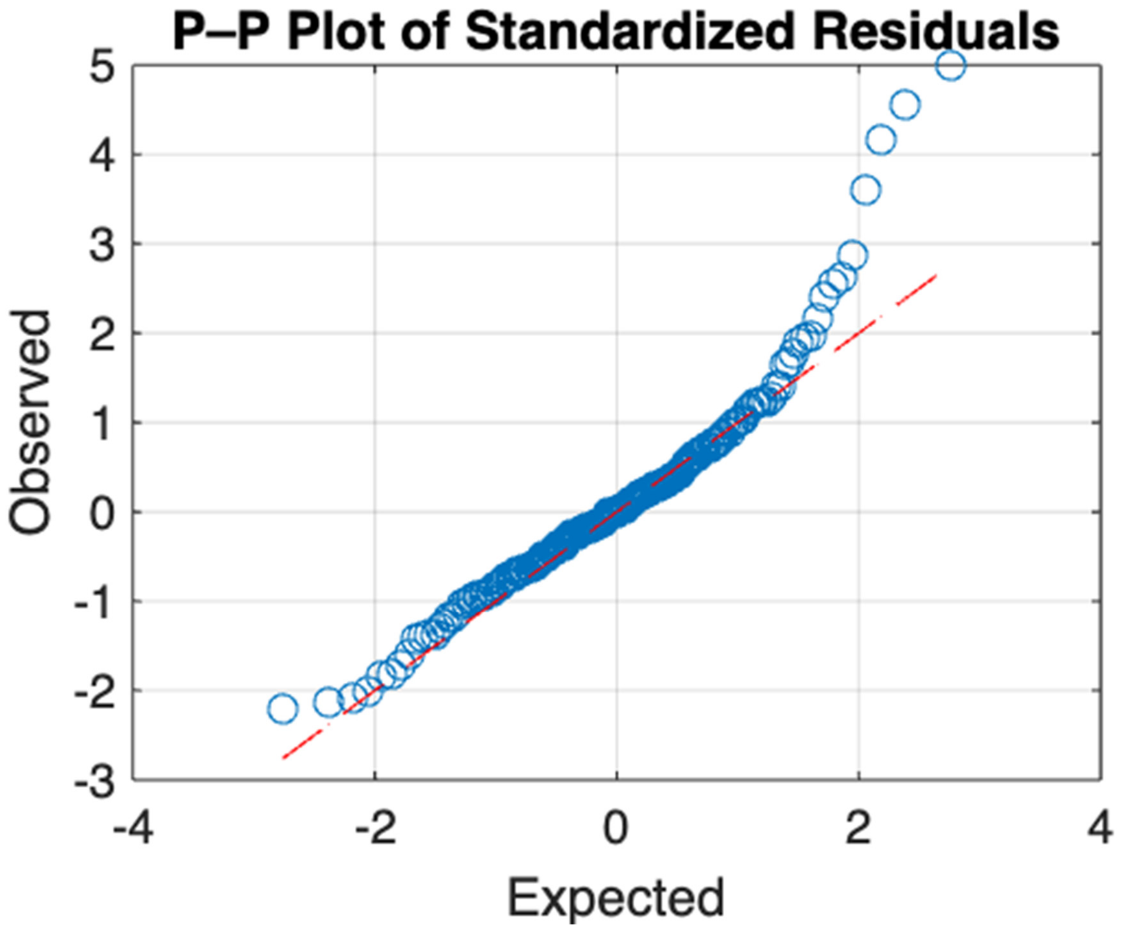
P–P Plot of Standardized Residuals
Observed
Expected

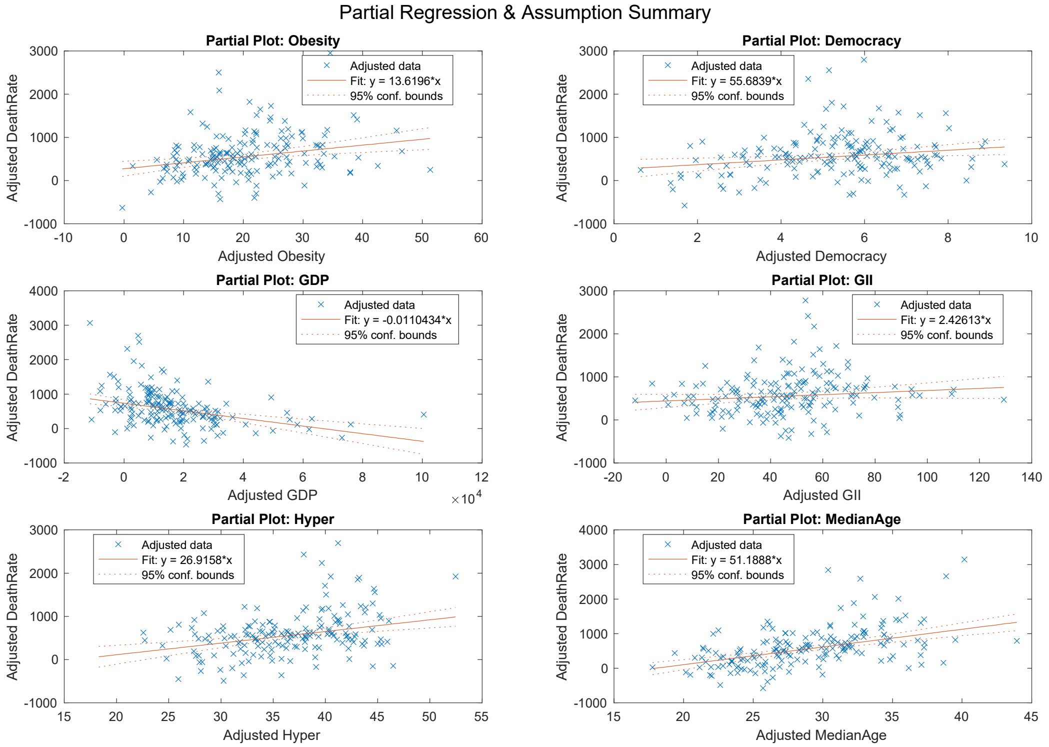

Partial Regression & Assumption Summary
Partial Plot: Obesity
Adjusted data
Fit: y = 13.6196*x
95% conf. bounds
Adjusted DeathRate
Adjusted Obesity
Partial Plot: Democracy
Adjusted data
Fit: y = 55.6839*x
95% conf. bounds
Adjusted DeathRate
Adjusted Democracy
Partial Plot: GDP
Adjusted data
Fit: y = -0.0110434*x
95% conf. bounds
Adjusted DeathRate
Adjusted GDP
×10^4
Partial Plot: GII
Adjusted data
Fit: y = 2.42613*x
95% conf. bounds
Adjusted DeathRate
Adjusted GII
Partial Plot: Hyper
Adjusted data
Fit: y = 26.9158*x
95% conf. bounds
Adjusted DeathRate
Adjusted Hyper
Partial Plot: MedianAge
Adjusted data
Fit: y = 51.1888*x
95% conf. bounds
Adjusted DeathRate
Adjusted MedianAge

Model Assumption Tests Summary

Durbin-Watson Statistic: 1.796

Breusch–Pagan LM Stat: 22.720 | p-value: 0.0009 → Violated

White Test Stat: 57.032 | p-value: 0.0006 → Violated

Variance Inflation Factor (VIF) Values:

Predictor	VIF
Obesity	1.47
Democracy	1.79
GDP	2.06
GII	3.09
Hyper	1.38
MedianAge	3.82

Analysis 10

```
Linear regression model:
    DeathRate ~ 1 + Gini + GDP + AgeOver65

Estimated Coefficients:
```

	Estimate	SE	tStat	pValue
**(Intercept)**	-262.32	99.018	-2.6493	0.0088273
**Gini**	240.09	119.75	2.005	0.046549
**GDP**	0.0021782	0.00064771	3.3629	0.00095288
**AgeOver65**	2367.5	250.1	9.4662	2.4177e-17

```
Number of observations: 174, Error degrees of freedom: 170
Root Mean Squared Error: 160
R-squared: 0.56,  Adjusted R-Squared: 0.552
F-statistic vs. constant model: 72.2, p-value = 3.73e-30

Linear regression model (robust fit):
    DeathRate ~ 1 + Gini + GDP + AgeOver65

Estimated Coefficients:
```

	Estimate	SE	tStat	pValue
**(Intercept)**	-136.49	52.81	-2.5846	0.01059
**Gini**	76.7	63.866	1.201	0.23144
**GDP**	0.0014498	0.00034545	4.197	4.3512e-05
**AgeOver65**	2230	133.39	16.718	9.8761e-38

```
Number of observations: 174, Error degrees of freedom: 170
Root Mean Squared Error: 85.5
R-squared: 0.802,  Adjusted R-Squared: 0.799
F-statistic vs. constant model: 230, p-value = 1.31e-59
```

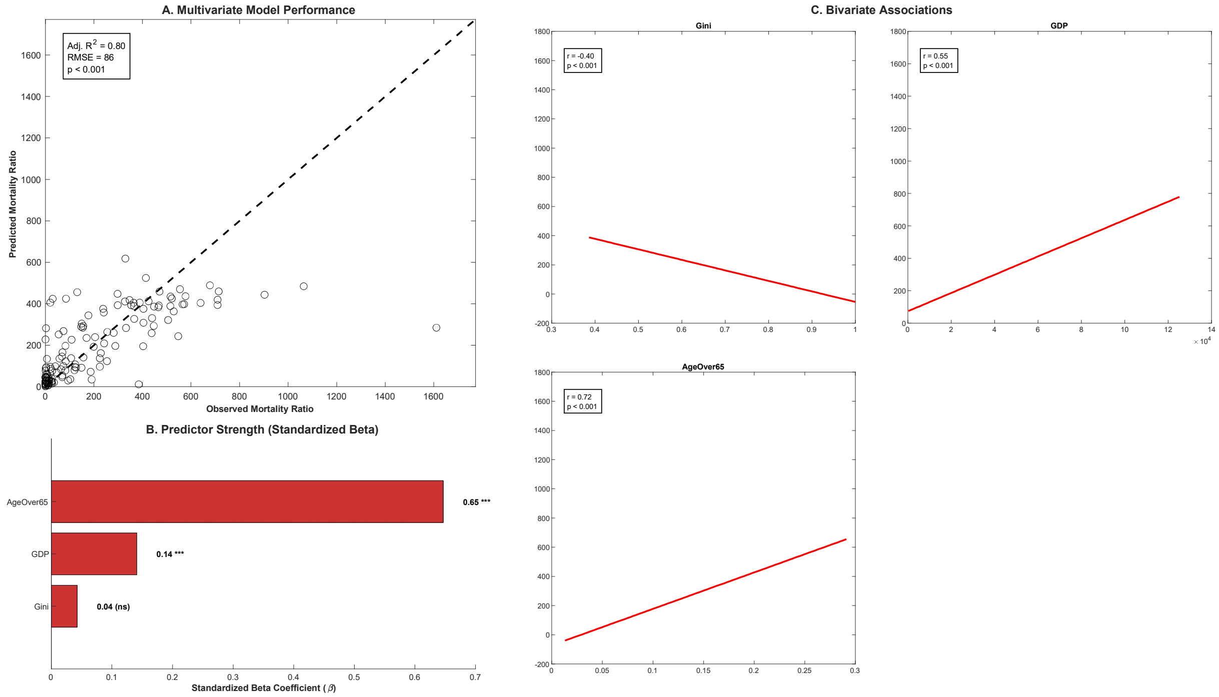
A. Multivariate Model Performance
Adj. R^2 = 0.80
RMSE = 86
p < 0.001
Predicted Mortality Ratio
Observed Mortality Ratio
B. Predictor Strength (Standardized Beta)
AgeOver65
GDP
Gini
0.65 ***
0.14 ***
0.04 (ns)
Standardized Beta Coefficient (β)
C. Bivariate Associations
Gini
r = -0.40
p < 0.001
GDP
r = 0.55
p < 0.001
AgeOver65
r = 0.72
p < 0.001

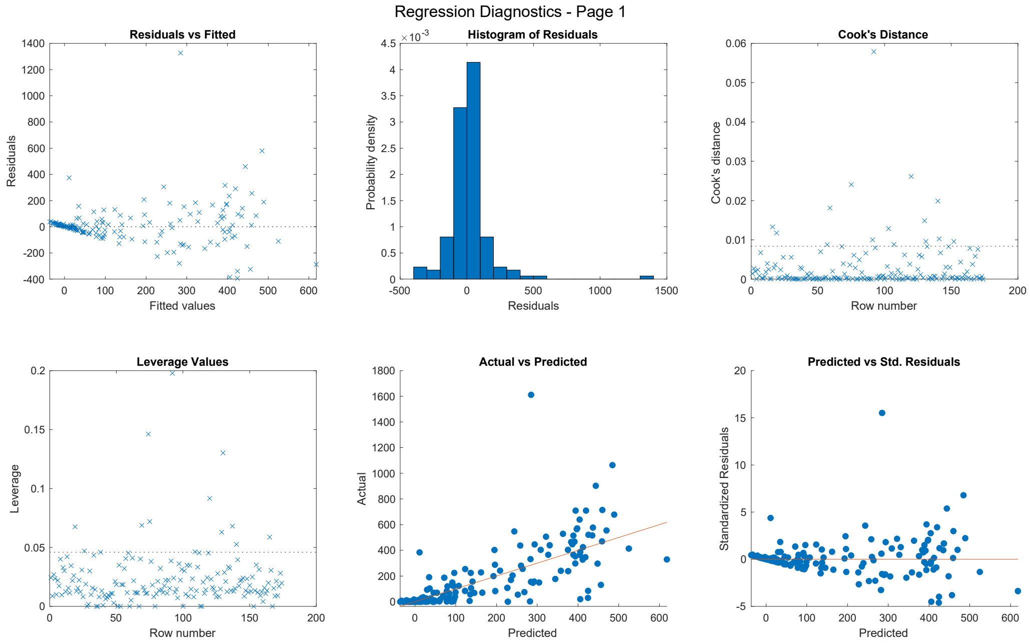
Regression Diagnostics - Page 1
Residuals vs Fitted
Residuals
Fitted values
Histogram of Residuals
Probability density
Residuals
Cook's Distance
Cook's distance
Row number
Leverage Values
Leverage
Row number
Actual vs Predicted
Actual
Predicted
Predicted vs Std. Residuals
Standardized Residuals
Predicted

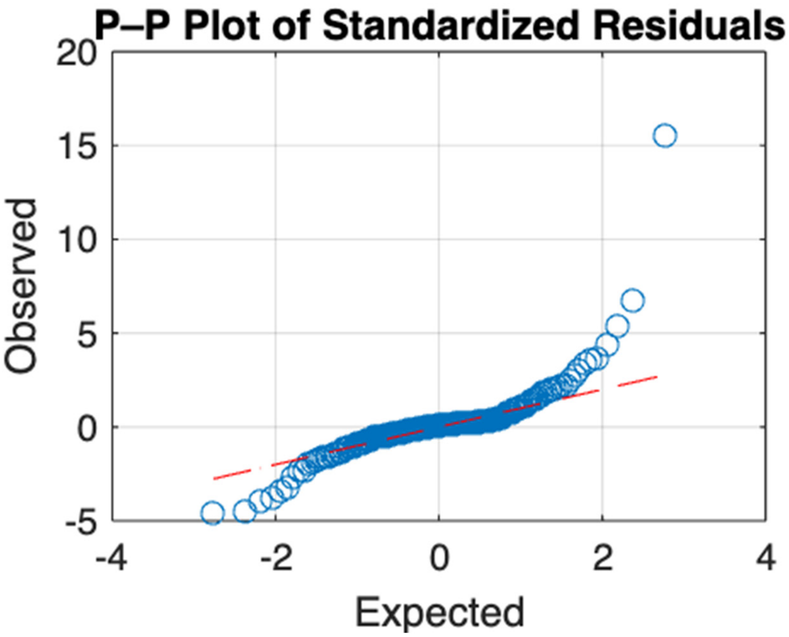
P–P Plot of Standardized Residuals
Observed
Expected
20
15
10
5
0
-5
-4
-2
0
2
4

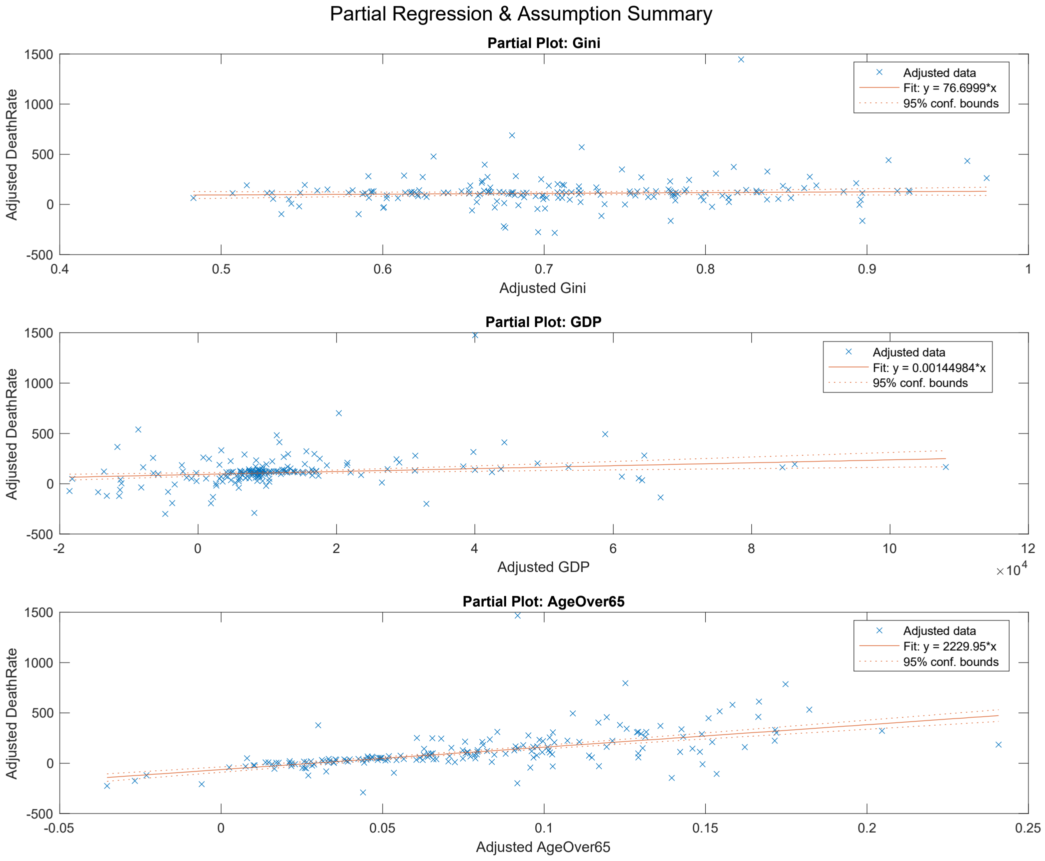
Partial Regression & Assumption Summary
Partial Plot: Gini
Adjusted DeathRate
Adjusted Gini
Adjusted data
Fit: y = 76.6999*x
95% conf. bounds
Partial Plot: GDP
Adjusted DeathRate
Adjusted GDP
Adjusted data
Fit: y = 0.00144984*x
95% conf. bounds
$\times 10^4$
Partial Plot: AgeOver65
Adjusted DeathRate
Adjusted AgeOver65
Adjusted data
Fit: y = 2229.95*x
95% conf. bounds

Model Assumption Tests Summary

Durbin-Watson Statistic: 1.969

Breusch–Pagan LM Stat: 8.408 | p-value: 0.0383 → Violated

White Test Stat: 18.308 | p-value: 0.0318 → Violated

Variance Inflation Factor (VIF) Values:

Predictor	VIF
Gini	1.72
GDP	1.54
AgeOver65	2.03
