# Supplementary figures and images for: An Ecological Study on the Mortality Impact of the COVID-19 Pandemic According to Country Development Status and Pandemic Years
Source: Epidemiologia (Basel). 2026 Apr 6;7(2):50. doi: 10.3390/epidemiologia7020050 (PMC13115391; doi:10.3390/epidemiologia7020050)

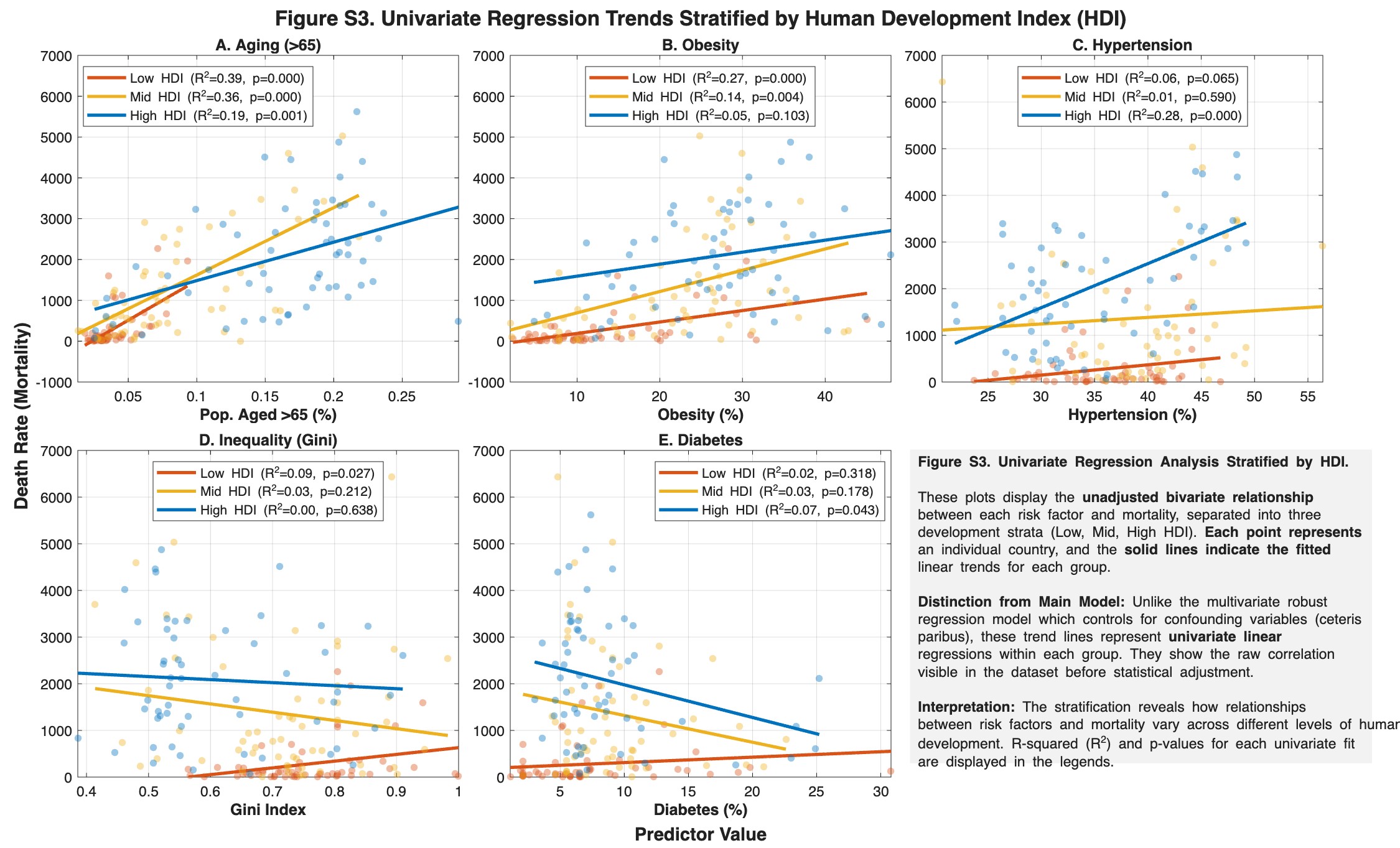

Supplement: Supplementary file 1 [file epidemiologia-07-00050-s001.zip › Figure S3. Univariate Regression Trends Stratified by Human Development Index (HDI).jpg]

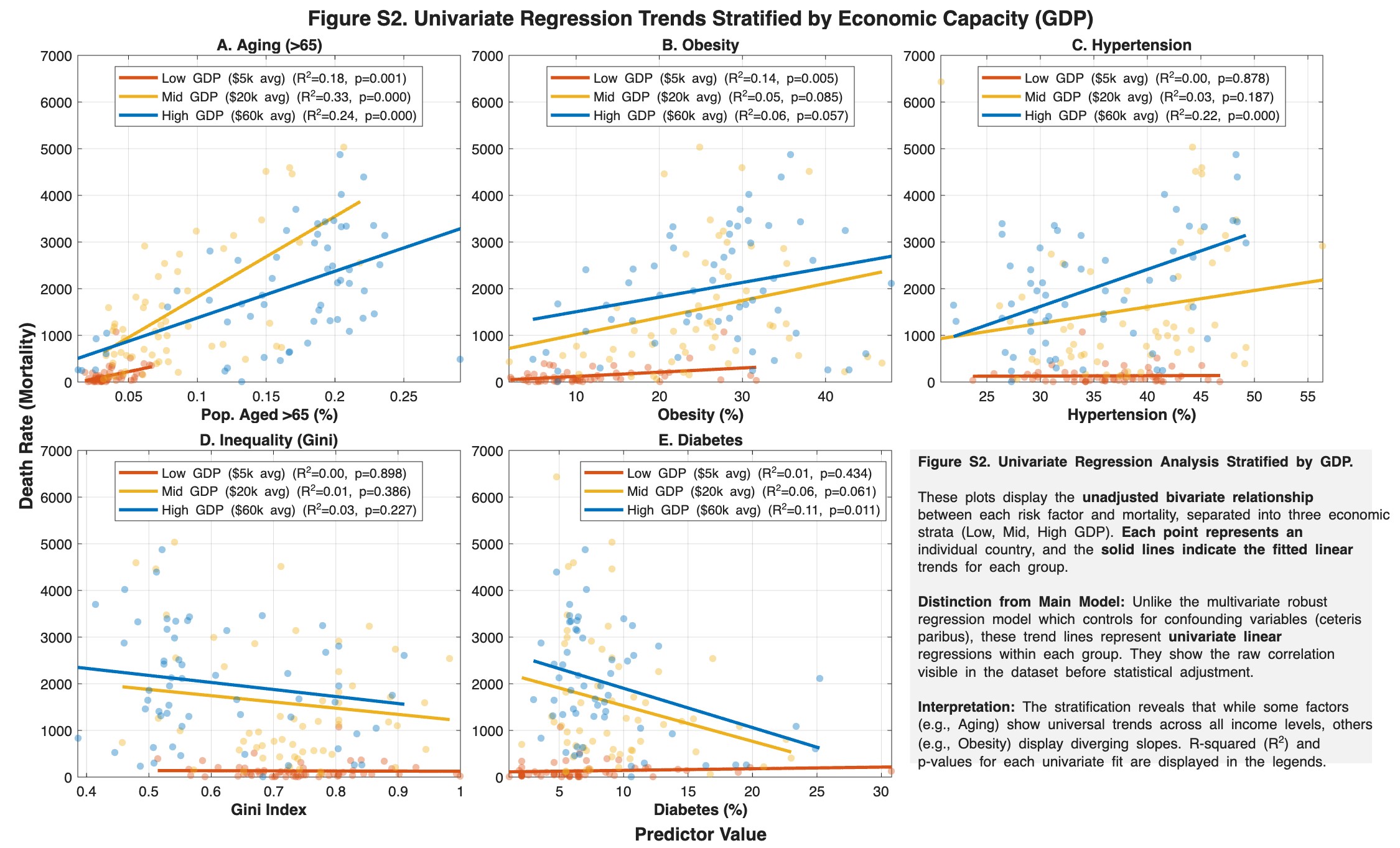

Supplement: Supplementary file 1 [file epidemiologia-07-00050-s001.zip › Figure S2. Univariate Regression Trends Stratified by Economic Capacity (GDP).jpg]

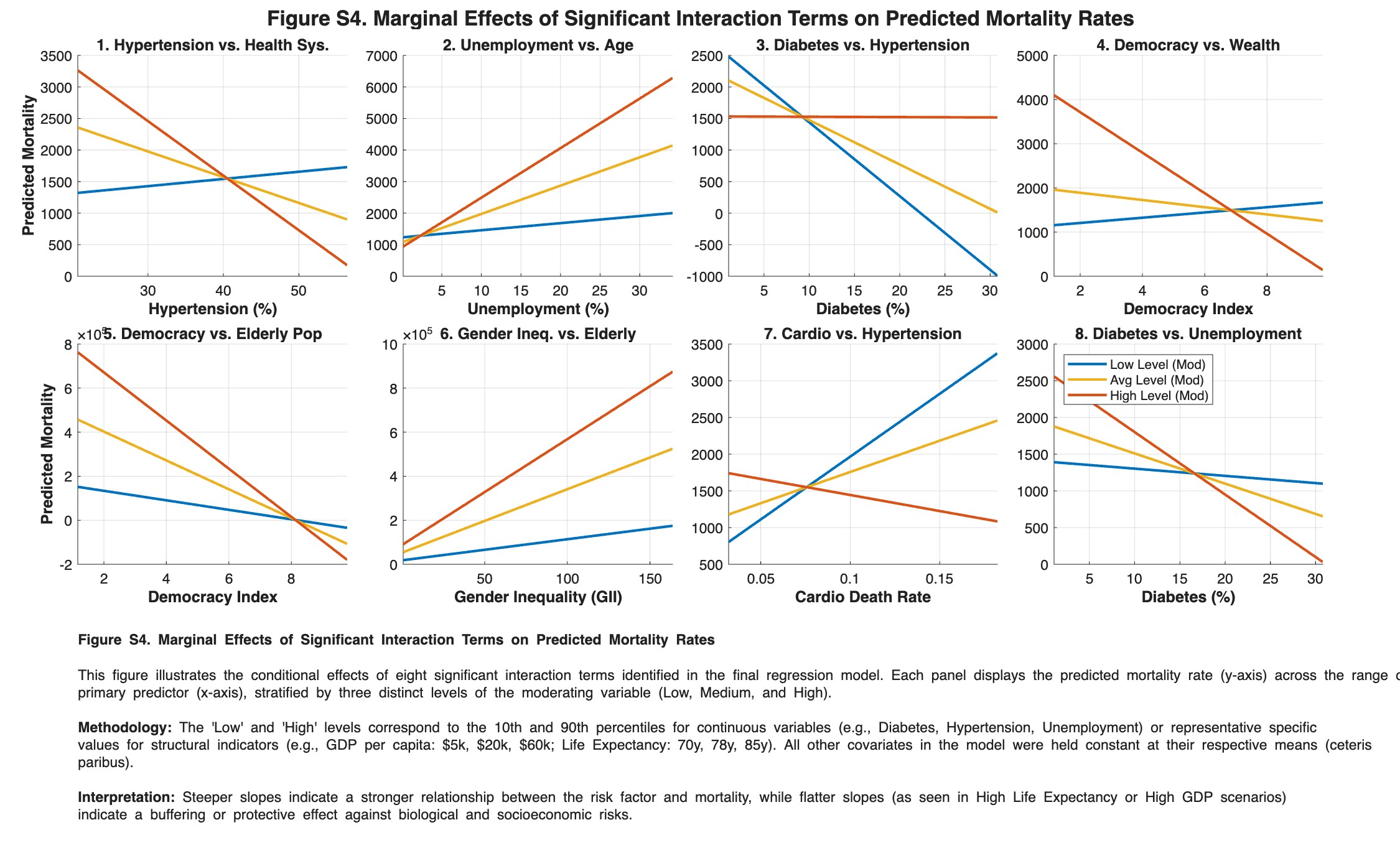

Supplement: Supplementary file 1 [file epidemiologia-07-00050-s001.zip › Figure S4 Marginal Effects of Significant Interaction Terms on Predicted Mortality Rates.jpg]
